# Supplementary material for: A facile way to construct sensor array library via supramolecular chemistry for discriminating complex systems
Source: Nat Commun. 2022 Jul 25;13:4293. doi: 10.1038/s41467-022-31986-x (PMC9314354; doi:10.1038/s41467-022-31986-x)
Supplement: Supplementary file 1 — Supplementary Information [file 41467_2022_31986_MOESM1_ESM.pdf]

Supplementary Information
for
A Facile Way to Construct Sensor Array Library via Supramolecular Chemistry for Discriminating Complex Systems

Jia-Hong Tian [1]‡, Xin-Yue Hu [1]‡, Zong-Ying Hu [1], Han-Wen Tian [1], Juan-Juan Li [1], Yu-Chen Pan [1], Hua-Bin Li [1] & Dong-Sheng Guo [1]*

[1] College of Chemistry, Key Laboratory of Functional Polymer Materials (Ministry of Education), State Key Laboratory of Elemento-Organic Chemistry, Nankai University, Tianjin 300071, China.

* email: dshguo@nankai.edu.cn

‡ These authors contributed equally.

Supplementary Note 1. Syntheses of compounds.

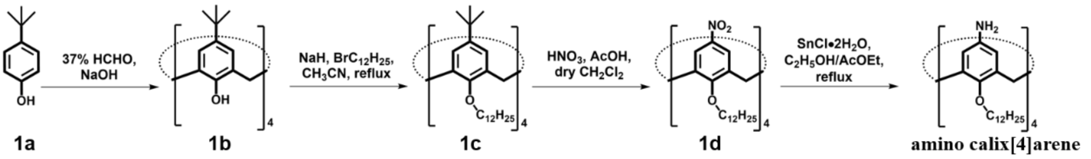

Supplementary Figure 1. The synthetic route of amino calix[4]arene.

Amino calixarenes were synthesized and purified according to the literature procedures.[1–4] **1b**: A mixture of *p*-tert-butylphenol (40.0 g, 0.27 mol), 37% formaldehyde solution (1.2 eq), and sodium hydroxide (0.05 eq) of were placed in a three-necked, round-bottomed flask equipped with a mechanical stirrer. The contents of the open flask were heated for 1.5 h at 110 °C by means of a heating mantle. Stirring was discontinued, the reaction mixture was allowed to cool to room temperature. To dissolve the residue 400 mL of warm diphenyl ether was added to the flask and the contents were stirred. The contents of the flask were stirred and heated to 160 °C for 20 min and then heated to 255 °C for 2 h under argon. The reaction mixture was cooled to room temperature, and the product was precipitated by the addition of 600 mL of ethyl acetate. The resulting mixture was stirred for 30 min and allowed to stand. Filtration yields material that was washed with ethyl acetate, acetic acid, and water. Yield: 69%. **1c**: A stirred mixture of **1b** (10.0 g, 15.4 mmol), 1-bromododecane (3 eq per phenolic hydroxyl group) and sodium hydride (3 eq per phenolic hydroxyl group) in dry DMF (100 mL) was refluxed for 12 h. Under ice bath conditions, slowly add water dropwise to the reaction solution, quench A until no bubbles were generated. The solvent was treated with DCM (400 mL) and washed with 1 M hydrochloric acid, dried over Na_2SO_4, and concentrated to dryness. Crude product was purified by DCM/methanol to afford colorless crystals **1c**. Yield: 80%. **1d**: To a solution of **1c** (5.0 g, 3.8 mmol) in dry DCM (100 mL) and acetic acid (40 mL), HNO_3 (12 mL) was added gradually and the mixture was stirred for about 1−4 h at room temperature. The color of the mixture changed from dark purple to orange. Then water (250 mL) was added into the reaction mixture and followed by stirring for 30 min. The mixture was washed with saturated Na_2CO_3, brine and water. After drying with anhydrous Na_2SO_4, the solvent was removed in vacuo. The residue was recrystallized from DCM/methanol to obtain yellow solid **1d**. Yield: 85%. Amino calix[4]arene: To a solution of **1d** (1.0 g, 0.8 mmol) in dry ethanol/ethyl acetate (80 mL/80 mL), $SnCl_2{\cdot}2H_2O$ (6 eq each nitro group) was added and the mixture was refluxed for 48 h. Then the mixture was poured into ice water. After all the ice melt, sodium hydroxide was added to the mixture to adjust the pH to 8.0. DCM was added and the mixture was stirred overnight at room temperature. The solution was washed by water for three times. After drying with anhydrous Na_2SO_4, the solvent was removed in vacuo to obtain white solid amino calix[4]arene. Yield: 75%.

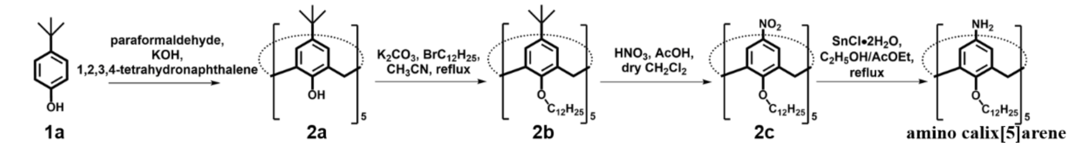

Supplementary Figure 2. The synthetic route of amino calix[5]arene.

2a: *p*-tert-butylphenol (75.0 g, 2.5 mol), paraformaldehyde (1.1 eq), KOH (0.3 eq) in

34 mL of water, and 1.0 L of tetralin were stirred in a 2 L, 3 necked, round-bottomed flask equipped with a mechanical stirrer, a Dean-Stark water trap fitted with a condenser, a nitrogen inlet and a thermometer. The mixture was heated to 80−85 °C and held at that temperature for 1.5 h under a small positive pressure of nitrogen. During this time the reaction mixture became turbid and changed to a lemon-yellow color. The heat was increased to rapidly bring the temperature to 180−185 °C, and the nitrogen flow was increased to facilitate the removal of water. During this heating period, the color of the reaction mixture changed from a bright, lemon-yellow to a light yellow and finally to a dark, red-brown, as 40−50 mL of formalin was collected. Once a temperature of 180−185 °C was reached, the reaction mixture was held at that temperature for 10 min, and the heating mantle was reduced the temperature to 160−165 °C. The reaction mixture was held within this range for 3 h and then allowed to cool to room temperature. When cooled, the reaction mixture was filtered and the precipitate was an off-white powder. The filtrate was evaporated to dryness in vacuo and the residual dark-brown, gummy residue was stirred vigorously with 400 mL of chloroform and 200 mL of 1 M hydrochloric acid for 15 min. The organic solvent was evaporated under reduced pressure. 250 mL of ethanol was added to the obtained viscous liquid, then refluxed for 30 min, and stirred at room temperature for 1 h. A large amount of khaki solid was precipitated in the solution, which was obtained by suction filtration. Vacuum drying to remove residual ethanol in the solids. The residue was triturated by refluxing for 0.5 h with 400 mL of acetone and filtered hot to leave white powder. The filtrate was concentrated to 250–300 mL and allowed to cool to room temperature. After a few hours at room temperature, a small amount of white powder sometimes formed and was removed by filtration. **2a** was collected as large, diamond-shaped crystal. Yield: 12%. **2b**: A stirred mixture of **2a** (5.0 g, 6.2 mmol), 1-bromododecane (3 eq per phenolic hydroxyl group) and K_2CO_3 (8 eq per phenolic hydroxyl group) in dry CH_3CN (100 mL) was refluxed for 12 h. After cooling, the solvent was evaporated under reduced pressure to give a residue, which was treated with DCM (200 mL). The inorganic salts were filtered off and washed with DCM. The combined organic layers were washed with 1 M hydrochloric acid, dried over Na_2SO_4, and concentrated to dryness. Crude product was purified by recrystallization with ethanol gave a precipitate, which was collected by suction filtration and recrystallized from DCM/ethanol to afford colorless crystals **2b**. Yield: 87%. **2c**: The synthesis and purification methods refer to the experimental steps of **1d**. Yield: 82%. amino calix[5]arene: The synthesis and purification methods refer to the experimental steps of amino calix[4]arene. Yield: 72%.

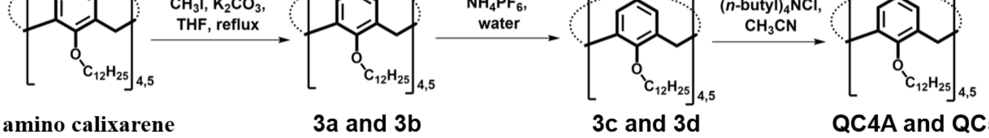

Supplementary Figure 3. The synthetic route of QC4A and QC5A.

General procedure for syntheses of QC4A and QC5A: To the solution of amino calixarene in THF, iodomethane (10 eq per amino group) and K_2CO_3 (40 eq) were added and heated to reflux for 3 d. After cooling to room temperature, the solid was filtered

and dispersed in water and then in ethanol to get rid of the impurity. The iodo-QC4A or iodo-QC5A was dissolved in hot water and 10 eq NH_4PF_6 was added. The solid was filtered and dissolved in acetonitrile. 10 eq (*n*-butyl)$_4$NCl was added and the solution was refluxed for 3 h. The final product was obtained by filter and dried in vacuum. The overall yield is 86% for QC4A and 78% for QC5A.

QC4A: ^{1}H NMR (400 MHz, DMSO-d_6, δ): 7.64 (s, 8H, Ar-H), 4.39 (d, J = 12.7 Hz, 4H, Ar-CH_2-Ar), 3.88 (t, J = 7.3 Hz, 8H, O-CH_2-), 3.50 (d, J = 12.8 Hz, 4H, Ar-CH_2-Ar), 3.43 (s, 36H, N-CH_3), 2.05−1.89 (m, 8H, alkyl-CH_2-), 1.39−1.25 (m, 72H, alkyl-H-CH_2-), 0.85 (t, J = 6.6 Hz, 12H, alkyl-CH_3) ppm.

^{13}C NMR (100 MHz, DMSO-d_6, δ): 156.63, 142.39, 136.02, 120.70, 76.27, 57.33, 31.88, 30.42, 30.18, 30.08, 29.99, 29.86, 29.77, 29.36, 26.21, 22.61, 14.35 ppm.

MS (ESI): $[M]^{4+}$: m/z calcd. for ($C_{88}H_{152}N_4O_4^{4+}$): 332.29, found: 332.29713.

M. p.:> 300 °C

Elemental Analysis (%): ($C_{88}H_{152}N_4O_4Cl_4 \cdot H_2O$) Calcd. C 70.94, H 10.42, N 3.76, found C 70.82, H 10.51, N 3.72.

QC5A: ^{1}H NMR (400 MHz, DMSO-d_6, δ): 8.17 (s, 10H, Ar-H), 4.49 (d, J = 13.1 Hz, 5H, Ar-CH_2-Ar), 3.89 (t, J = 8 Hz, 10H, O-CH_2-), 3.58 (s, 45H, N-CH_3), 2.02 (s, 10H, alkyl-CH_2-), 1.41 (m, 10H, alkyl-CH_2-), 1.26 (m, 80H, alkyl-H-CH_2-), 0.85 (t, J = 6.6 Hz, 15H, alkyl-CH_3) ppm.

^{13}C NMR (100 MHz, DMSO-d_6, δ): 156.31, 142.28, 135.35, 121.17, 74.87, 56.52, 31.89, 30.36, 30.21, 30.03, 29.89, 29.78, 29.37, 26.29, 22.48, 13.17 ppm.

MS (ESI): $[M]^{5+}$: m/z calcd. for ($C_{110}H_{190}N_5O_5^{5+}$): 332.29, found: 332.29800.

M. p.:> 300 °C

Elemental Analysis (%): ($C_{110}H_{190}N_5O_5Cl_5 \cdot H_2O$) Calcd. C 71.11, H 10.42, N 3.77, found C 71.26, H 10.38, N 3.74.

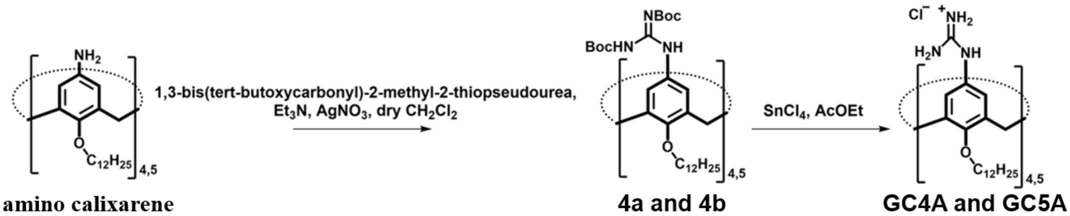

Supplementary Figure 4. The synthetic route of GC4A and GC5A.

GC4A and GC5A were synthesized and purified according to the literature procedures.[3–4] **4a and 4b**: To a solution of amino calixarene in dry DCM (25 mL), 1,3-bis(tert-butoxycarbonyl)-2-methyl-2-thiopseudourea (1 eq per amino group), $AgNO_3$ (1.2 eq per amino group) and Et_3N (2 eq per amino group) were added and the mixture was stirred for 48 h at room temperature. The solvent was evaporated under reduced pressure and the residue was purified by column chromatography on silica gel to obtain white powder (**4a** and **4b**). GC4A and GC5A: $SnCl_4$ (0.2 mL) was added to a solution of **4a** or **4b** (0.3 mmol) in 20 mL of ethyl acetate. The mixture was stirred for 8 h at room temperature and the solvent evaporated under reduce pressure. The residue was dissolved in methanol, then large amount of diethyl ether was added to obtain white precipitation. The yield is for 69% GC4A and 65% for GC5A. The characterization matches well with the reported data.

GC4A: ^{1}H NMR (400 MHz, CD_3OD, δ): 6.68 (s, 8H, Ar-H), 4.51 (d, J = 12 Hz, 4 H, Ar-CH_2-Ar), 3.97 (t, J = 8.0 Hz, 8H, -CH_2-O-Ar), 3.28 (br. s., 2 H, Ar-CH_2-Ar), 2.03−1.96 (m, 8H, -CH_2-CH_2-O-Ar), 1.45−1.31 (m, 72H, alkyl-CH_2-), 0.91 (t, J = 6.8 Hz, 12H, alkyl-CH_3) ppm.

GC5A: ^{1}H NMR (400 MHz, CD_3OD, δ): 7.01 (s, 10H, Ar-H), 4.61 (d, J = 16 Hz, 5H, Ar-CH_2-Ar), 3.91 (t, J = 8.0 Hz, 10H, -CH_2-O-Ar), 3.47 (d, J = 16 Hz, 5H, Ar-CH_2-Ar), 2.04−2.01 (m, 10H, -CH_2-CH_2-O-Ar), 1.55−1.31 (m, 90H, alkyl-CH_2-), 0.92−0.89 (m, 15H, alkyl-CH_3) ppm.

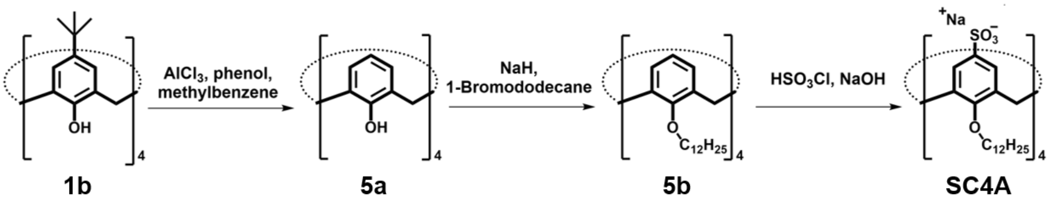

Supplementary Figure 5. The synthetic route of SC4A.

SC4A was synthesized and purified according to the literature procedures.[5–6] **5a**: **1b** (13.3 g, 20.5 mmol), anhydrous aluminium chloride (14.0 g, 105.3 mmol), phenol (9.1 g, 96.7 mmol) and methylbenzene (125 mL) were added to a round bottom flask. The mixture was heated to 40 °C for 1 h under argon. The reaction was quenched by the addition of dilute hydrochloric acid. After stirring for 30 min, the reaction mixture was separated. The organic phase was concentrated and methanol was added. After suction filtration, the residue was recrystallized from chloroform/methanol. Yield: 69%. **5b**: Under nitrogen atmosphere and magnetic stirring, in dry DMF, was added sodium hydride (0.7 g, 27.0 mmol) and **5a** (1.1 g, 2.7 mmol). After stirring, 1-bromododecane (6.7 g, 27.0 mmol) was added slowly. After 12 hours, the sodium hydride was neutralized with 14 mL of hydrochloric acid (2 M), the white precipitate was filtered and recrystallized from chloroform/methanol. Yield: 93%. SC4A: A solution of **5b** (2.0 g, 1.82 mmol) in 50.0 mL of chloroform was stirred under argon at ice bath temperature for 30 min. Then 0.5 mL (7.50 mmol, dissolved in 60.0 mL of chloroform) of HSO_3Cl was added. The mixture was stirred at 0 °C for another 3 h and then concentrated. The residue was dissolved in 100.0 mL of anhydrous ethanol. After a solution of sodium hydroxide (360 mg, 9.00 mmol) in anhydrous ethanol was then added, the mixture was stirred for 30 min. After centrifugation, the resulting white precipitate was recrystallized from water. Yield: 8%. The characterization matches well with the reported data.

^{1}H NMR (400 MHz, DMSO-d_6, δ): 7.08 (s, 8H; Ar-H), 4.32 (d, J = 12.1 Hz, 4H; Ar-CH_2-Ar), 3.86 (t, J = 7.4 Hz, 8H; -CH_2-O-Ar), 3.22 (d, J = 12.1 Hz, 4H; Ar-CH_2-Ar), 1.92 (m, 8H; -CH_2-CH_2-O-Ar), 1.45−1.20 (m, 72H; alkyl-CH_2-), 0.85 (t, J = 6.5 Hz, 12H; alkyl-CH_3) ppm.

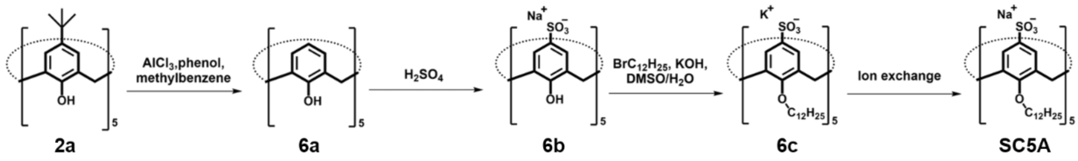

Supplementary Figure 6. The synthetic route of SC5A.

SC5A was synthesized and purified according to the literature procedures.[7] **6a**: **2a** (1.9 g, 2.3 mmol), anhydrous aluminium chloride (2.27 g, 17.0 mmol), phenol (1.69 g, 18.0 mmol) and methylbenzene (30 mL) were added to a round bottom flask. The reaction mixture was reacted at room temperature for 3 h under argon. The reaction mixture was quenched by the addition of dilute hydrochloric acid. After stirring for 30 min, the reaction mixture was separated. The organic phase was concentrated and methanol was added. After suction filtration, the pale-yellow powder was purified by column chromatography (petroleum ether/DCM = 2:1). The product is white solid. Yield: 49%. **6b**: **6a** (1.0 g, 1.9 mmol) was added to a round bottom flask, sulfuric acid (6 mL) was added dropwise and the mixture was reacted at 80−90 °C for 4 hours. After cooling to room temperature, the reaction mixture was dropped into diethyl ether (400 mL) with rapid stirring. The off-white solid obtained by suction filtration was dissolved in a small amount of water, sodium hydroxide (386 mg) was added. Ethanol was added with stirring, and after suction filtration, the residue was recrystallized from distilled water/ethanol. 1.5 g of white crystals were obtained. Yield: 69%. SC5A: **6b** (2.2 g, 1.9 mmol) was mixed with KOH (5.4 g, 96.2 mmol) and1-bromododecane (23.9 g, 96.0 mmol) in dimethyl sulfoxide (60 mL) and water (15 mL), the reaction mixture was heated to 100 °C for 7 days. After cooling to room temperature, the precipitate was recovered by filtration and dissolved in water (10 mL). The insoluble material was removed by filtration, and the product was precipitated from the filtrate by diluting with ethanol. The crude product was dissolved in hot water and then guanidine hydrochloride was added under vigorous string. The precipitate was recovered by filtration after 30 min. The obtained white solid was dissolved in ethanol and then sodium ethylate was added under vigorous string. The product of sodium salt was recovered by filtration after 30 min. Yield: 8%. The characterization matches well with the reported data.

^{1}H NMR (400 MHz, DMSO-d_6, δ): 7.37 (s, 10H; Ar-H), 4.45 (d, J = 13.9 Hz, 5H; Ar-CH_2-Ar), 3.80 (s, 10H; -CH_2-O-Ar), 1.96 (m, 10H; -CH_2-CH_2-O-Ar), 1.49−1.15 (m, 90H; alkyl-CH_2-), 0.85 (t, J = 6.8 Hz, 15H; alkyl-CH_3) ppm.

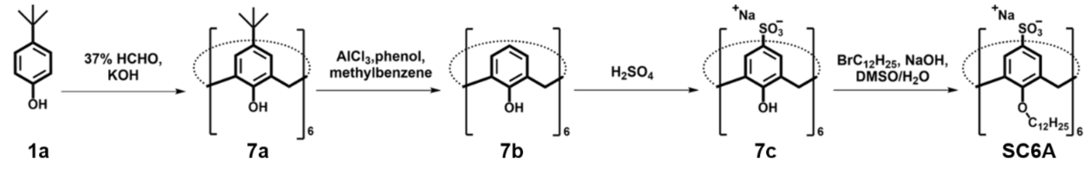

Supplementary Figure 7. The synthetic route of SC6A.

SC6A was synthesized and purified according to the literature procedures.[8–9] **7a**: To the three-necked, round-bottomed flask were added **1a** (100.0 g, 0.7 mol), 135 mL of 37% formaldehyde solution, and potassium hydroxide pellets (15.0 g, 0.2 mol). The reaction mixture was heated and stirred for 2 h under argon. The bright lemon-yellow solution

turned to a thick, golden-yellow mass of taffy like consistency. Xylene (1 L) was added to the flask to dissolve the semisolid mass. Then, reflux by increasing the temperature of the heating mantle for 3 h. After the mixture cooling to room temperature, the mixture was filtered, and the precipitate was washed with xylene and dried on a Büchner funnel to yield crude. This material was dissolved in 2.5 L of chloroform, and treated with 800 mL of 1 M hydrochloric acid. The mixture was transferred to a separatory funnel. The chloroform layer was drawn off, the aqueous layer was extracted with an additional 250 mL of chloroform, and the combined chloroform solution was concentrated to 1 L by boiling, and 1 L of hot acetone was added to the boiling chloroform solution. The mixture was allowed to cool and was then filtered to give 95 g (88%) of product as a white powder. **7b**: The synthesis and purification methods refer to the experimental steps of **6a**. The product of white solid was in 85% yield. **7c**: The synthesis and purification methods refer to the experimental steps of **6b**. The product of white solid was in 75% yield. SC6A: **7c** (3.0 g, 2.4 mmol) was mixed with sodium hydroxide (2.35 g, 58.8 mmol) and 1-bromododecane (14.8 mL) in dimethyl sulfoxide (60 mL) and water (15mL). The reaction mixture was heated at 100 °C for 5 days. After cooling, the solution was diluted with methanol. The precipitate was filtrated and dissolved in 10 mL of water. After the insoluble impurity was removed by filtration, the product was precipitated from the filtrate by dilution with large amount of ethanol. Yield: 96%. The characterization matches well with the reported data.

^{1}H NMR of SC6A-12C (400 MHz, DMSO-d_6, δ): 7.39 (s, 12H; Ar-H), 4.55 (d, J = 13.8 Hz, 6H; Ar-CH_2-Ar), 3.60 (s, 12H; -CH_2-O-Ar), 1.80 (m, 12H; -CH_2-CH_2-O-Ar), 1.65–1.15 (m, 108H; alkyl-CH_2-), 0.85 (t, J = 6.7 Hz, 18H; alkyl-CH_3) ppm.

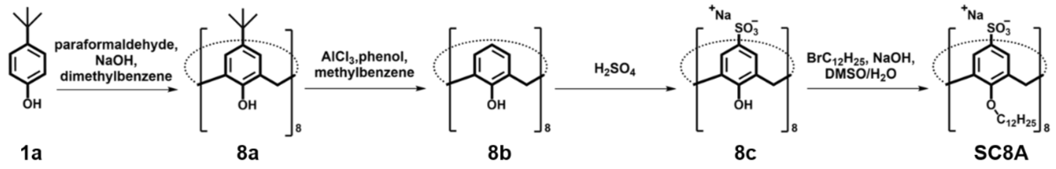

Supplementary Figure 8. The synthetic route of SC8A.

SC8A was synthesized and purified according to the literature procedures.[10–11] **8a**: A slurry prepared from **1a** (100.0 g, 0.7 mol), paraformaldehyde (35 g, 1.1 mol), 2 mL sodium hydroxide (10 M) and 600 mL of xylene was placed in a round-bottomed, three-necked flask. The stirred contents of the flask were heated to reflux by means of a heating mantle under argon for 4 h and a white precipitate formed. After the mixture cooling to room temperature, the precipitate was removed by filtration. The crude product is washed, in succession, with 400 mL portions of toluene, diethyl ether, acetone, and water and was then dried under reduced pressure. It was dissolved in 1600 mL of boiling chloroform. The chloroform was concentrated to 1200 mL. After the mixture cooling to room temperature, the precipitate was collected by filtration to yield a colorless powder. Yield: 62%. **8b**: **8a** (10.0 g, 7.7 mmol), anhydrous aluminium chloride (12.4 g, 93.0 mmol), phenol (5.8 g, 61.6 mmol) and methylbenzene (150 mL) were added to a round bottom flask. The reaction mixture was reacted at room temperature for 3 h under argon. The reaction mixture was quenched by the addition of dilute hydrochloric acid. After stirring for 30 min, the reaction mixture was separated.

The organic phase was concentrated and methanol was added. After suction filtration, the residue was washed with dilute hydrochloric acid, methanol, chloroform, acetone and diethyl ether. The product was white solid. Yield: 93%. **8c**: The synthesis and purification methods refer to the experimental steps of **6b**. The product of white solid was in 88% yield. SC8A: The synthesis and purification methods refer to the experimental steps of SC6A. The product of white solid was in 90% yield. The characterization matches well with the reported data.

^{1}H NMR (400 MHz, DMSO-d_6, δ): 7.27 (s, 16H; Ar-H), 4.30 (d, J = 15.7 Hz, 8H; Ar-CH_2-Ar), 3.70–3.48 (m, 24H; -CH_2-O-Ar and Ar-CH_2-Ar), 1.64 (m, 16H; -CH_2-CH_2-O-Ar), 1.50–1.16 (m, 144H; alkyl-CH_2-), 0.84 (t, J = 6.7 Hz, 24H; alkyl-CH_3) ppm.

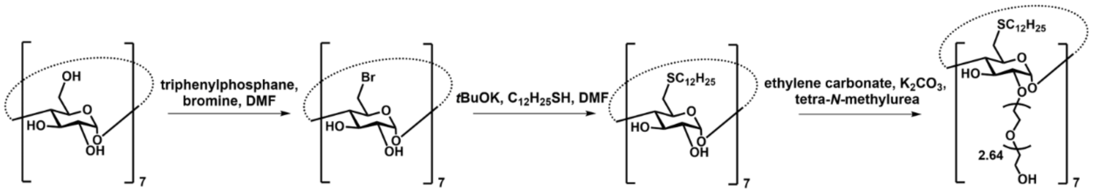

Supplementary Figure 9. The synthetic route of amphiphilic *β*-cyclodextrin.

Amphiphilic β-cyclodextrin (CD) was synthesized and purified according to the literature procedures.[12] To a stirred solution of triphenylphosphane (21 g, 80 mmol) and bromine (4 mL, 80 mmol) in DMF (80 mL) was added **9a** (4.32 g. 26.6 mole equiv.) and the solution was stirred at 80 °C for 15 h. It was then concentrated under vacuum to half the volume and the pH adjusted to 9−10, by the addition of sodium methoxide in methanol (3 M, 30 mL), with simultaneous cooling. The solution was kept at room temperature for 30 min to destroy the formate esters formed in the reaction, after which it was poured into ice water (1.5 L). The precipitate was collected by filtration to yield **9b** (5.57 g, 93%). **9c** was prepared by reaction of **9b** with linear thiols $C_{12}H_{25}SH$ by nucleophilic displacement reactions. Heptakis(6-alkylthio-6-deoxy)-*β*-cyclodextrin (ca. 1 mmol), K_2CO_3 (10% by weight relative to cyclodextrin) and ethylene carbonate (50 molar equiv.) were dissolved in 10 mL of tetra-*N*-methylurea. The mixture was stirred at 150 °C under nitrogen for 4 h, after which time TLC analysis (chloroform/methanol/water = 50:10:1) indicated complete conversion of compound **9c** (R_f = 0) into product (R_f = 0.55), respectively. Furthermore, after this period carbon dioxide evolution had ceased. The solvent was removed at 100 °C under reduced pressure. The crude product was dissolved in the minimum quantity of methanol and was purified by size-exclusion chromatography with methanol as eluent. Product was isolated in 89% yield as a wax. The characterization matches well with the reported data.

^{1}H NMR (300 MHz, $CDCl_3$, δ): 0.89 (t, 21H, CH_3), 1.27 (br s, 126H, CH_2), 1.60 (m, 14H, CH_2), 2.60 (m, 14H, SCH_2), 3.00 (m, 14H, H6), 3.3−4.0 (m, ca. 84H, H2−5 and OCH_2CH_2O), 5.05 (br, 7H, H1) ppm.

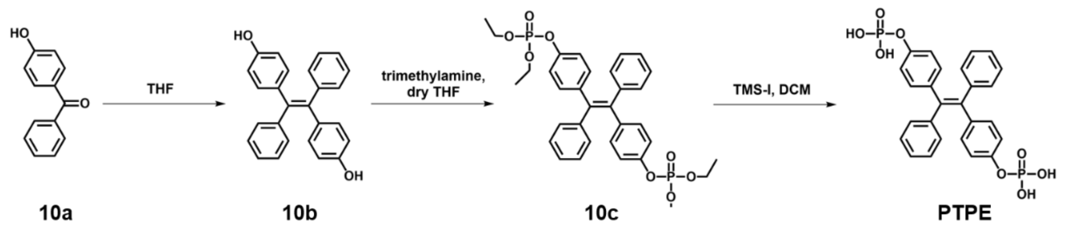

Supplementary Figure 10. The synthetic route of PTPE.
PTPE was synthesized and purified according to the literature procedures.[13] **10b**: A mixture of benzophenone (1.8 g, 10.0 mmol), 4-hydroxybenzophenone (2.0 g, 10.0 mmol) and zinc dust (1.3 g, 20.0 mmol) was dissolved in dry THF and cooled in an ice bath under nitrogen for 30 min, then $TiCl_4$ (2.2 mL, 20 mmol) was added dropwise. After refluxing overnight, the reaction suspension was cooled to room temperature, poured into 100 mL 10% K_2CO_3 aqueous solution and vigorously stirred for 5 min. The final mixture was filtered to remove the insoluble compounds, and extracted with ethyl acetate to get the organic fraction. After drying with Na_2SO_4, the solvent was removed by vacuum rotary evaporation. The product was finally purified by silica gel with petroleum ether (60–90 °C)-ethyl acetate as an eluent. Yield: 25%. **10c** was synthesized from **10b** (1.1 g, 3.0 mmol), trimethylamine (1.3 mL, 9.0 mmol) and chlorophosphoric acid diethyl ester (0.7 mL, 4.5 mmol) in 8 mL dry THF, and the reaction mixture was stirred at room temperature under nitrogen overnight. Then the reaction was quenched with 1 mL cool water, and the organic fraction was extracted and dried with Na_2SO_4. The solvent was removed by vacuum rotary evaporation and purified by silica gel with petroleum ether (60–90 °C)-ethyl acetate as an eluent. Yield: 55%. **PTPE**: **10c** (0.3 g, 0.5 mmol) was dissolved in 5 mL dry DCM, then TMS-I (0.4 mL, 3.0 mmol) was added dropwise using a syringe. The reaction system was tightly sealed and further reacted for 4 h at room temperature. Then the solvent was removed by vacuum rotary evaporation and the residue was purified by washing with DCM several times. The product was further purified by dissolving in deionized water and lyophilized. Yield: 70%. The characterization matches well with the reported data.
^{1}H NMR (400 MHz, DMSO-d_6, δ): 7.20–7.08 (m, 6H), 7.00–6.85 (m, 12H) ppm.

Supplementary Note 2. Characterizations of compounds.

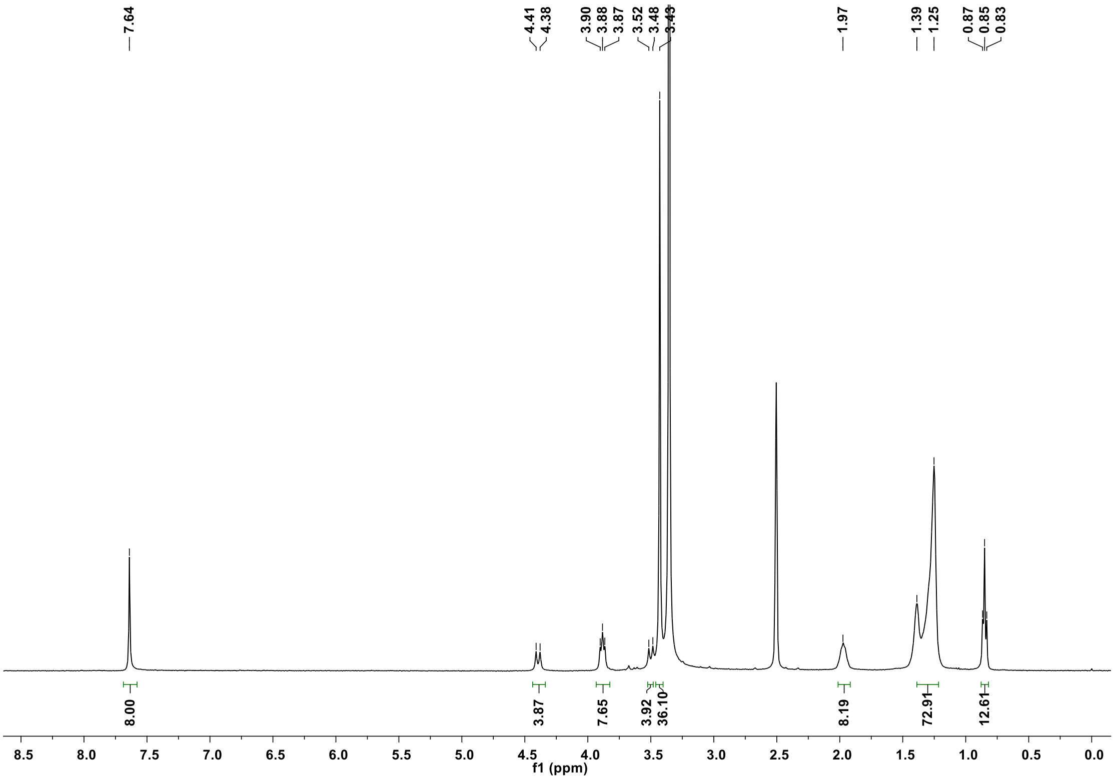

Supplementary Figure 11. ^{1}H NMR spectrum of QC4A in DMSO-*d*$_6$, 400 MHz, 25 °C.

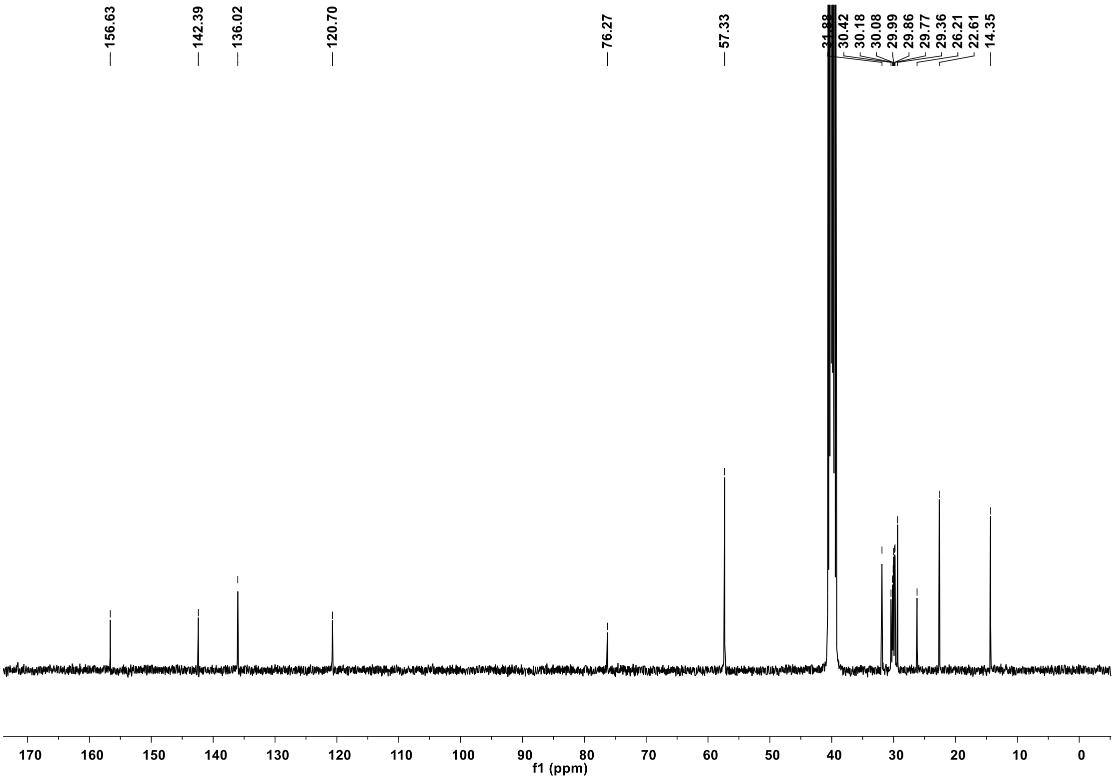

Supplementary Figure 12. ^{13}C NMR spectrum of QC4A in DMSO-*d*$_6$, 100 MHz, 25 °C.

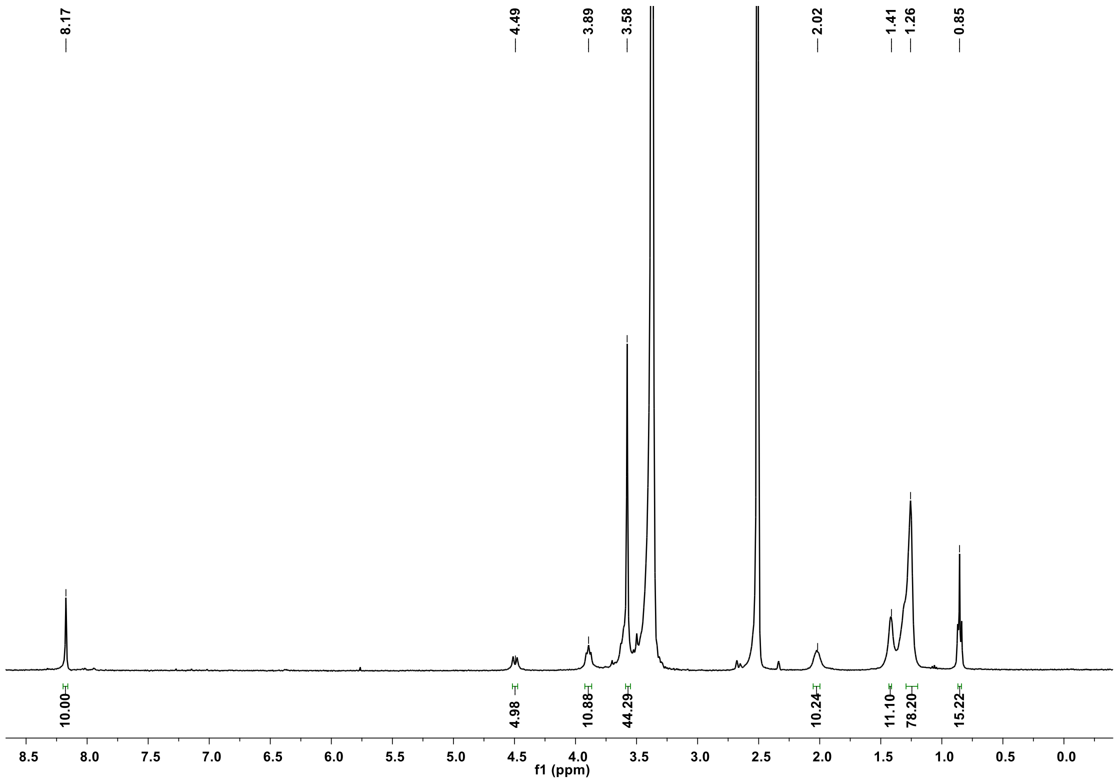

Supplementary Figure 13. ^{1}H NMR spectrum of QC5A in DMSO-*d*$_6$, 400 MHz, 25 °C.

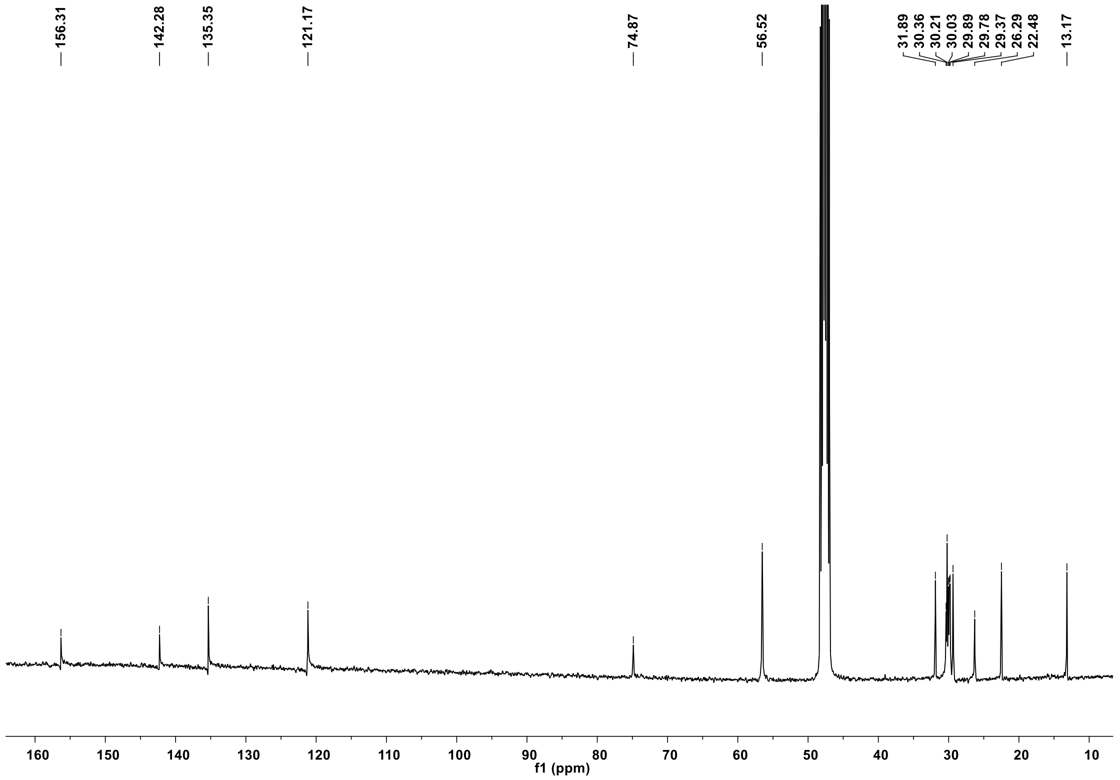

Supplementary Figure 14. ^{13}C NMR spectrum of QC5A in DMSO-*d*$_6$, 100 MHz, 25 °C.

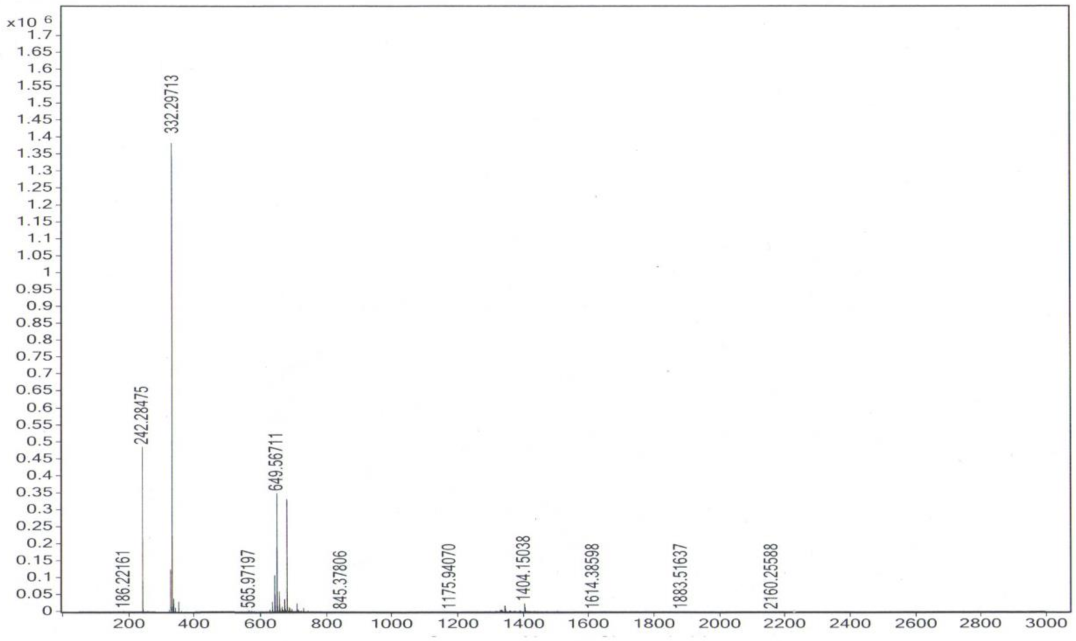

Supplementary Figure 15. Mass spectrum (ESI) of QC4A.

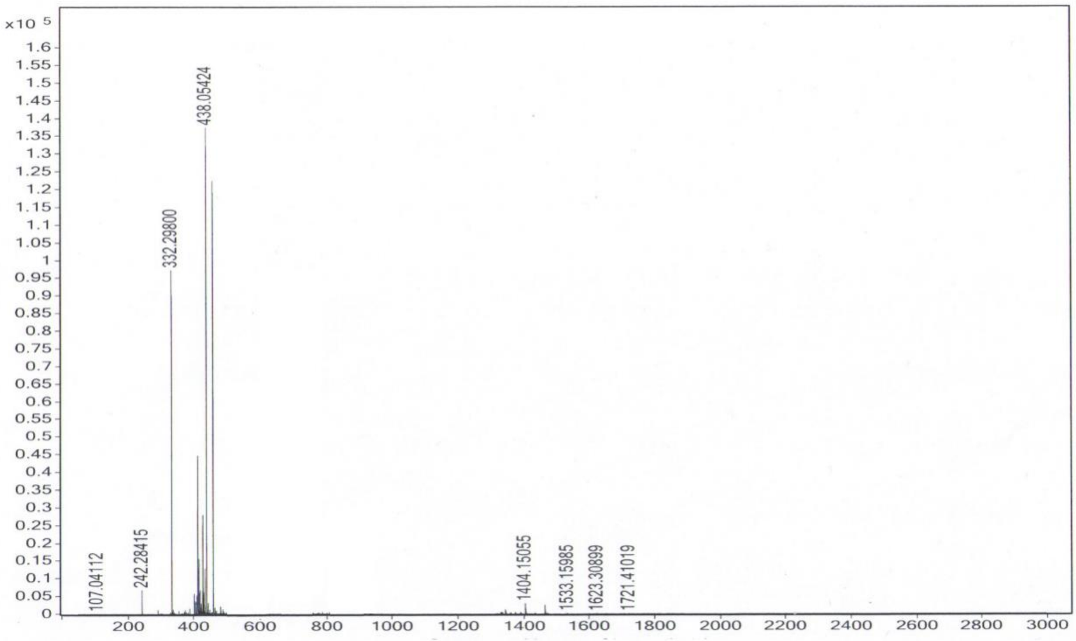

Supplementary Figure 16. Mass spectrum (ESI) of QC5A.

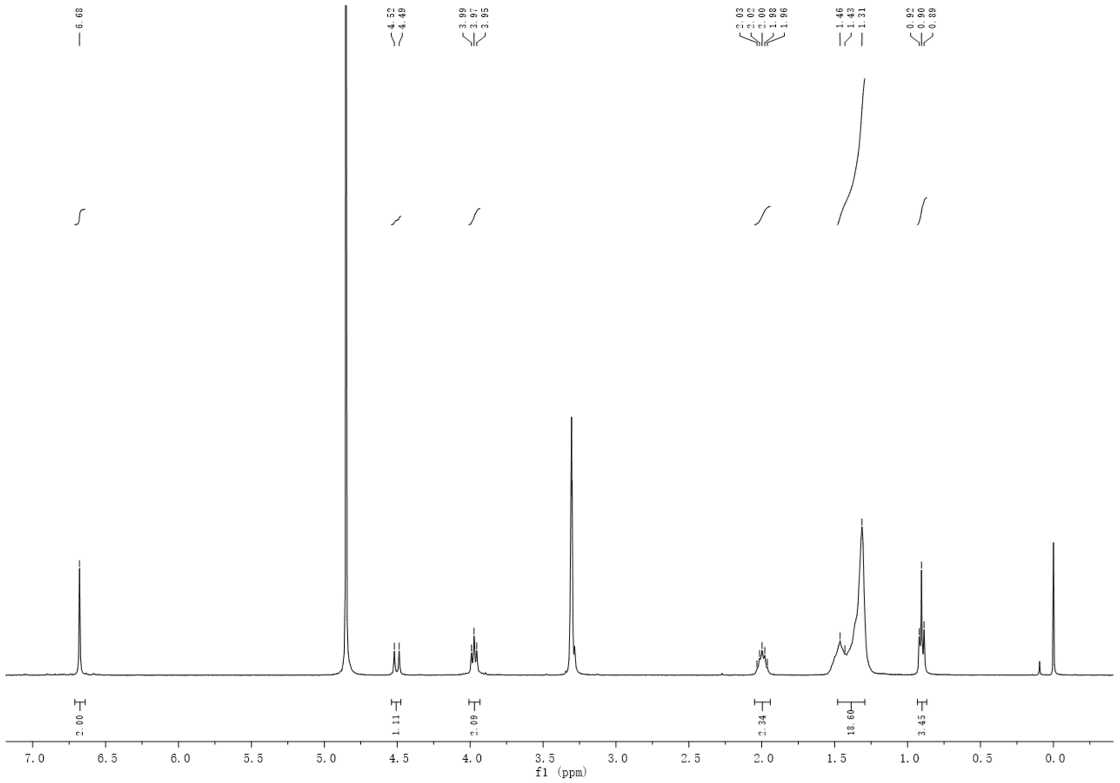

Supplementary Figure 17. ^{1}H NMR spectrum of GC4A in CD_3OD, 400 MHz, 25 °C.

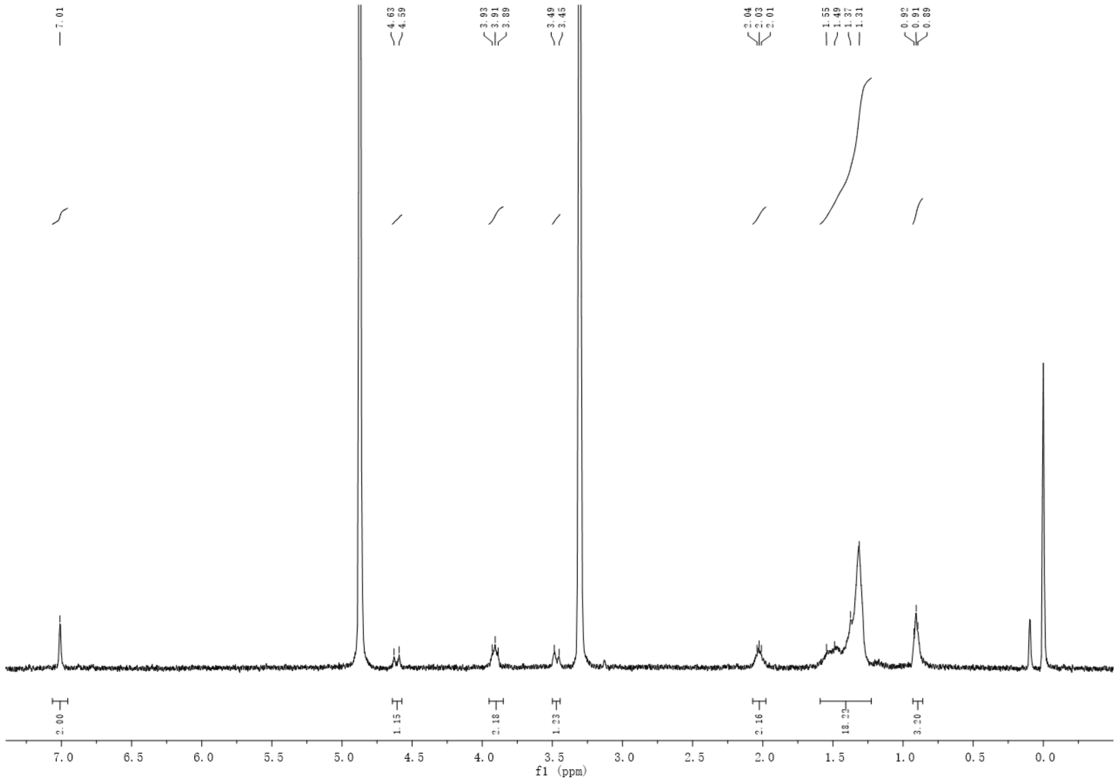

Supplementary Figure 18. ^{1}H NMR spectrum of GC5A in CD_3OD, 400 MHz, 25 °C.

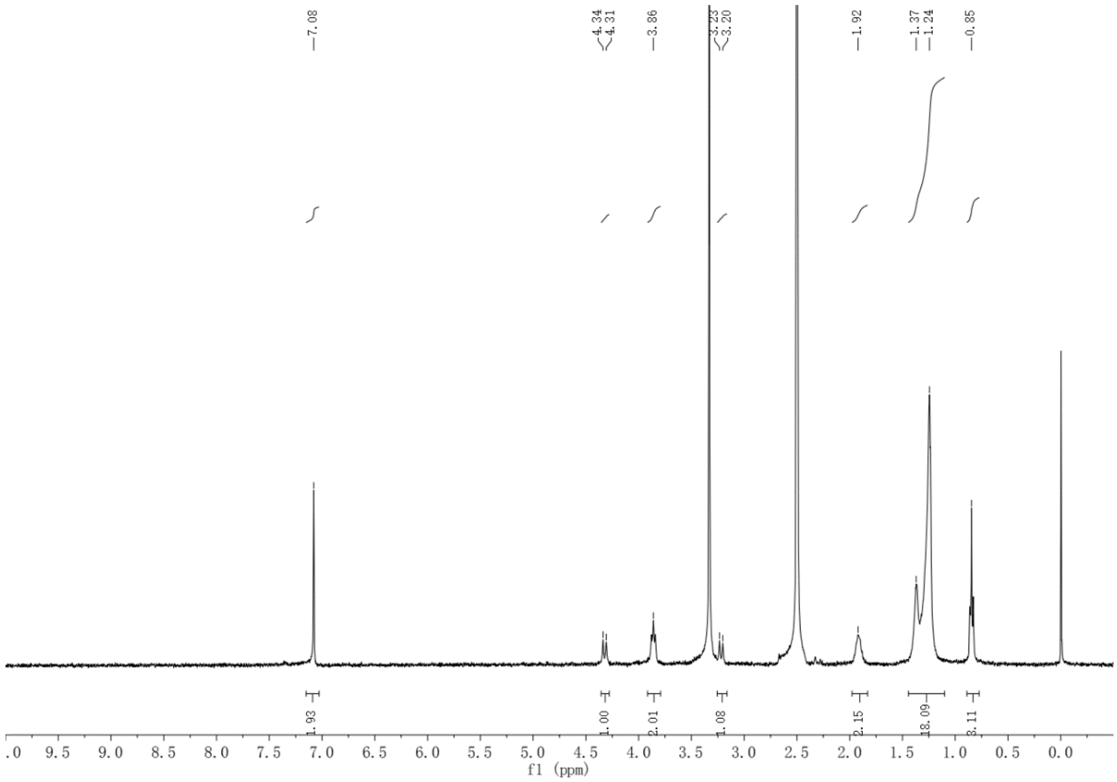

Supplementary Figure 19. ^{1}**H NMR spectrum of SC4A in DMSO-**d_6**, 400 MHz, 25 °C.**

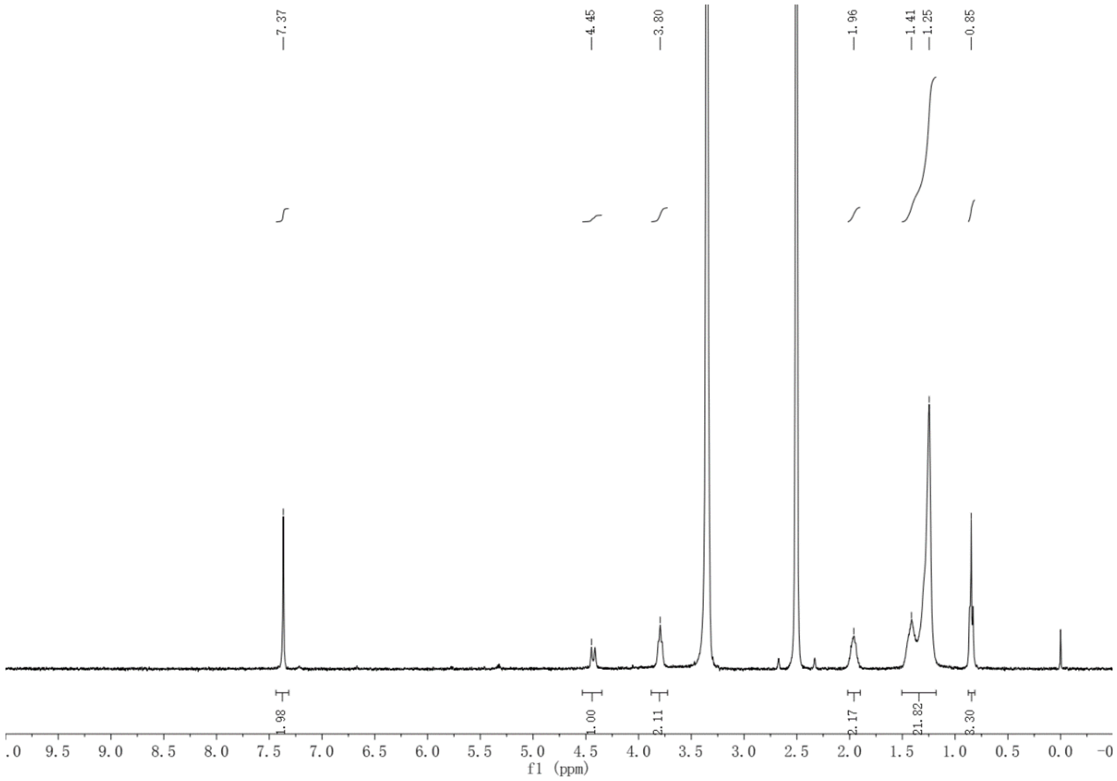

Supplementary Figure 20. ^{1}**H NMR spectrum of SC5A in DMSO-**d_6**, 400 MHz, 25 °C.**

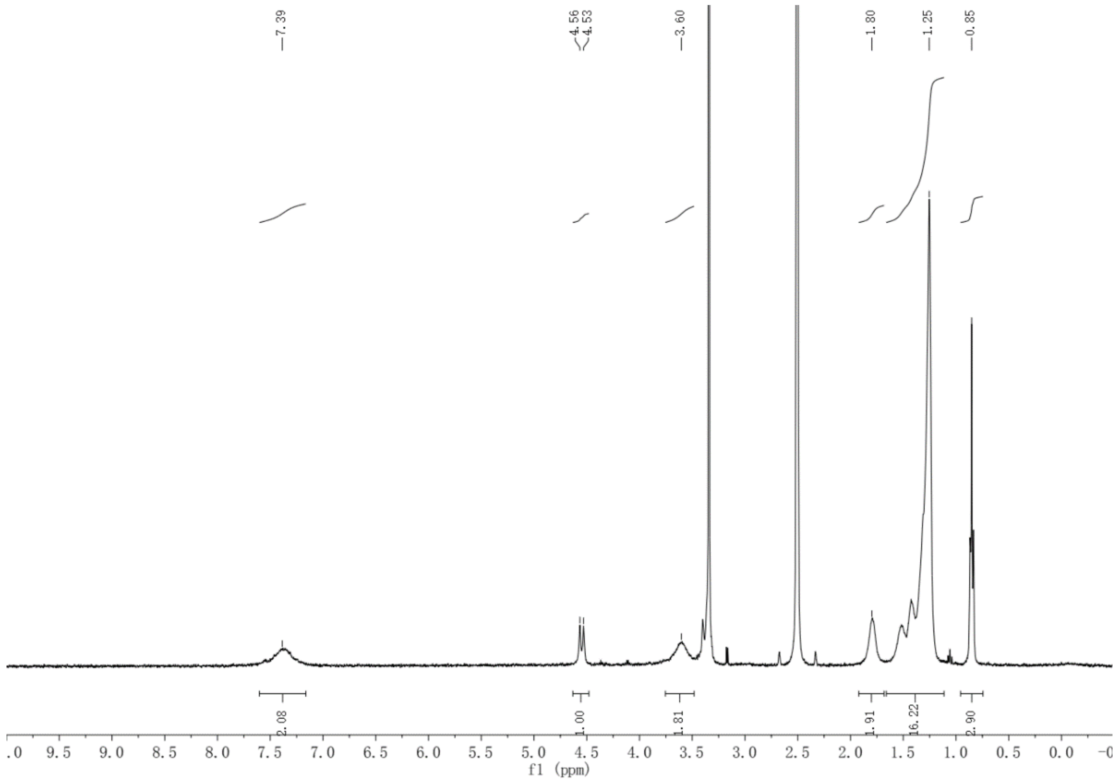

Supplementary Figure 21. **^{1}H NMR spectrum of SC6A in DMSO-d_6, 400 MHz, 25 °C.**

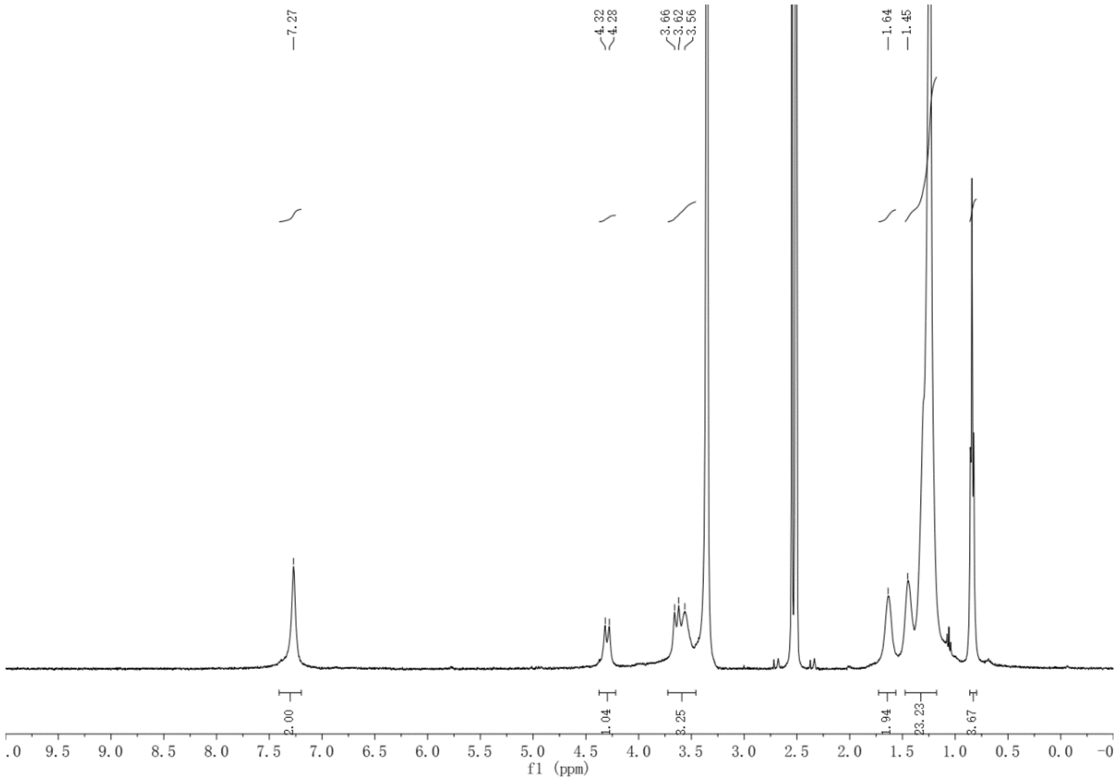

Supplementary Figure 22. **^{1}H NMR spectrum of SC8A in DMSO-d_6, 400 MHz, 25 °C.**

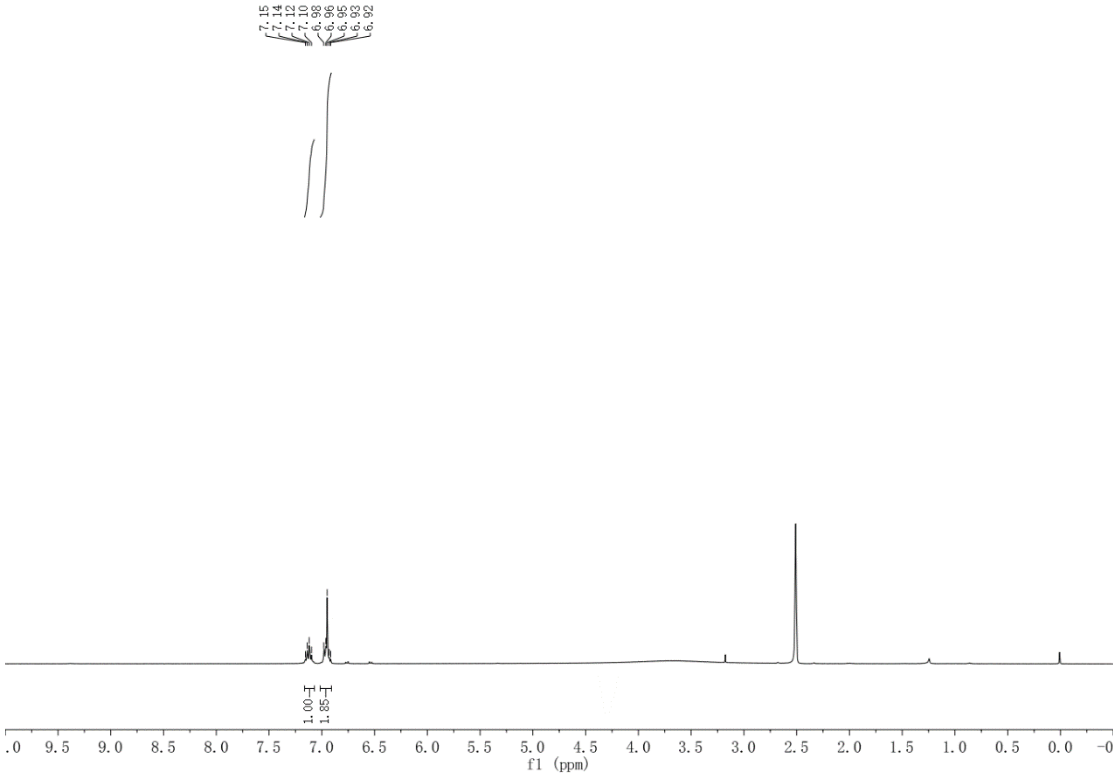

Supplementary Figure 23. ^{1}H NMR spectrum of PTPE in DMSO-d_6, 400 MHz, 25 °C.

Supplementary Note 3. Basic properties of the target proteins.

Supplementary Table 1. Basic properties of the target proteins.

Protein	Molecular weight (kDa)	Isoelectric point
Hb	64.5	7.1
TRF	77.0	5.6
CytC	12.3	10.7
Lys	14.4	11
Mb	17.8	7.6
Lip	58.0	5.6
Try	23.3	10.5
HSA	69.4	5.2
BSA	66.4	4.8
BHb	64.5	6.7
Ova	44.5	4.6
Pap	23.4	8.8
Sal	6~7	12.1

Supplementary Table 2. The effect of proteins ([protein] = 23.8 μg/mL) on the pH of water. The pH of water used in this work was 6.53.

Protein	pH
Hb	6.41
TRF	6.45
CytC	6.46
Lys	6.50
Mb	6.50
Lip	6.31
Try	6.43
HSA	6.63
BSA	6.59
BHb	6.28
Ova	6.32
Pap	6.26
Sal	6.37

Supplementary Note 4. Preparation and Characterization of the CA-CD coassemblies.

Preparation of assemblies. To prepare the GC*n*A-CD and QC*n*A-CD coassemblies, CA and CD were dissolved in methanol, respectively, at a concentration of 1 mM. The mixed organic solution of CA and CD in the ratio of 1:1 (or different ratios) was dried under high vacuum for 4 h to yield a thin film in a glass vial. Pure water was added and the solution was sonicated at 80 °C for 3 h to make the CA-CD coassemblies. To prepare the SC*n*A-CD coassemblies, firstly, the methanol solution of CD was dried under high vacuum for 4 h to yield a thin film in a glass vial. Then the aqueous solution of equal concentration of SC*n*As was added and the solution was sonicated at 80 °C for 3 h. The coassemblies were characterized using DLS and zeta potential.

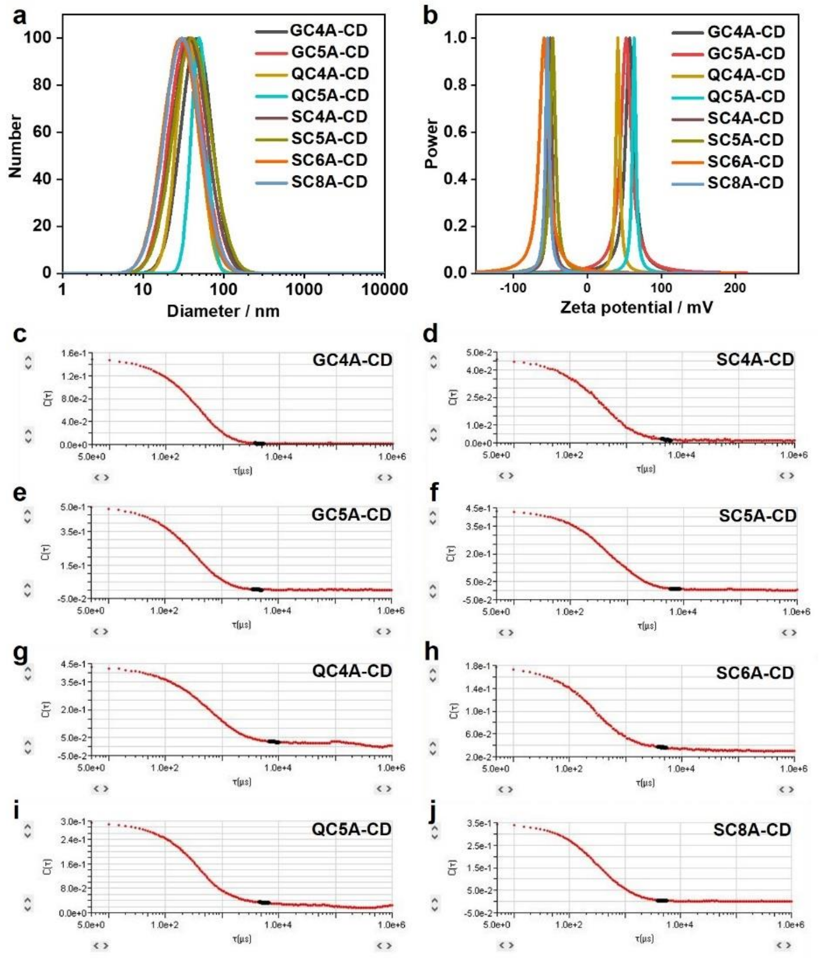

Supplementary Figure 24. DLS and zeta potential data. (a) DLS data and (b) zeta potentials of the CA-CD coassemblies in water. ([CA] = [CD] = 20.0 μM). The correlation functions for the DLS measurements of (c) GC4A-CD, (d) SC4A-CD, (e) GC5A-CD, (f) SC5A-CD, (g) QC4A-CD, (h) SC6A-CD, (i) QC5A-CD and (j) SC8A-CD.

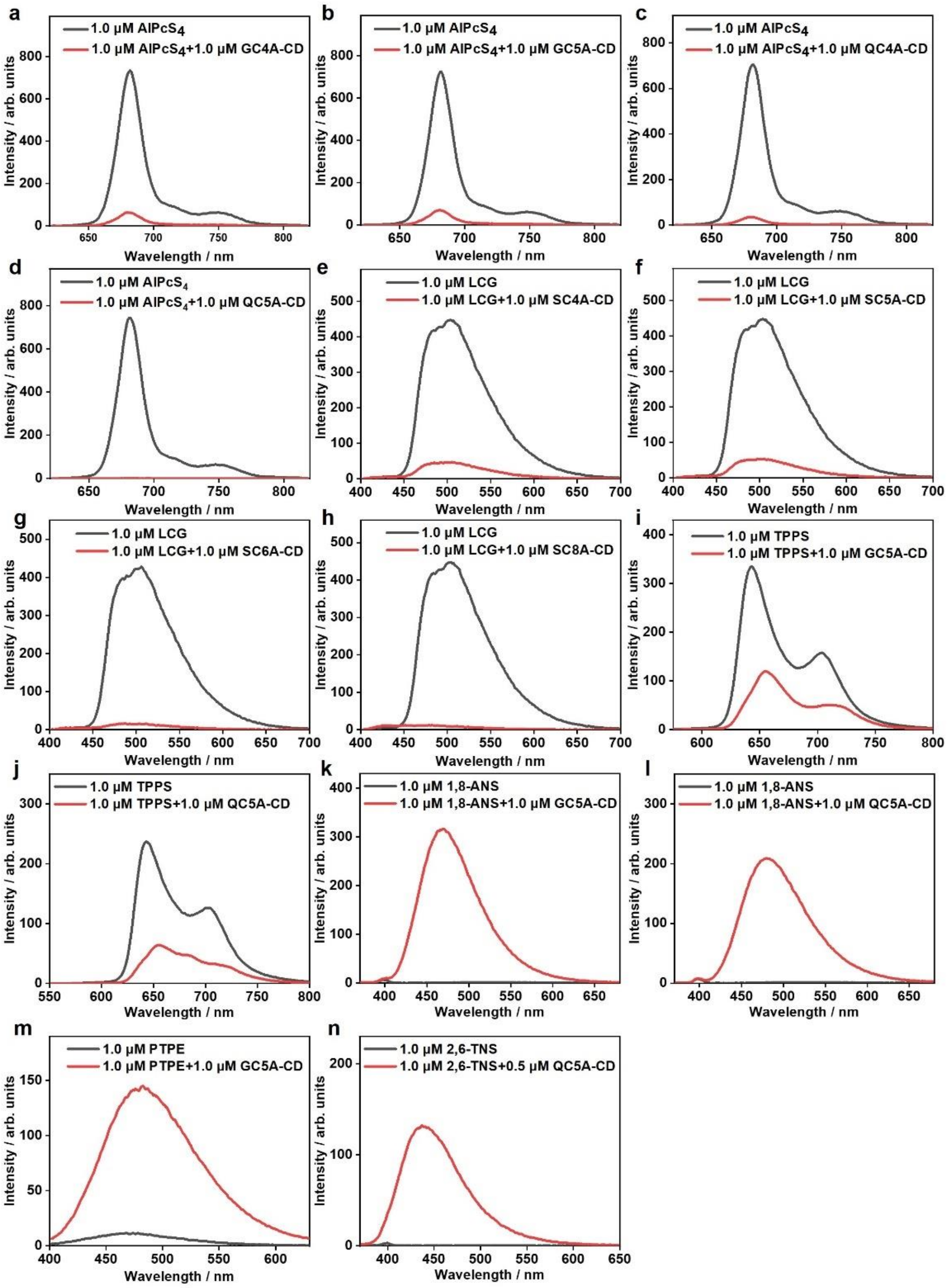

Supplementary Figure 25. Fluorescence spectra of dyes in the absence and presence of coassemblies. Fluorescence quenching or enhancement of (a–d) $AlPcS_4$ (λ_{ex} = 610 nm), (e–h) LCG (λ_{ex} = 365 nm), (i, j) TPPS (λ_{ex} = 412 nm), (k, l) 1,8-ANS (λ_{ex} = 350 nm), (m) PTPE (λ_{ex} = 327 nm) and (n) 2,6-TNS (λ_{ex} = 350 nm) by the host-guest complexation of different CA-CD coassemblies ([CA] = [CD] = 1.0 μM, [dye] = 1.0 μM).

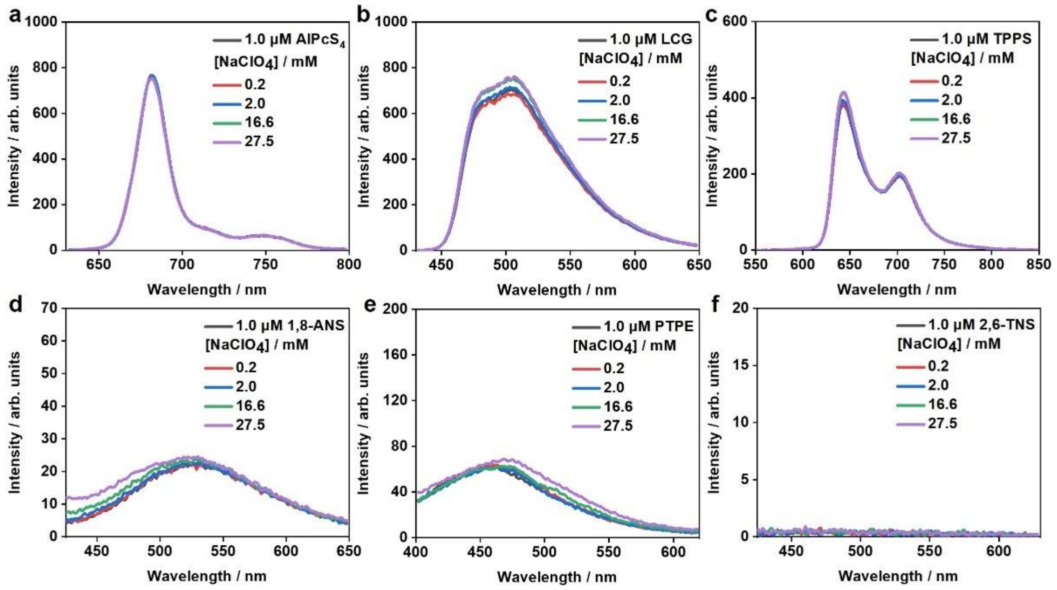

Supplementary Figure 26. Fluorescence spectra of dyes in the absence and presence of $NaClO_4$. Fluorescence spectra of (a) $AlPcS_4$ (λ_{ex} = 610 nm), (b) LCG (λ_{ex} = 365 nm), (c) TPPS (λ_{ex} = 412 nm), (d) 1,8-ANS (λ_{ex} = 350 nm), (e) PTPE (λ_{ex} = 327 nm) and (f) 2,6-TNS (λ_{ex} = 350 nm) in the presence of $NaClO_4$ from 0.2 to 27.5 mM.

Supplementary Note 5. Theoretical modeling and supporting results.

Model reporter pairs were given with assumed binding constants. The free dye concentrations can be calculated using the following formulas. We considered that a dye (D) formed a 1:1 host-dye complex with a host (H) at an association constant (K_a), which satisfied the respective law of mass action relating to the equilibrium concentrations of free host, [H], free dye, [D], and host-dye complex, [HD]. Also, the relationship between the total concentrations of host, $[H]_0$, and dye, $[D]_0$, and their equilibrium concentrations were introduced by the law of mass conservation (equations 2−1 and 2−2). Among them, K_a, $[H]_0$ and $[D]_0$ are all given values. [D] can be obtained by calculation. We considered that the free dye concentration at this time represented the initial fluorescence intensity (I_0). Here, [D] was calculated using a JavaScript-based tool from the website of Prof. Nau's group (http://www.jacobs-university.de/ses/wnau) under the column of "Data Analysis".

$$[H]+[D] \underset{}{\overset{K_a}{\rightleftharpoons}} [HD] \qquad K_a = \frac{[HD]}{[H][D]} \tag{1}$$

$$[D] = [D]_0 - [HD] \tag{2−1}$$

$$[H] = [H]_0 - [HD] \tag{2−2}$$

$$[HD] = \frac{K_a[H][D]_0}{1+K_a[H]} \tag{3}$$

The binding constants between hosts and analytes were also assumed. When the analytes were added to the host-dye complex, part of the dye was replaced by competition. The total concentrations of free dye were calculated by the following formulas. The competitor (C) could bind to a host's cavity in a 1:1 stoichiometry at an association constant (K_C). Free host, [H], free analyte, [C], and host-analyte complex, [HC] obeyed the respective law of mass action referring to the equilibrium concentrations. Also, $[H]_0$ and the total concentrations of analyte, $[C]_0$, and their equilibrium concentrations satisfied the law of mass conservation (equations 5−1 and 5−2). Among them, K_a, K_C, $[H]_0$, $[D]_0$ and $[C]_0$ were all given values. [H] can be obtained by solving a cubic equation in one variable (equation 6). Then according to equations 1 and 2−1 together, the changed [D] can be obtained by calculation. We considered that the concentration of free dye at this time represented the final fluorescence intensity (I).

$$[H]+[C] \overset{K_C}{\rightleftharpoons} [HC] \qquad K_C = \frac{[HC]}{[H][C]} \tag{4}$$

$$[H] = [H]_0 - [HD] - [HC] \tag{5−1}$$

$$[C] = [C]_0 - [HC] \tag{5−2}$$

$$
\begin{aligned}
&0 = \mathrm{A}[\mathrm{H}]^3 + \mathrm{B}[\mathrm{H}]^2 + \mathrm{C}[\mathrm{H}] + \mathrm{D}, \text{ where} \\
&\mathrm{A} = K_\mathrm{a}K_\mathrm{C} \\
&\mathrm{B} = K_\mathrm{a} + K_\mathrm{C} + K_\mathrm{a}K_\mathrm{C}([\mathrm{D}]_0 + [\mathrm{C}]_0 - [\mathrm{H}]_0) \\
&\mathrm{C} = K_\mathrm{C}([\mathrm{C}]_0 - [\mathrm{H}]_0) - K_\mathrm{a}([\mathrm{H}]_0 - [\mathrm{D}]_0) + 1 \\
&\mathrm{D} = -[\mathrm{H}]_0
\end{aligned}
\tag{6}
$$

The ratio of the final fluorescence intensity to the initial fluorescence intensity (I/I_0) was considered to be the fluorescence response signal of the sensor unit caused by the analyte. Subsequently, six random numbers around the calculated I/I_0 value as repeated data were given by the random number generator in Microsoft Excel software. Each group of random numbers conformed to a normal distribution with a mean value of I/I_0 and a standard deviation of 0.1. The obtained matrix was used for LDA analysis to obtain a two-dimensional score plot of the model.

Supplementary Table 3. The fluorescence response values calculated according to the assumed binding constants using a simulated sensor array based on different reporter pairs.

Assumed binding constant between receptor and dye / M^{-1}		Sensor unit concentration / M	Calculated free dye concentration (I_0) / M	Assumed binding constant between receptor and analyte / M^{-1}		Analyte concentration / M	Calculated free dye concentration (I) / M	I/I_0
receptor 1 dye 1 (sensor unit 1)	5.0×10^{6}	1.0×10^{-6}	3.6×10^{-7}	receptor 1 analyte 1	8.0×10^{6}	1.0×10^{-6}	6.2×10^{-7}	1.7
				receptor 1 analyte 2	4.0×10^{5}		4.0×10^{-7}	1.1
				receptor 1 analyte 3	2.0×10^{3}		3.6×10^{-7}	1.0
receptor 2 dye 2 (sensor unit 2)	5.0×10^{7}	1.0×10^{-6}	1.3×10^{-7}	receptor 2 analyte 1	5.0×10^{4}		1.4×10^{-7}	1.0
				receptor 2 analyte 2	7.0×10^{5}		1.7×10^{-7}	1.3
				receptor 2 analyte 3	3.0×10^{5}		1.5×10^{-7}	1.1
receptor 3 dye 3 (sensor unit 3)	5.0×10^{8}	1.0×10^{-6}	4.0×10^{-8}	receptor 3 analyte 1	9.0×10^{7}		3.0×10^{-7}	7.5
				receptor 3 analyte 2	5.0×10^{4}		4.5×10^{-8}	1.1
				receptor 3 analyte 3	4.0×10^{6}		9.2×10^{-8}	2.3

Supplementary Table 4. Six random numbers generated from the calculated fluorescence response values from Supplementary Table 3, used to construct the data matrix for LDA analysis.

	sensor unit 1	sensor unit 2	sensor unit 3
analyte 1	1.922437	1.150879	7.545922
analyte 1	1.801074	0.905300	7.501182
analyte 1	1.920569	1.181741	7.456075
analyte 1	1.697693	1.066051	7.480488
analyte 1	1.691748	0.970792	7.487857
analyte 1	1.756658	1.035610	7.631927
analyte 2	0.871961	1.128976	0.975619
analyte 2	1.065147	1.397936	1.135195
analyte 2	1.182220	1.182988	1.139849
analyte 2	1.105967	1.288473	1.050097
analyte 2	1.051470	1.411275	0.984923
analyte 2	1.070257	1.486185	0.998274
analyte 3	1.120433	1.253834	2.427665
analyte 3	0.922629	0.966839	2.228719
analyte 3	0.865698	1.147107	2.243495
analyte 3	1.038387	1.213738	2.305928
analyte 3	1.026837	1.121731	2.284567
analyte 3	1.123458	1.120565	2.537824

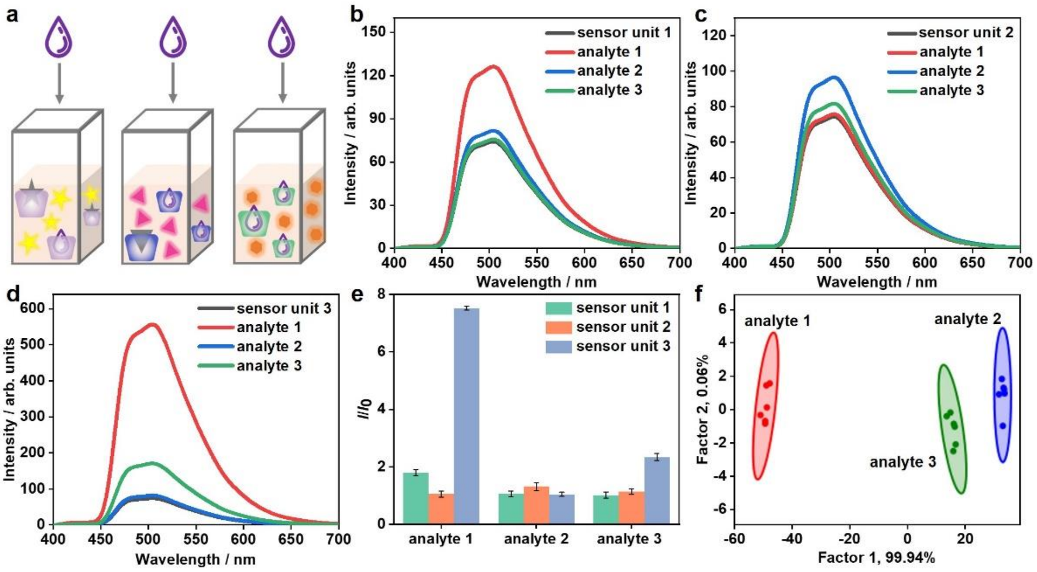

Supplementary Figure 27. Theoretically model of sensor array based on different reporter pairs. (a) Schematic diagram of the sensor array formed by different reporter pairs. (b–d) Theoretically simulated fluorescence spectra produced by sensor units after adding analytes. (e) Fluorescence response patterns of the simulated sensor array. (f) Canonical score plot for the two factors of simplified fluorescence response patterns obtained from LDA with 95% confidence ellipses based on the simulated data. (Because several repetitions are needed for each analyte to allow the use of statistical methodologies to reduce the values into simpler, smaller vector, here we generated six values distributed randomly around the calculated fluorescence response value in order to obtain this simulated discrimination.) Error bars in (e) represent mean ± s.d. ($n = 6$ random numbers).

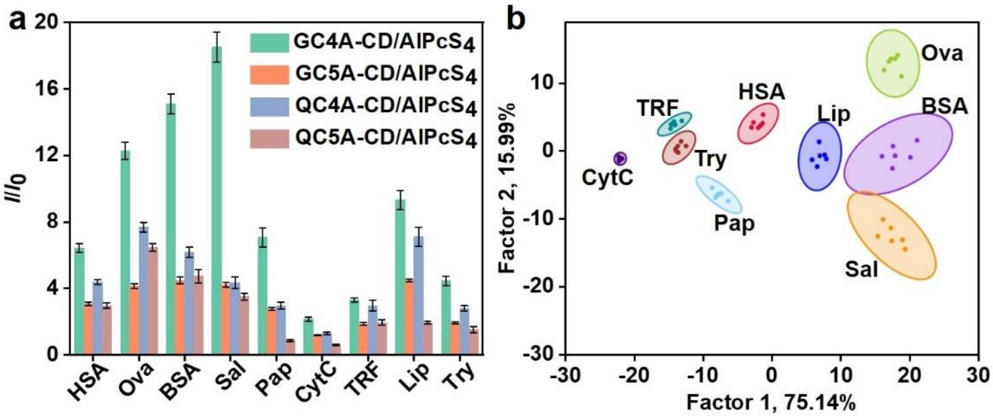

Supplementary Figure 28. Pattern recognition of proteins using SA2 ([CA] = [CD] = 1.0 μM, [AlPcS$_4$] = 1.0 μM). (a) Fluorescence response patterns of **SA2** against various proteins (23.8 μg/mL for each protein). (b) Canonical score plot for the two factors of simplified fluorescence response patterns obtained from LDA with 95% confidence ellipses. Error bars in (a) represent mean ± s.d. ($n = 6$ independent experiments).

Supplementary Table 5. The fluorescence response values calculated according to the assumed binding constants using a simulated sensor array based on dye replacement.

Assumed binding constant between receptor and dye / M^{-1}		Sensor unit concentration / M	Calculated free dye concentration (I_0) / M	Assumed binding constant between receptor and analyte / M^{-1}		Analyte concentration / M	Calculated free dye concentration (I) / M	I/I_0
receptor 1 dye 1 (sensor unit 1)	5.0×10^{6}	1.0×10^{-6}	3.6×10^{-7}	receptor 1 analyte 1	8.0×10^{6}	1.0×10^{-6}	6.2×10^{-7}	1.7
				receptor 1 analyte 2	2.0×10^{3}		3.6×10^{-7}	1.0
				receptor 1 analyte 3	9.0×10^{7}		8.3×10^{-7}	2.3
receptor 1 dye 2 (sensor unit 2)	5.0×10^{7}	1.0×10^{-6}	1.3×10^{-7}	receptor 1 analyte 1	8.0×10^{6}		3.1×10^{-7}	2.4
				receptor 1 analyte 2	2.0×10^{3}		1.3×10^{-7}	1.0
				receptor 1 analyte 3	9.0×10^{7}		5.8×10^{-7}	4.5
receptor 1 dye 3 (sensor unit 3)	5.0×10^{8}	1.0×10^{-6}	4.0×10^{-8}	receptor 1 analyte 1	8.0×10^{6}		1.2×10^{-7}	3.0
				receptor 1 analyte 2	2.0×10^{3}		4.4×10^{-8}	1.1
				receptor 1 analyte 3	9.0×10^{7}		3.0×10^{-7}	7.5

Supplementary Table 6. Six random numbers generated from the calculated fluorescence response values from Supplementary Table 5, used to construct the data matrix for LDA analysis.

	sensor unit 1	sensor unit 2	sensor unit 3
analyte 1	1.580697	2.632028	2.930493
analyte 1	1.687502	2.531472	3.264459
analyte 1	1.647752	2.349793	3.041303
analyte 1	1.781632	2.361785	3.125131
analyte 1	1.822044	2.515300	2.818139
analyte 1	1.640380	2.432586	2.770173
analyte 2	0.900307	0.942433	1.030493
analyte 2	1.183648	0.987402	1.284565
analyte 2	0.930230	0.802241	1.064430
analyte 2	1.054426	1.088565	1.204789
analyte 2	1.077402	0.914804	1.169137
analyte 2	1.035097	0.987926	1.026973
analyte 3	2.355137	4.672563	7.429320
analyte 3	2.326916	4.460606	7.545837
analyte 3	2.443642	4.527226	7.635979
analyte 3	2.479627	4.377891	7.457829
analyte 3	2.247401	4.510203	7.603174
analyte 3	2.183094	4.510065	7.640146

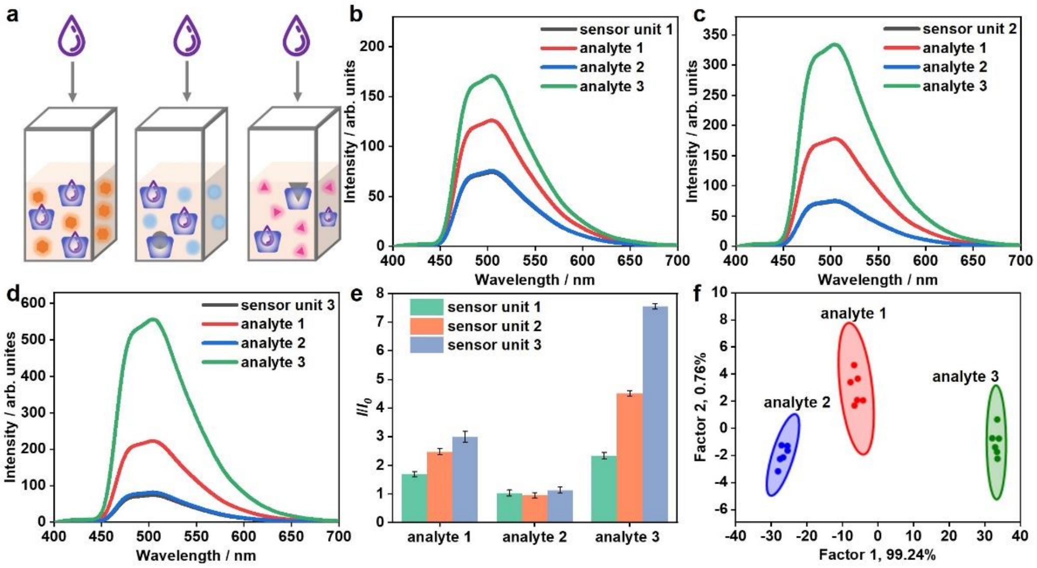

Supplementary Figure 29. Theoretically model of sensor array based on dye replacement. (a) Schematic diagram of the theoretical model of a sensor array formed by dye replacement. (b–d) Theoretical simulated fluorescence spectra produced by sensor units after adding analytes. (e) Fluorescence response patterns of the simulated sensor array. (f) Canonical score plot for the two factors of simplified fluorescence response patterns obtained from LDA with 95% confidence ellipses based on the simulated data. Error bars in (e) represent mean ± s.d. ($n = 6$ random numbers).

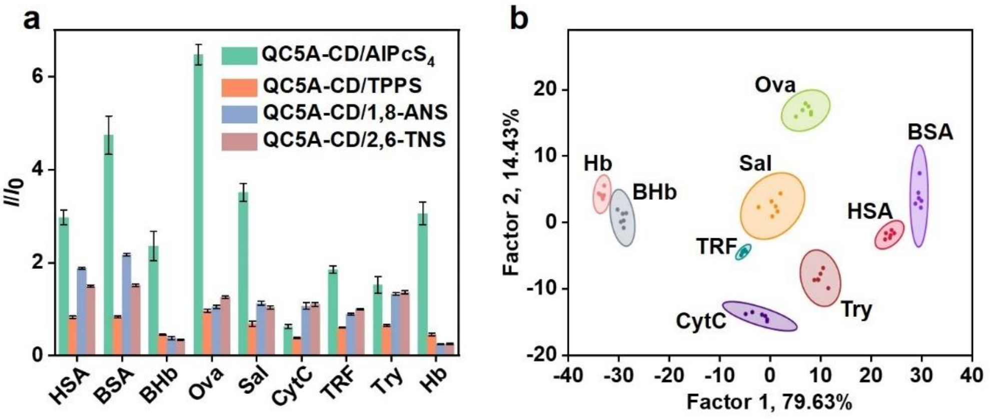

Supplementary Figure 30. Pattern recognition of proteins using SA4 ([QC5A] = [CD] = 1.0 μM, [dye] = 1.0 μM). (a) Fluorescence response patterns of **SA4** against various proteins (23.8 μg/mL for each protein). (b) Canonical score plot for the two factors of simplified fluorescence response patterns obtained from LDA with 95% confidence ellipses. Error bars in (a) represent mean ± s.d. ($n = 6$ independent experiments).

Supplementary Table 7. The fluorescence response values calculated according to the assumed binding constants using a simulated sensor array based on adjusting the coassembly ratio. The concentration of the first macrocycle was fixed at 1.0×10^{-6} M. The concentrations of the second macrocycle were 0.5×10^{-7} M, 1.0×10^{-6} M and 3.0×10^{-6} M. The dye concentrations were consistent with the changed component.

Assumed binding constant between coassembly and dye / M^{-1}		Sensor unit concentration / M	Calculated free dye concentration (I_0) / M	Assumed binding constant between coassembly and analyte / M^{-1}		Analyte concentration / M	Calculated free dye concentration (I) / M	I/I_0
coassembly 1 dye 1 (sensor unit 1)	5.0×10^{7}	5.0×10^{-7}	9.0×10^{-8}	coassembly 1 analyte 1	8.0×10^{6}	1.0×10^{-6}	2.1×10^{-7}	2.3
				coassembly 1 analyte 2	2.0×10^{3}		9.1×10^{-8}	1.0
				coassembly 1 analyte 3	9.0×10^{7}		3.8×10^{-7}	4.2
coassembly 2 dye 1 (sensor unit 2)	5.0×10^{7}	1.0×10^{-6}	1.3×10^{-7}	coassembly 2 analyte 1	9.0×10^{6}		3.2×10^{-7}	2.5
				coassembly 2 analyte 2	3.0×10^{3}		1.3×10^{-7}	1.0
				coassembly 2 analyte 3	1.0×10^{8}		5.9×10^{-7}	4.6
coassembly 3 dye 1 (sensor unit 3)	5.0×10^{7}	3.0×10^{-6}	2.4×10^{-7}	coassembly 3 analyte 1	1.0×10^{7}		5.6×10^{-7}	2.3
				coassembly 3 analyte 2	4.0×10^{3}		2.4×10^{-7}	1.0
				coassembly 3 analyte 3	1.1×10^{8}		8.9×10^{-7}	3.7

Supplementary Table 8. Six random numbers generated from the calculated fluorescence response values from Supplementary Table 7, used to construct the data matrix for LDA analysis.

	sensor unit 1	sensor unit 2	sensor unit 3
analyte 1	2.245494	2.670009	2.493013
analyte 1	2.167228	2.496729	2.347492
analyte 1	2.372240	2.522672	2.445756
analyte 1	2.305139	2.333884	2.366480
analyte 1	2.293566	2.446158	2.175236
analyte 1	2.393060	2.478864	2.334935
analyte 2	0.887682	0.993934	1.072310
analyte 2	1.112794	1.003646	1.083200
analyte 2	0.936188	0.955805	0.936656
analyte 2	0.979388	0.870482	1.065747
analyte 2	1.189766	1.035472	0.991280
analyte 2	1.052213	1.006503	1.129607
analyte 3	4.192615	4.619831	3.715526
analyte 3	4.316997	4.574256	3.733936
analyte 3	4.293426	4.570712	3.577746
analyte 3	4.184412	4.771422	3.536938
analyte 3	4.304829	4.610496	3.635501
analyte 3	4.206357	4.652995	3.721120

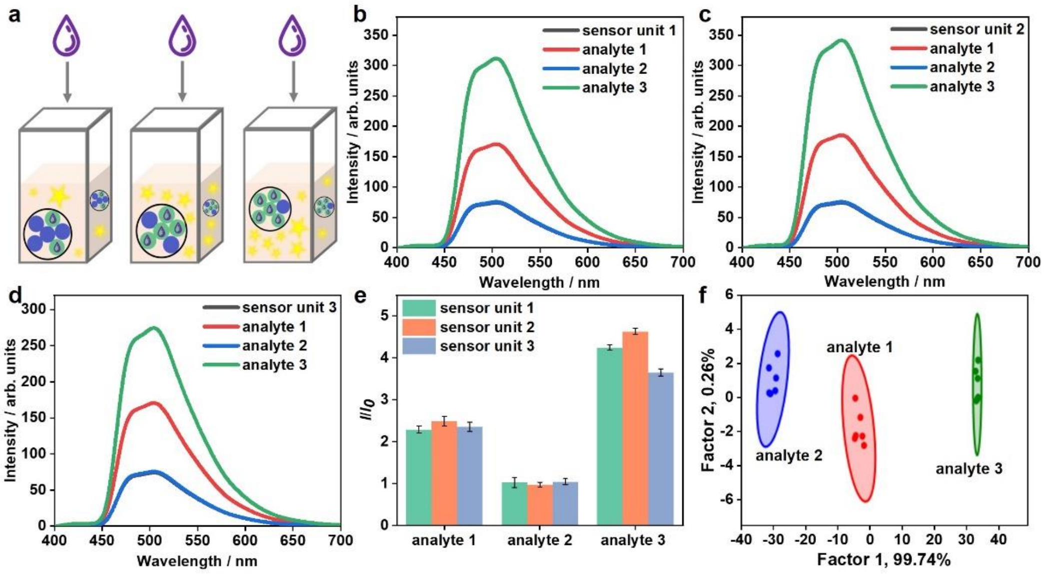

Supplementary Figure 31. Theoretically model of sensor array based on adjusting coassembly ratio. (a) Schematic diagram of the theoretical model of a sensor array formed by adjusting the coassembly ratios. (b–d) Theoretical simulated fluorescence spectra produced by sensor units after adding analytes. (e) Fluorescence response patterns of the simulated sensor array. (f) Canonical score plot for the two factors of simplified fluorescence response patterns obtained from LDA with 95% confidence ellipses based on the simulated data. Error bars in (e) represent mean ± s.d. ($n = 6$ random numbers).

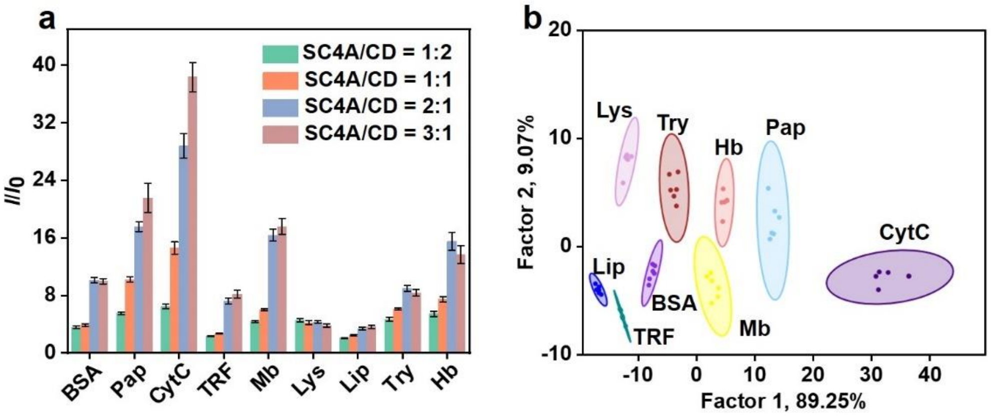

Supplementary Figure 32. Pattern recognition of proteins using SA6 ([SC4A]/[CD] = 0.5/1.0 μM, 1.0/1.0 μM, 2.0/1.0 μM, and 3.0/1.0 μM, [LCG] = [SC4A]). (a) Fluorescence response patterns of **SA6** against various proteins (23.8 μg/mL for each protein). (b) Canonical score plot for the two factors of simplified fluorescence response patterns obtained from LDA with 95% confidence ellipses. Error bars in (a) represent mean ± s.d. ($n = 6$ independent experiments).

Supplementary Table 9. The fluorescence response values calculated according to the assumed binding constants using a simulated sensor array based on adjusting reporter pair ratio.

Assumed binding constant between receptor and dye / M^{-1}		Sensor unit concentration / M	Calculated free dye concentration (I_0) / M	Assumed binding constant between receptor and analyte / M^{-1}		Analyte concentration / M	Calculated free dye concentration (I) / M	I/I_0
receptor 1<br>dye 1<br>(sensor unit 1)	5.0×10^{7}	0.8×10^{-6}<br>/<br>1.0×10^{-6}	2.6×10^{-7}	receptor 1<br>analyte 1	8.0×10^{6}	1.0×10^{-6}	4.1×10^{-7}	1.6
				receptor 1<br>analyte 2	2.0×10^{3}		2.6×10^{-7}	1.0
				receptor 1<br>analyte 3	9.0×10^{7}		6.8×10^{-7}	2.6
receptor 1<br>dye 1<br>(sensor unit 2)	5.0×10^{7}	1.0×10^{-6}<br>/<br>1.0×10^{-6}	1.3×10^{-7}	receptor 1<br>analyte 1	8.0×10^{6}		3.1×10^{-7}	2.4
				receptor 1<br>analyte 2	2.0×10^{3}		1.3×10^{-7}	1.0
				receptor 1<br>analyte 3	9.0×10^{7}		5.8×10^{-7}	4.5
receptor 1<br>dye 1<br>(sensor unit 3)	5.0×10^{7}	1.2×10^{-6}<br>/<br>1.0×10^{-6}	7.0×10^{-8}	receptor 1<br>analyte 1	8.0×10^{6}		2.3×10^{-7}	3.2
				receptor 1<br>analyte 2	2.0×10^{3}		6.9×10^{-8}	1.0
				receptor 1<br>analyte 3	9.0×10^{7}		4.8×10^{-7}	6.9

Supplementary Table 10. Six random numbers generated from the calculated fluorescence response values from Supplementary Table 9, used to construct the data matrix for LDA analysis.

	sensor unit 1	sensor unit 2	sensor unit 3
analyte 1	1.686801	2.477268	3.216526
analyte 1	1.735480	2.393681	3.191510
analyte 1	1.585062	2.438503	3.188072
analyte 1	1.732385	2.250631	3.311931
analyte 1	1.476016	2.354554	3.246433
analyte 1	1.755760	2.401075	3.002187
analyte 2	1.042338	1.036435	1.003990
analyte 2	1.018266	1.025412	0.925123
analyte 2	0.974137	0.952303	0.996331
analyte 2	0.957218	1.129766	0.967559
analyte 2	1.022413	1.138351	1.055565
analyte 2	0.925618	0.884566	0.926983
analyte 3	2.627027	4.425769	6.821134
analyte 3	2.420831	4.468445	7.118442
analyte 3	2.323757	4.428877	7.058280
analyte 3	2.577258	4.451951	6.872576
analyte 3	2.610911	4.610638	6.814010
analyte 3	2.670562	4.338083	6.898650

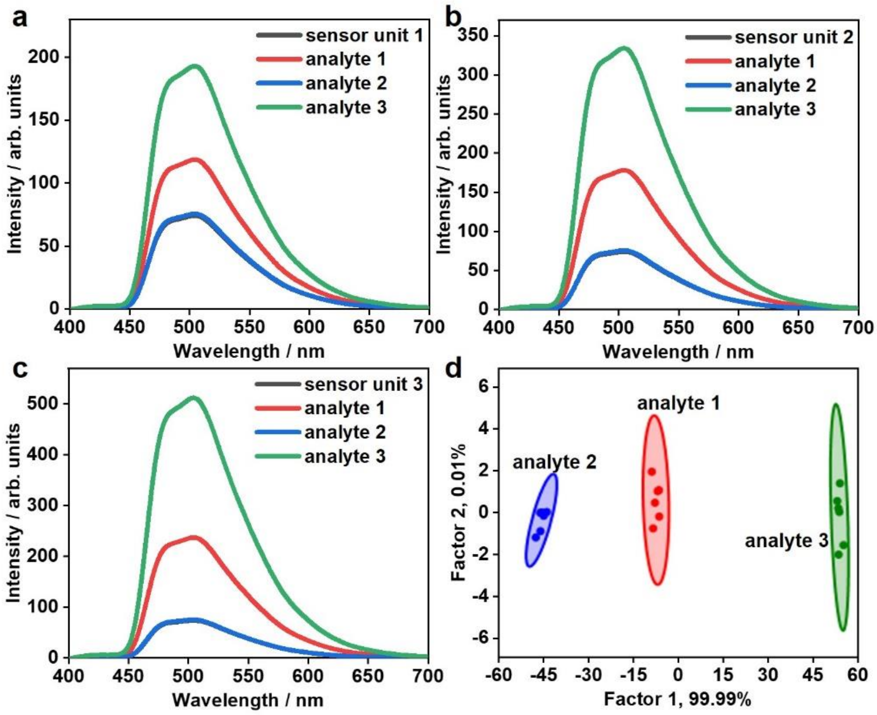

Supplementary Figure 33. Theoretically model of sensor array based on adjusting reporter pair ratio. (a–c) Theoretical simulated fluorescence spectra produced by sensor units of a sensor array formed by adjusting reporter pair ratio after adding analytes. (d) Canonical score plot for the two factors of simplified fluorescence response patterns obtained from LDA with 95% confidence ellipses (n = 6) based on the simulated data.

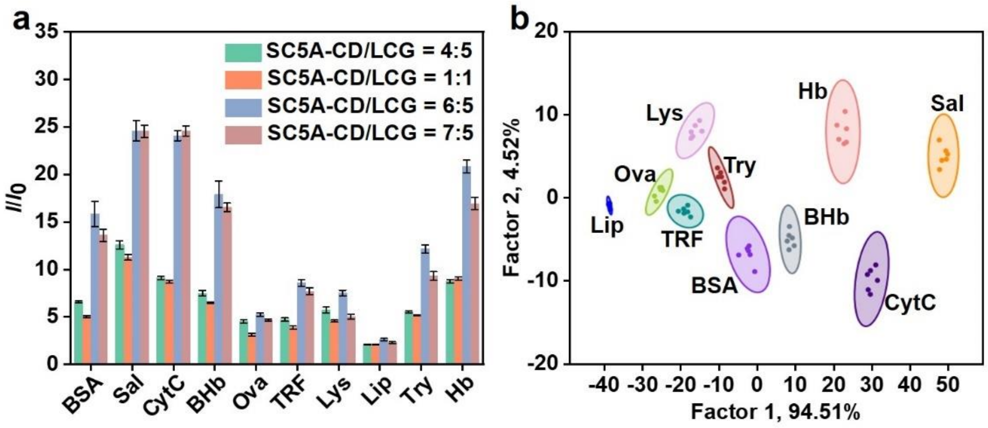

Supplementary Figure 34. Pattern recognition of proteins using SA8 ([SC5A] = [CD] = 0.8 μM, 1.0 μM, 1.2 μM and 1.4 μM, [LCG] = 1.0 μM). (a) Fluorescence response patterns of **SA8** against various proteins (23.8 μg/mL for each protein). (b) Canonical score plot for the two factors of simplified fluorescence response patterns obtained from LDA with 95% confidence ellipses. Error bars in (a) represent mean ± s.d. (n = 6 independent experiments).

Supplementary Table 11. The fluorescence response values calculated according to the assumed binding constants using a simulated sensor array based on changing pH.

Assumed binding constant between receptor and dye / M^{-1}		Sensor unit concentration / M	Calculated free dye concentration (I_0) / M	Assumed binding constant between receptor and analyte / M^{-1}		Analyte concentration / M	Calculated free dye concentration (I) / M	I/I_0
receptor 1 dye 1 at pH = 5 (sensor unit 1)	3.0×10^{7}	1.0×10^{-6}	1.7×10^{-7}	receptor 1 analyte 1 at pH = 5	8.0×10^{7}	1.0×10^{-6}	6.3×10^{-7}	3.7
				receptor 1 analyte 2 at pH = 5	4.0×10^{5}		1.9×10^{-7}	1.1
				receptor 1 analyte 3 at pH = 5	2.0×10^{3}		1.7×10^{-7}	1.0
receptor 1 dye 1 at pH = 7 (sensor unit 2)	5.0×10^{7}	1.0×10^{-6}	1.3×10^{-7}	receptor 1 analyte 1 at pH = 7	5.0×10^{6}		2.7×10^{-7}	2.1
				receptor 1 analyte 2 at pH = 7	7.0×10^{6}		3.0×10^{-7}	2.3
				receptor 1 analyte 3 at pH = 7	3.0×10^{5}		1.5×10^{-7}	1.1
receptor 1 dye 1 at pH = 9 (sensor unit 3)	7.0×10^{7}	1.0×10^{-6}	1.1×10^{-7}	receptor 1 analyte 1 at pH = 9	9.0×10^{4}		1.2×10^{-7}	1.1
				receptor 1 analyte 2 at pH = 9	5.0×10^{7}		4.7×10^{-7}	4.2
				receptor 1 analyte 3 at pH = 9	4.0×10^{6}		2.2×10^{-7}	2.0

Supplementary Table 12. Six random numbers generated from the calculated fluorescence response values from Supplementary Table 11, used to construct the data matrix for LDA analysis.

	sensor unit 1	sensor unit 2	sensor unit 3
analyte 1	3.677524	2.120472	0.868865
analyte 1	3.979513	1.964654	1.130376
analyte 1	3.453265	2.127281	1.124512
analyte 1	3.755360	2.173157	1.081120
analyte 1	3.782671	1.978566	1.135293
analyte 1	3.642261	1.999258	1.091594
analyte 2	1.224332	2.348987	4.211619
analyte 2	0.964232	2.283396	4.368789
analyte 2	1.191700	2.239501	4.163483
analyte 2	1.259797	2.139790	4.051166
analyte 2	1.043701	2.361070	4.137996
analyte 2	1.113655	2.275267	4.325064
analyte 3	0.984659	0.944343	1.903427
analyte 3	1.124381	1.067551	1.961118
analyte 3	0.900758	1.038274	2.017147
analyte 3	0.970728	1.153515	1.936084
analyte 3	0.971111	1.138907	2.051059
analyte 3	1.024820	1.148728	2.038264

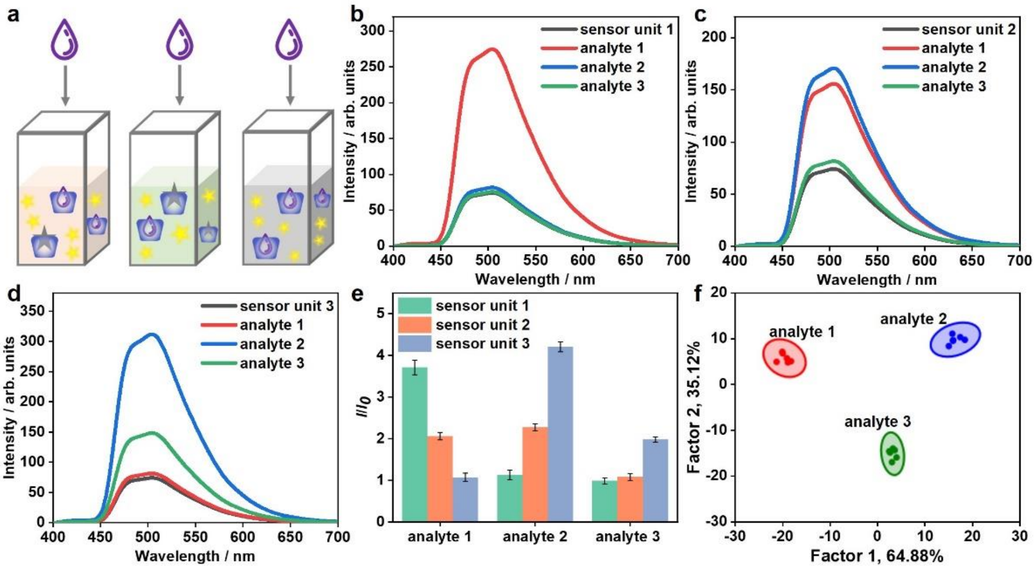

Supplementary Figure 35. Theoretically model and experimental results of sensor array based on changing the environmental factor. (a) Schematic diagram of the theoretical model of a sensor array formed by changing pH. (b–d) Theoretical simulated fluorescence spectra produced by sensor units after adding analytes. (e) Fluorescence response patterns of the simulated sensor array. (f) Canonical score plot for the two factors of simplified fluorescence response patterns obtained from LDA with 95% confidence ellipses based on the simulated data. Error bars in (e) represent mean ± s.d. ($n = 6$ random numbers).

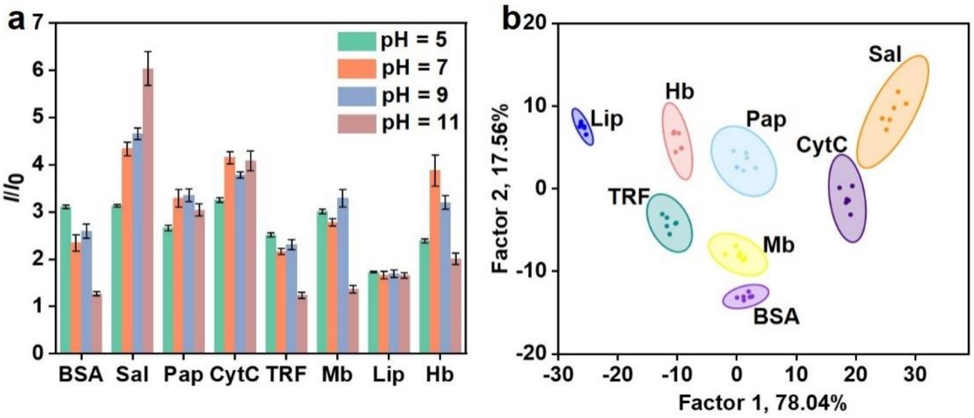

Supplementary Figure 36. Pattern recognition of proteins using SA10 ([SC5A] = [CD] = 0.6 μM, [LCG] = 1.0 μM). (a) Fluorescence response patterns of **SA10** against various proteins (23.8 μg/mL for each protein). (b) Canonical score plot for the two factors of simplified fluorescence response patterns obtained from LDA with 95% confidence ellipses. Error bars in (a) represent mean ± s.d. ($n = 6$ independent experiments).

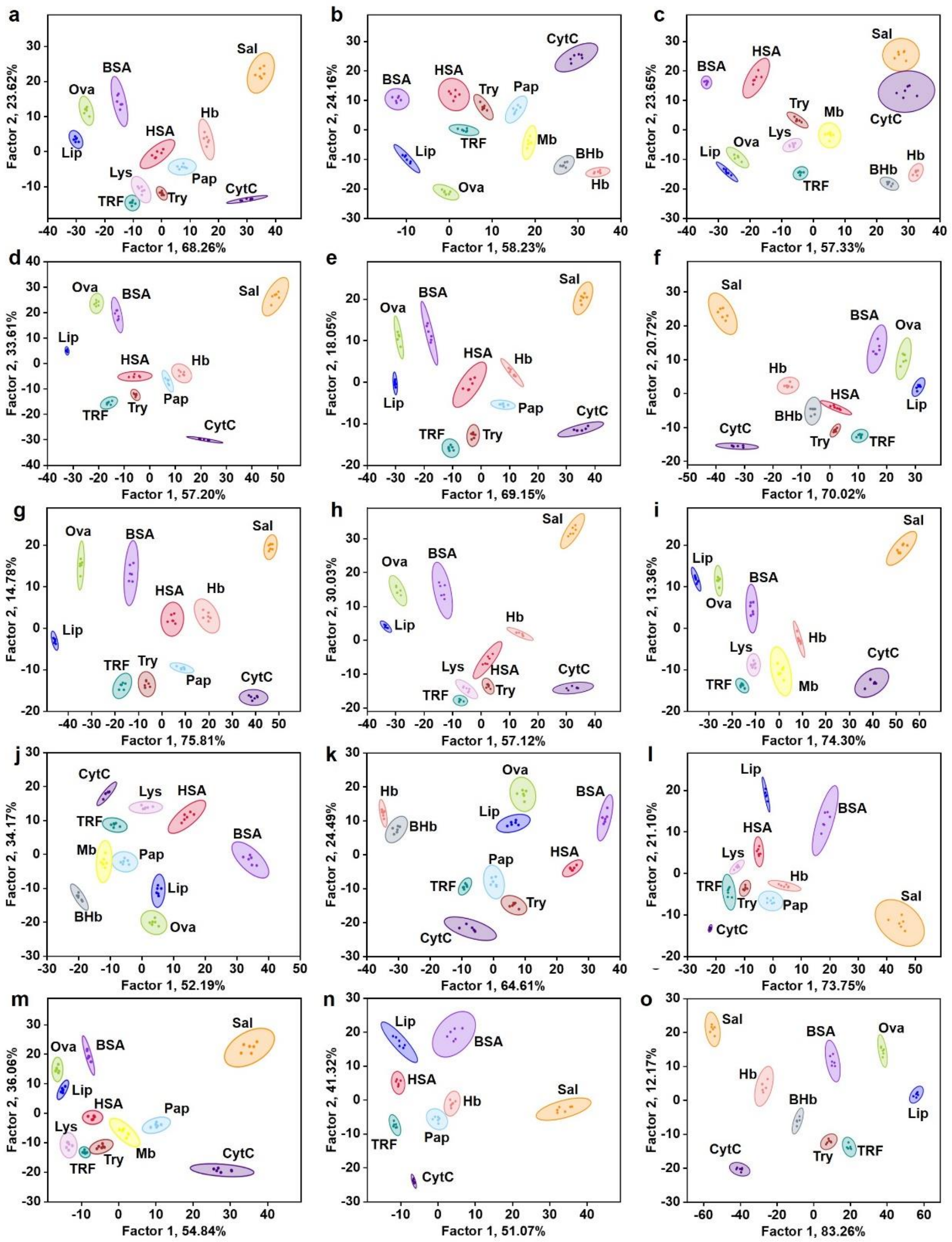

Supplementary Figure 37. Pattern recognition of proteins by combining two different sensor arrays. (a) **SA1** + **SA2**, (b) **SA1** + **SA3**, (c) **SA1** + **SA4**, (d) **SA1** + **SA5**, (e) **SA1** + **SA6**, (f) **SA1** + **SA7**, (g) **SA1** + **SA8**, (h) **SA1** + **SA9**, (i) **SA1** + **SA10**, (j) **SA2** + **SA3**, (k) **SA2** + **SA4**, (l) **SA2** + **SA5**, (m) **SA2** + **SA6**, (n) **SA2** + **SA7** and (o) **SA2** + **SA8**. Canonical score plots for the two factors of simplified fluorescence response patterns obtained from LDA with 95% confidence ellipses (n = 6).

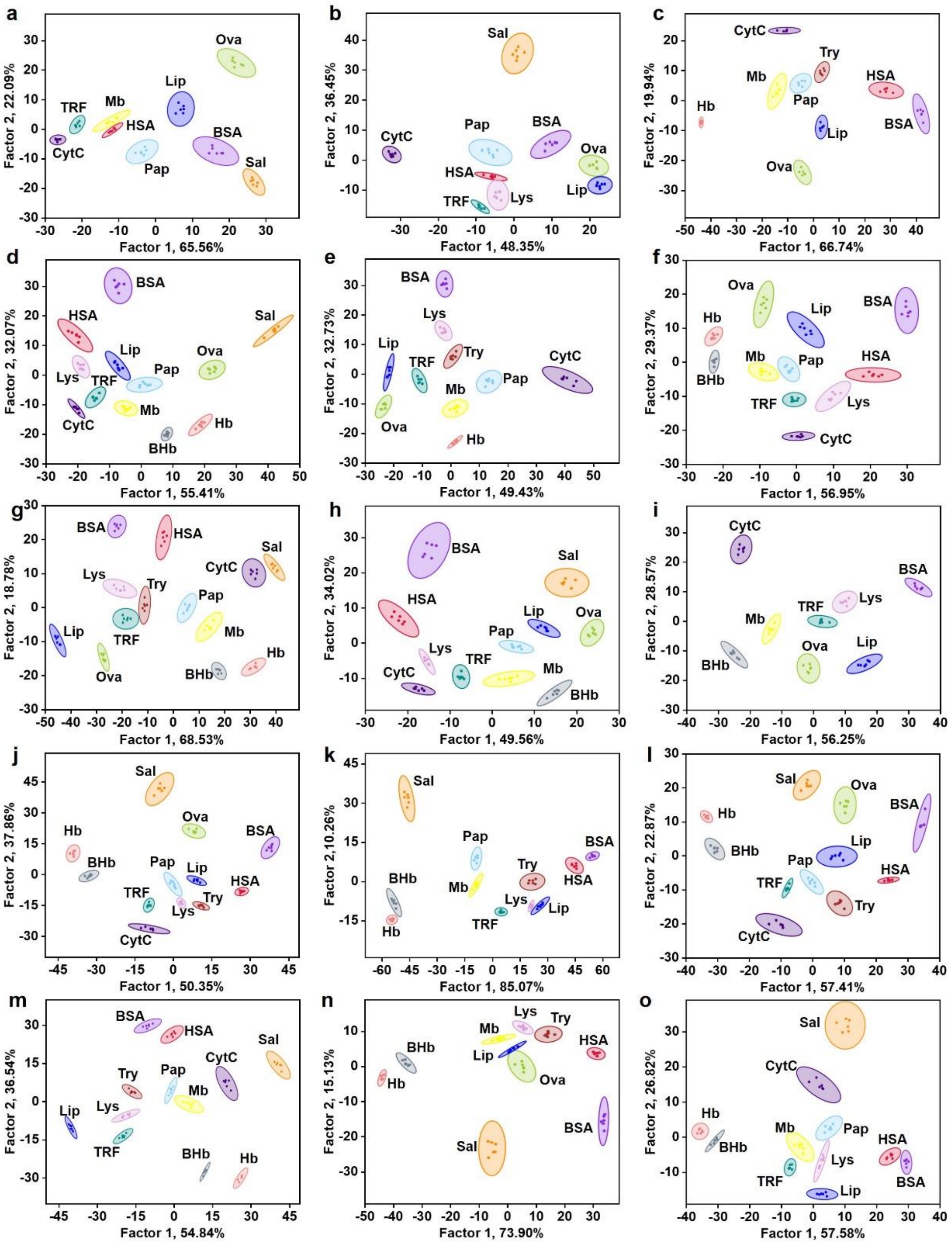

Supplementary Figure 38. Pattern recognition of proteins by combining two different sensor arrays. (a) **SA2** + **SA9**, (b) **SA2** + **SA10**, (c) **SA3** + **SA4**, (d) **SA3** + **SA5**, (e) **SA3** + **SA6**, (f) **SA3** + **SA7**, (g) **SA3** + **SA8**, (h) **SA3** + **SA9**, (i) **SA3** + **SA10**, (j) **SA4** + **SA5**, (k) **SA4** + **SA6**, (l) **SA4** + **SA7**, (m) **SA4** + **SA8**, (n) **SA4** + **SA9** and (o) **SA4** + **SA10**. Canonical score plots for the two factors of simplified fluorescence response patterns obtained from LDA with 95% confidence ellipses (n = 6).

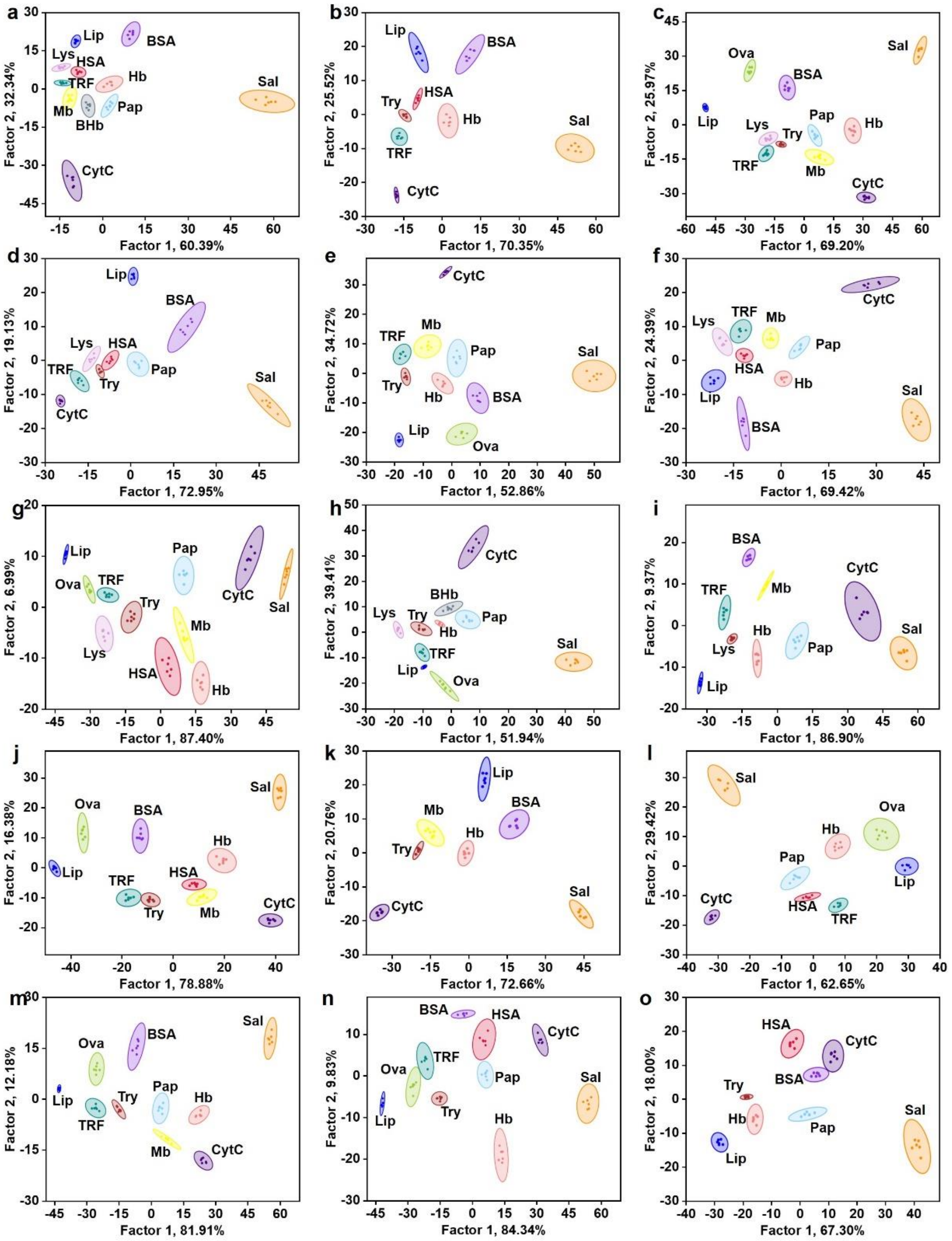

Supplementary Figure 39. Pattern recognition of proteins by combining two different sensor arrays. (a) **SA5** + **SA6**, (b) **SA5** + **SA7**, (c) **SA5** + **SA8**, (d) **SA5** + **SA9**, (e) **SA5** + **SA10**, (f) **SA6** + **SA7**, (g) **SA6** + **SA8**, (h) **SA6** + **SA9**, (i) **SA6** + **SA10**, (j) **SA7** + **SA8**, (k) **SA7** + **SA9**, (l) **SA7** + **SA10**, (m) **SA8** + **SA9**, (n) **SA8** + **SA10** and (o) **SA9** + **SA10**. Canonical score plots for the two factors of simplified fluorescence response patterns obtained from LDA with 95% confidence ellipses (n = 6).

Supplementary Table 13. The discriminant results obtained by combining two sensor arrays. The numbers represent the kind of proteins that can be distinguished on the LDA plot. The content of the diagonal line represents the recognition capability of the single sensor array itself. Purple-marked groups represent recognition capability better than the two original sensor arrays.

	SA1	SA2	SA3	SA4	SA5	SA6	SA7	SA8	SA9	SA10
**SA1**	11	11	11	12	10	10	10	10	10	9
**SA2**	-	9	10	10	10	11	8	9	9	9
**SA3**	-	-	10	9	12	10	11	13	11	8
**SA4**	-	-	-	9	12	11	11	12	10	11
**SA5**	-	-	-	-	8	11	8	11	9	10
**SA6**	-	-	-	-	-	9	10	11	10	9
**SA7**	-	-	-	-	-	-	8	10	7	8
**SA8**	-	-	-	-	-	-	-	10	10	10
**SA9**	-	-	-	-	-	-	-	-	8	8
**SA10**	-	-	-	-	-	-	-	-	-	8

Supplementary Table 14. The fluorescence response value calculated according to the assumed binding constant for identifying two analytes at two concentrations.

Assumed binding constant between coassembly and dye / M^{-1}		Sensor unit concentration / M	Calculated free dye concentration (I_0) / M	Assumed binding constant between coassembly and analyte / M^{-1}		Analyte concentration / M	Calculated free dye concentration (I) / M	I/I_0
coassembly 1<br>dye 1<br>(sensor unit 1)	5.0×10^{6}	1.0×10^{-6}	3.6×10^{-7}	coassembly 1<br>analyte 1	4.5×10^{7}	5.0×10^{-7}	5.8×10^{-7}	1.6
						8.0×10^{-6}	9.9×10^{-7}	2.7
				coassembly 1<br>analyte 2	4.0×10^{5}	5.0×10^{-7}	3.8×10^{-7}	1.1
						8.0×10^{-6}	5.8×10^{-7}	1.6
coassembly 2<br>dye 2<br>(sensor unit 2)	5.0×10^{8}	1.0×10^{-6}	4.0×10^{-8}	coassembly 2<br>analyte 1	1.4×10^{6}	5.0×10^{-7}	5.6×10^{-8}	1.4
						8.0×10^{-6}	1.4×10^{-7}	3.6
				coassembly 2<br>analyte 2	3.0×10^{7}	5.0×10^{-7}	1.4×10^{-7}	3.6
						8.0×10^{-6}	4.8×10^{-7}	12.1
coassembly 3<br>dye 3<br>(sensor unit 3)	5.0×10^{7}	1.0×10^{-6}	1.3×10^{-7}	coassembly 3<br>analyte 1	5.0×10^{6}	5.0×10^{-7}	2.1×10^{-7}	1.6
						8.0×10^{-6}	5.7×10^{-7}	4.4
				coassembly 3<br>analyte 1	5.0×10^{6}	5.0×10^{-7}	2.1×10^{-7}	1.6
						8.0×10^{-6}	5.7×10^{-7}	4.4

Supplementary Table 15. Six random numbers generated from the calculated fluorescence response values from Supplementary Table 14, used to construct the data matrix for LDA analysis.

	sensor unit 1	sensor unit 2	Sensor unit 3
analyte 1 at 0.5 μM	1.616105	1.488000	1.543349
analyte 1 at 0.5 μM	1.507372	1.475253	1.683625
analyte 1 at 0.5 μM	1.463241	1.312763	1.685623
analyte 1 at 0.5 μM	1.731771	1.344667	1.535670
analyte 1 at 0.5 μM	1.548921	1.429951	1.618619
analyte 1 at 0.5 μM	1.599704	1.329035	1.732664
analyte 1 at 8.0 μM	2.824003	3.715181	4.305635
analyte 1 at 8.0 μM	2.679547	3.665476	4.469351
analyte 1 at 8.0 μM	2.760105	3.540365	4.316767
analyte 1 at 8.0 μM	2.627940	3.573846	4.375038
analyte 1 at 8.0 μM	2.766361	3.613402	4.348609
analyte 1 at 8.0 μM	2.784051	3.687173	4.334689
analyte 2 at 0.5 μM	1.192128	3.571151	1.767527
analyte 2 at 0.5 μM	1.238225	3.691674	1.875495
analyte 2 at 0.5 μM	0.889861	3.537076	1.713060
analyte 2 at 0.5 μM	1.041197	3.677315	1.761299
analyte 2 at 0.5 μM	1.137124	3.482836	1.596488
analyte 2 at 0.5 μM	1.084252	3.623888	1.624680
analyte 2 at 8.0 μM	1.626627	12.08793	4.421919
analyte 2 at 8.0 μM	1.721133	12.02446	4.477671
analyte 2 at 8.0 μM	1.713867	11.98455	4.257318
analyte 2 at 8.0 μM	1.784775	12.23066	4.305096
analyte 2 at 8.0 μM	1.692704	12.02744	4.422452
analyte 2 at 8.0 μM	1.545784	12.08108	4.484581

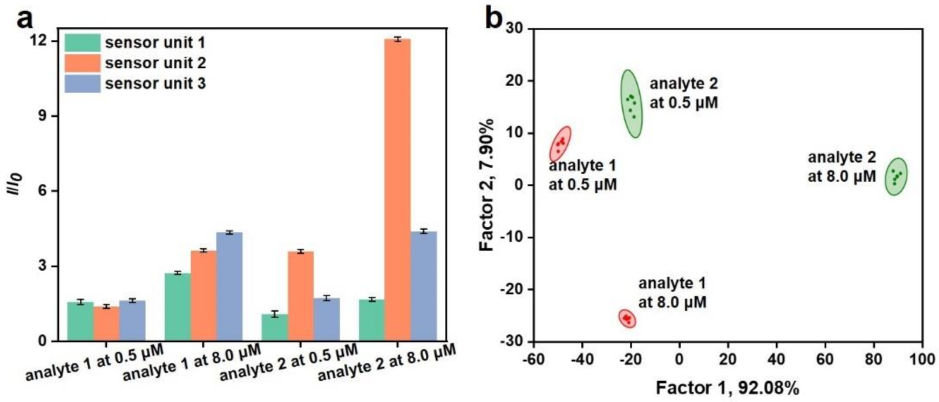

Supplementary Figure 40. Theoretically model of the sensor array for identifying two analytes at two concentrations. (a) Fluorescence response patterns of the simulated sensor array. (b) Canonical score plot for the two factors of simplified fluorescence response patterns obtained from LDA with 95% confidence ellipses (n = 6) based on the simulated data. Error bars in (a) represent mean ± s.d. (n = 6 random numbers).

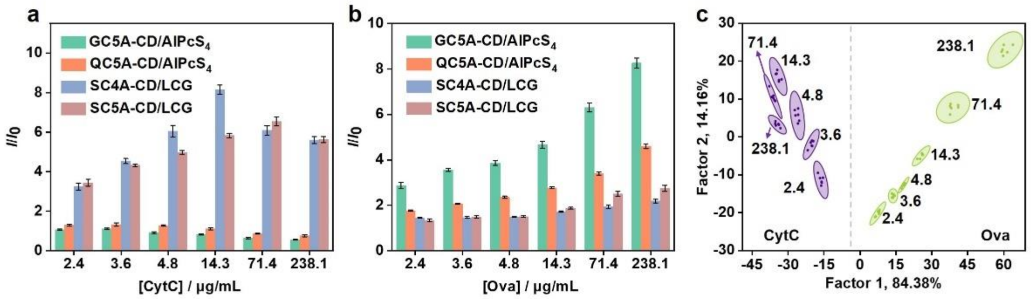

Supplementary Figure 41. Pattern recognition of different concentrations of CytC and Ova using SA1. (a) Fluorescence response patterns of the sensor array against CytC and (b) Ova at 2.4, 3.6, 4.8, 14.3, 71.4 and 238.1 μg/mL. (c) Canonical score plot for the two factors of simplified fluorescence response patterns obtained from LDA with 95% confidence ellipses. Error bars in (a) and (b) represent mean ± s.d. (n = 6 independent experiments).

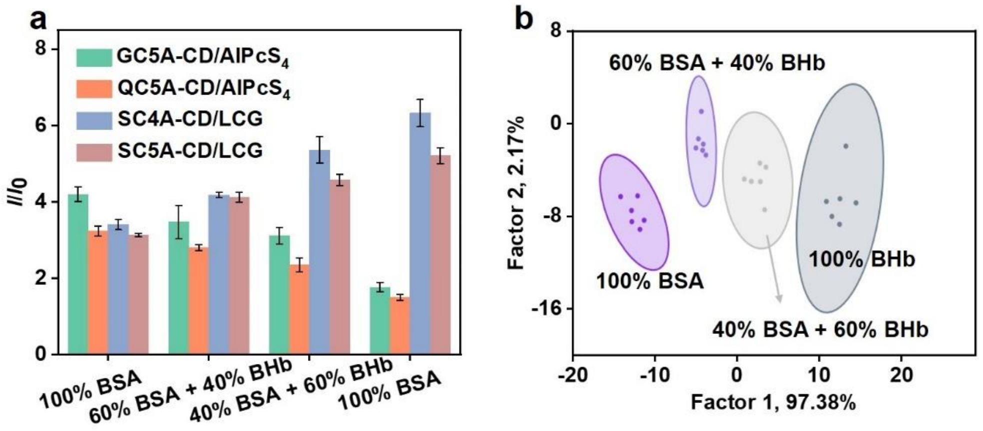

Supplementary Figure 42. Pattern recognition of BSA/BHb mixtures using SA1. (a) Fluorescence response patterns of the sensor array against protein mixtures of BSA/BHb. The total protein concentration was 23.8 μg/mL for each group. (b) Canonical score plot for the two factors of simplified fluorescence response patterns obtained from LDA with 95% confidence ellipses. Error bars in (a) represent mean ± s.d. (n = 6 independent experiments).

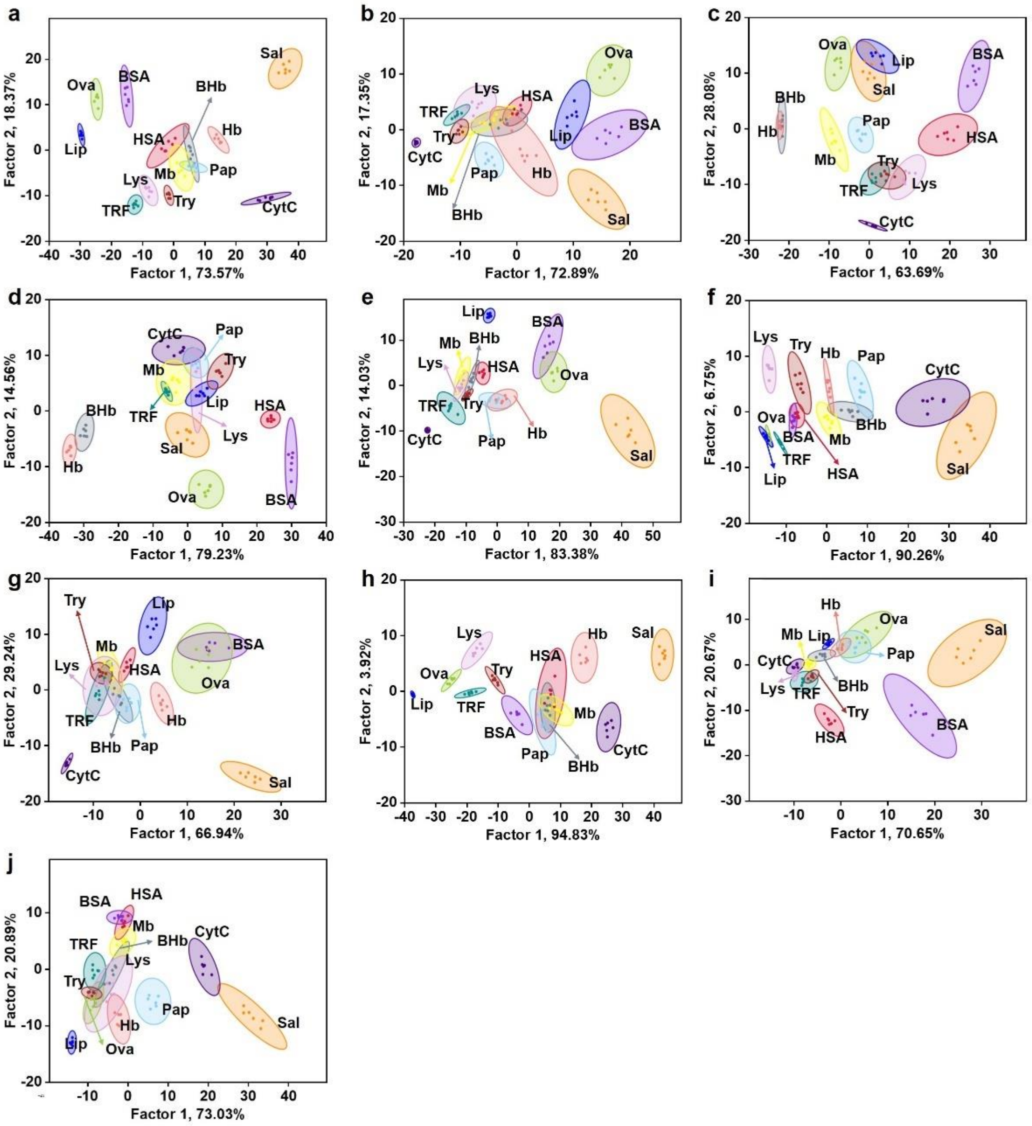

Supplementary Figure 43. Pattern recognition of all proteins used in this work. Canonical score plots for the two factors of simplified fluorescence response patterns obtained from LDA with 95% confidence ellipses (*n* = 6) of (a) **SA1**, (b) **SA2**, (c) **SA3**, (d) **SA4**, (e) **SA5**, (f) **SA6**, (g) **SA7**, (h) **SA8**, (i) **SA9** and (j) **SA10**.

Supplementary Table 16. The training matrix of fluorescence response patterns of SA1 against different proteins.

Protein	GC5A-CD/AlPcS$_4$	QC5A-CD/AlPcS$_4$	SC4A-CD/LCG	SC5A-CD/LCG
HSA	3.030367	3.043692	3.743149	6.367029
HSA	3.105192	2.921329	4.327616	7.008830
HSA	2.978363	2.864835	3.518258	6.711394
HSA	3.267904	2.943896	3.929987	7.470438
HSA	3.001677	2.803340	3.746753	6.573981
HSA	3.144308	3.252039	3.741115	6.971243
BSA	4.730562	4.800452	3.679684	5.156270
BSA	4.548590	5.440759	4.123787	5.047799
BSA	4.640615	4.426864	3.905378	4.999652
BSA	4.423808	4.283425	4.008196	5.044154
BSA	4.317464	4.896654	3.678112	5.075784
BSA	4.234400	4.581649	3.947863	4.774627
BHb	2.664351	2.190222	8.714060	6.468374
BHb	2.498799	2.103128	8.832018	6.561805
BHb	2.848342	1.932228	9.431495	6.504149
BHb	2.881593	2.654818	9.065423	6.519093
BHb	2.947968	2.615820	9.331243	6.381483
BHb	2.783517	2.624648	8.619654	6.619325
Ova	4.225398	6.354583	2.520231	3.273636
Ova	4.236543	6.426637	2.427176	3.195864
Ova	4.169384	6.881431	2.545876	3.174909
Ova	4.287905	6.268011	2.517008	3.221916
Ova	4.090954	6.514357	2.577729	2.892264
Ova	3.871611	6.382613	2.514279	3.019937
Sal	4.140029	3.263865	17.877438	11.389316
Sal	4.391379	3.492955	19.873053	11.204427
Sal	4.355320	3.545545	17.041580	11.714437
Sal	4.088334	3.791546	18.655255	11.125177
Sal	4.181840	3.332237	18.213185	10.834752
Sal	4.056554	3.609900	18.803699	11.338299
Pap	2.848948	0.801675	9.809021	6.044013
Pap	2.808222	0.938594	9.899080	6.367793
Pap	2.707146	0.879739	10.725011	6.187389
Pap	2.764226	0.952150	9.978216	6.237591
Pap	2.788883	0.772829	10.204298	6.058631
Pap	2.666030	0.876690	10.605511	6.304021
CytC	1.154671	0.556455	15.456310	8.702905
CytC	1.156387	0.645471	15.239940	8.926478
CytC	1.206128	0.644480	13.276620	8.592833
CytC	1.174051	0.607834	14.964263	8.902520
CytC	1.201047	0.677699	14.663917	8.673065

CytC	1.178270	0.593653	13.900080	8.503051
TRF	1.760250	1.902561	2.693956	3.691176
TRF	1.896717	1.896092	2.650878	3.846015
TRF	1.981048	1.815607	2.798704	3.991119
TRF	1.867541	1.878104	2.732574	3.720785
TRF	1.841651	1.700739	2.636168	4.079095
TRF	1.856427	1.883597	2.700654	3.910605
Mb	2.835556	1.682229	5.876151	6.554120
Mb	2.829476	1.330921	6.210066	6.816568
Mb	2.472618	1.247337	5.943893	6.800331
Mb	2.651607	2.047450	6.179689	6.784963
Mb	2.609565	1.494824	6.168472	6.610510
Mb	2.549224	1.604777	5.886037	6.469564
Lys	2.052223	2.187724	3.925902	4.656084
Lys	2.049518	1.597490	4.212898	4.480701
Lys	2.095952	2.449848	4.141870	4.579660
Lys	2.280847	1.944263	3.962225	4.504025
Lys	2.283014	1.784301	4.298387	4.458563
Lys	2.322329	1.110957	4.684620	4.712556
Lip	4.496668	1.985083	2.466147	2.067197
Lip	4.454861	1.949344	2.493900	2.137867
Lip	4.595037	1.973137	2.628330	2.077199
Lip	4.527707	1.999126	2.559539	2.115264
Lip	4.524743	1.754777	2.412274	2.083679
Lip	4.335347	1.983773	2.270209	2.106437
Try	1.896642	1.327497	5.969323	5.183314
Try	2.026457	1.358328	6.273109	5.184896
Try	1.949709	1.515731	6.190966	5.238911
Try	1.833705	1.474353	6.214021	5.182403
Try	1.948781	1.662778	5.867536	5.150553
Try	1.925907	1.796665	6.175308	5.164303
Hb	2.864823	3.376292	7.996288	8.778343
Hb	2.734847	3.326840	7.806682	8.884986
Hb	2.699794	2.978938	7.546427	9.140539
Hb	2.730818	2.870203	7.018783	9.243655
Hb	2.781060	2.793233	7.164231	9.235790
Hb	2.872550	3.009165	7.321079	8.866342

Supplementary Table 17. The training matrix of fluorescence response patterns of SA2 against different proteins.

Protein	GC4A-CD/$AlPcS_4$	GC5A-CD/$AlPcS_4$	QC4A-CD/$AlPcS_4$	QC5A-CD/$AlPcS_4$
HSA	6.909648	3.030367	4.386486	3.043692
HSA	6.480504	3.105192	4.190131	2.921329
HSA	6.472328	2.978363	4.613294	2.864835
HSA	6.180315	3.267904	4.413344	2.943896
HSA	6.299973	3.001677	4.229549	2.803340
HSA	6.257791	3.144308	4.463121	3.252039
BSA	15.402023	4.730562	6.231835	4.800452
BSA	15.935357	4.548590	6.598909	5.440759
BSA	14.427391	4.640615	6.279931	4.426864
BSA	15.474365	4.423808	6.153202	4.283425
BSA	14.889437	4.317464	6.209195	4.896654
BSA	14.514154	4.234400	5.656226	4.581649
BHb	5.530510	2.664351	3.720956	2.190222
BHb	6.075657	2.498799	3.837701	2.103128
BHb	5.983432	2.848342	3.763313	1.932228
BHb	6.649639	2.881593	3.794933	2.654818
BHb	5.908025	2.947968	3.747312	2.615820
BHb	5.831761	2.783517	3.603526	2.624648
Ova	11.755528	4.225398	7.948944	6.354583
Ova	12.090573	4.236543	8.083446	6.426637
Ova	12.100786	4.169384	7.428753	6.881431
Ova	13.126432	4.287905	7.796397	6.268011
Ova	11.956208	4.090954	7.468278	6.514357
Ova	12.673208	3.871611	7.392986	6.382613
Sal	17.452335	4.140029	3.921427	3.263865
Sal	19.479033	4.391379	4.173321	3.492955
Sal	19.112106	4.355320	4.514393	3.545545
Sal	17.353973	4.088334	4.126260	3.791546
Sal	18.892279	4.181840	4.541876	3.332237
Sal	18.773474	4.056554	4.856979	3.609900
Pap	7.011571	2.848948	3.247717	0.801675
Pap	7.966200	2.808222	3.026027	0.938594
Pap	7.135974	2.707146	2.997301	0.879739
Pap	7.008159	2.764226	2.790235	0.952150
Pap	6.149637	2.788883	3.040319	0.772829
Pap	7.133707	2.666030	2.702087	0.876690
CytC	2.247851	1.154671	1.272192	0.556455
CytC	2.006351	1.156387	1.298922	0.645471
CytC	2.143244	1.206128	1.299707	0.644480
CytC	2.070230	1.174051	1.236383	0.607834
CytC	2.218075	1.201047	1.254051	0.677699

CytC	2.267717	1.178270	1.450353	0.593653
TRF	3.124735	1.760250	2.376714	2.118349
TRF	3.239849	1.896717	2.947056	1.896092
TRF	3.315534	1.981048	2.929548	2.144185
TRF	3.426342	1.867541	3.145007	1.878104
TRF	3.472757	1.816843	3.135990	1.700739
TRF	3.288964	1.856427	3.254531	1.883597
Mb	5.126347	2.835556	4.568113	1.682229
Mb	5.178641	2.829476	4.413794	1.330921
Mb	4.881185	2.472618	4.104741	1.247337
Mb	5.634919	2.651607	4.107588	2.047450
Mb	4.805707	2.609565	3.839314	1.494824
Mb	5.152916	2.549224	3.909736	1.604777
Lys	4.093336	2.052223	4.205564	2.187724
Lys	3.913482	2.049518	4.308409	1.597490
Lys	4.941311	2.095952	4.289941	2.449848
Lys	4.717436	2.280847	4.798211	1.944263
Lys	4.304729	2.283014	4.349707	1.784301
Lys	4.259260	2.322329	4.368441	1.110957
Lip	8.886496	4.496668	7.882731	1.985083
Lip	9.999724	4.454861	7.700798	1.949344
Lip	9.604693	4.595037	7.149482	1.973137
Lip	9.058551	4.527707	6.885637	1.999126
Lip	8.523720	4.524743	6.638226	1.754777
Lip	9.805250	4.335347	6.444330	1.983773
Try	4.450724	1.896642	3.039458	1.327497
Try	4.209893	2.026457	2.621354	1.358328
Try	4.189598	1.949709	2.759455	1.515731
Try	4.628729	1.833705	2.705200	1.474353
Try	4.330946	1.948781	2.954841	1.662778
Try	4.922847	1.925907	2.749268	1.796665
Hb	12.840640	2.864823	4.205415	3.376292
Hb	11.986539	2.734847	3.711554	3.326840
Hb	8.513214	2.699794	3.951793	2.978938
Hb	10.741297	2.730818	3.955541	2.870203
Hb	11.236648	2.781060	3.821436	2.793233
Hb	11.342187	2.872550	3.289445	3.009165

Supplementary Table 18. The training matrix of fluorescence response patterns of SA3 against different proteins.

Protein	GC5A-CD/AlPcS$_4$	GC5A-CD/TPPS	GC5A-CD/1,8-ANS	GC5A-CD/PTPE
HSA	3.030367	1.449637	1.290109	1.226154
HSA	3.105192	1.475776	1.334878	1.229907
HSA	2.978363	1.506980	1.396674	1.268544
HSA	3.267904	1.524333	1.424252	1.233031
HSA	3.001677	1.478233	1.362713	1.280521
HSA	3.144308	1.468460	1.357518	1.243780
BSA	4.730562	1.366122	1.555792	1.073257
BSA	4.548590	1.460483	1.557272	1.076721
BSA	4.640615	1.439362	1.588472	1.089904
BSA	4.423808	1.373894	1.560980	1.131048
BSA	4.317464	1.413329	1.565574	1.110843
BSA	4.234400	1.399185	1.487772	0.978818
BHb	2.664351	1.179548	0.171143	0.571160
BHb	2.498799	1.118980	0.173284	0.561190
BHb	2.848342	1.062041	0.181646	0.566753
BHb	2.881593	1.081198	0.192615	0.602616
BHb	2.947968	1.105398	0.187224	0.549653
BHb	2.783517	1.030386	0.171859	0.553897
Ova	4.225398	1.385084	0.626744	1.088149
Ova	4.236543	1.437723	0.640240	1.118797
Ova	4.169384	1.438640	0.659190	1.080399
Ova	4.287905	1.380790	0.658280	1.036111
Ova	4.090954	1.375096	0.653122	1.016099
Ova	3.871611	1.409657	0.586489	0.935172
Sal	4.140029	1.318940	0.794318	0.942591
Sal	4.391379	1.291039	0.814083	0.930461
Sal	4.355320	1.299103	0.866948	0.998689
Sal	4.088334	1.284424	0.881473	1.004765
Sal	4.181840	1.272421	0.856015	1.017271
Sal	4.056554	1.250874	0.834311	0.971322
Pap	2.848948	1.307960	0.714548	0.909176
Pap	2.808222	1.310127	0.753349	0.935100
Pap	2.707146	1.249293	0.749925	0.949367
Pap	2.764226	1.381710	0.761167	0.910138
Pap	2.788883	1.413010	0.708242	0.890017
Pap	2.666030	1.336579	0.724363	0.906549
CytC	1.154671	1.000569	0.757174	0.889652
CytC	1.156387	0.960754	0.807005	0.945968
CytC	1.206128	0.919427	0.822898	0.998753
CytC	1.174051	0.950395	0.827129	0.939107
CytC	1.201047	0.912289	0.834792	0.931044

CytC	1.178270	0.924744	0.827135	0.931459
TRF	1.760250	1.449009	0.829942	1.273965
TRF	1.896717	1.436522	0.865065	1.318643
TRF	1.981048	1.404607	0.840224	1.282238
TRF	1.867541	1.598452	0.868721	1.236867
TRF	1.801949	1.426707	0.853361	1.249669
TRF	1.856427	1.358738	0.826800	1.219668
Mb	2.835556	1.219224	0.542395	0.868284
Mb	2.829476	1.135587	0.572564	0.950727
Mb	2.472618	1.162173	0.608520	0.934885
Mb	2.651607	1.159149	0.578600	0.946334
Mb	2.609565	1.201357	0.578466	0.907822
Mb	2.549224	1.209377	0.554365	0.947143
Lys	2.052223	1.313833	1.042740	1.185443
Lys	2.049518	1.260479	1.062268	1.215556
Lys	2.095952	1.169544	1.089370	1.321710
Lys	2.280847	1.279973	1.147950	1.288210
Lys	2.283014	1.231913	1.098734	1.204429
Lys	2.322329	1.196732	1.068028	1.137867
Lip	4.496668	1.432643	0.861654	1.027043
Lip	4.454861	1.430633	0.882448	1.108882
Lip	4.595037	1.365722	0.919411	1.150796
Lip	4.527707	1.393582	0.959378	1.234577
Lip	4.524743	1.419343	0.966678	1.166795
Lip	4.335347	1.417547	0.943466	1.117500
Try	1.896642	1.500063	0.879045	1.217077
Try	2.026457	1.499872	0.917686	1.296421
Try	1.949709	1.480740	0.969954	1.203956
Try	1.833705	1.513815	0.941534	1.426728
Try	1.948781	1.525342	0.917570	1.247789
Try	1.925907	1.530378	0.922880	1.196941
Hb	2.864823	1.035923	0.168908	0.547536
Hb	2.734847	1.005783	0.159507	0.542471
Hb	2.699794	1.049506	0.171029	0.537504
Hb	2.730818	1.055798	0.165792	0.539371
Hb	2.781060	1.044731	0.168212	0.553695
Hb	2.872550	1.032738	0.168766	0.500150

Supplementary Table 19. The training matrix of fluorescence response patterns of SA4 against different proteins.

Protein	QC5A-CD/AlPcS$_4$	QC5A-CD/TPPS	QC5A-CD/1,8-ANS	QC5A-CD/2,6-TNS
HSA	3.043692	0.816470	1.858396	1.474743
HSA	2.921329	0.785964	1.885942	1.458694
HSA	2.864835	0.851590	1.847759	1.515639
HSA	2.943896	0.854692	1.898839	1.509012
HSA	2.803340	0.831160	1.899980	1.478879
HSA	3.252039	0.818511	1.861305	1.518453
BSA	4.800452	0.796210	2.148063	1.559145
BSA	5.440759	0.869676	2.171731	1.494721
BSA	4.426864	0.829570	2.118784	1.522453
BSA	4.283425	0.835098	2.170936	1.533167
BSA	4.896654	0.816543	2.208187	1.481257
BSA	4.581649	0.838598	2.193129	1.493824
BHb	2.190222	0.462048	0.403069	0.335977
BHb	2.103128	0.463083	0.381045	0.327778
BHb	1.932228	0.454749	0.408191	0.337838
BHb	2.654818	0.439811	0.400534	0.360424
BHb	2.615820	0.434499	0.380988	0.345209
BHb	2.624648	0.458670	0.313885	0.340924
Ova	6.354583	0.975029	1.072639	1.214109
Ova	6.426637	0.908663	0.984505	1.238583
Ova	6.881431	0.941825	1.049012	1.259203
Ova	6.268011	1.007264	1.056344	1.291962
Ova	6.514357	0.997166	1.046632	1.276675
Ova	6.382613	0.965020	1.080936	1.285015
Sal	3.263865	0.693524	1.040673	0.977640
Sal	3.492955	0.718042	1.143828	1.007498
Sal	3.545545	0.601838	1.141637	1.048679
Sal	3.791546	0.748388	1.156452	1.063565
Sal	3.332237	0.729334	1.117355	1.061859
Sal	3.609900	0.639766	1.160035	1.069757
Pap	0.801675	0.564947	1.156639	1.139595
Pap	0.938594	0.625467	1.169316	1.183186
Pap	0.879739	0.611669	1.128387	1.196272
Pap	0.952150	0.544943	1.177076	1.212079
Pap	0.772829	0.534715	1.122890	1.181172
Pap	0.876690	0.564986	1.187759	1.211083
CytC	0.556455	0.367601	1.126801	1.126267
CytC	0.645471	0.389182	1.105244	1.118920
CytC	0.644480	0.370164	1.103031	1.142557
CytC	0.607834	0.377515	0.927427	1.078704
CytC	0.677699	0.384079	1.093340	1.099609

CytC	0.593653	0.391483	1.061439	1.035841
TRF	1.902561	0.596786	0.892529	0.995258
TRF	1.896092	0.608899	0.924409	0.985011
TRF	1.815607	0.607455	0.873899	1.005403
TRF	1.878104	0.594780	0.863822	1.011706
TRF	1.700739	0.600094	0.881141	0.990633
TRF	1.883597	0.604458	0.910087	1.009946
Mb	1.682229	0.539614	0.924285	1.003005
Mb	1.330921	0.549597	0.982181	1.010966
Mb	1.247337	0.550810	0.987710	1.022211
Mb	2.047450	0.569027	0.992421	1.048879
Mb	1.494824	0.554192	1.009766	1.063089
Mb	1.604777	0.550388	1.033622	1.068202
Lys	2.187724	0.738341	1.107287	1.155197
Lys	1.597490	0.749292	1.091575	1.154706
Lys	2.449848	0.604634	1.184628	1.183654
Lys	1.944263	0.676044	1.117194	1.187991
Lys	1.784301	0.670579	1.191954	1.127455
Lys	1.110957	0.585638	1.183081	1.160935
Lip	1.985083	0.713163	1.185315	1.204748
Lip	1.949344	0.764329	1.185786	1.205292
Lip	1.973137	0.717665	1.184797	1.262134
Lip	1.999126	0.716831	1.159717	1.172089
Lip	1.754777	0.726149	1.171562	1.175656
Lip	1.983773	0.687292	1.155095	1.147205
Try	1.327497	0.658158	1.354609	1.316340
Try	1.358328	0.622939	1.363043	1.407397
Try	1.515731	0.632514	1.309192	1.349197
Try	1.474353	0.625660	1.304674	1.323709
Try	1.662778	0.657636	1.301296	1.369157
Try	1.796665	0.683148	1.304689	1.379790
Hb	3.376292	0.391123	0.231119	0.244610
Hb	3.326840	0.472556	0.250376	0.255565
Hb	2.978938	0.467304	0.238134	0.269088
Hb	2.870203	0.453052	0.255319	0.251083
Hb	2.793233	0.477915	0.261506	0.239251
Hb	3.009165	0.452172	0.250320	0.233591

Supplementary Table 20. The training matrix of fluorescence response patterns of SA5 against different proteins.

Protein	GC5A-CD = 1:2	GC5A-CD = 1:1	GC5A-CD = 2:1	GC5A-CD = 3:1
HSA	1.575838	3.030367	4.265842	4.538413
HSA	1.588603	3.105192	4.736019	5.137348
HSA	1.565483	2.978363	4.190224	5.346757
HSA	1.378111	3.267904	4.175098	5.174000
HSA	1.621593	3.001677	4.619085	5.075009
HSA	1.635938	3.144308	4.573919	4.885102
BSA	2.282029	4.730562	8.622697	11.565558
BSA	2.369753	4.548590	8.462863	11.110106
BSA	2.441954	4.640615	7.847066	10.796577
BSA	2.318374	4.423808	8.003883	10.738866
BSA	2.278139	4.317464	7.967400	11.483932
BSA	2.146459	4.234400	7.757381	11.362748
BHb	1.526785	2.664351	3.562797	4.266270
BHb	1.500777	2.498799	3.628484	4.041875
BHb	1.401142	2.848342	3.647156	4.145122
BHb	1.468915	2.881593	3.953227	4.107696
BHb	1.492527	2.947968	3.644590	4.069096
BHb	1.387111	2.783517	3.854661	3.968742
Ova	1.998840	4.225398	7.876458	12.844419
Ova	2.076121	4.236543	7.107574	14.440823
Ova	1.975457	4.169384	8.056335	13.566398
Ova	1.988743	4.287905	8.158464	14.224771
Ova	2.011599	4.090954	7.607618	13.632520
Ova	2.056333	3.871611	7.223340	14.472949
Sal	1.954503	4.140029	11.444843	24.388390
Sal	2.123359	4.391379	11.844981	25.524634
Sal	1.865148	4.355320	11.872310	26.864342
Sal	1.872374	4.088334	11.435044	28.389140
Sal	1.897772	4.181840	12.005186	26.754628
Sal	1.969491	4.056554	11.740575	26.660480
Pap	1.490663	2.848948	5.849587	8.169425
Pap	1.356237	2.808222	5.384837	8.388453
Pap	1.483316	2.707146	5.359605	7.114521
Pap	1.416858	2.764226	5.829487	8.111203
Pap	1.450772	2.788883	5.840535	8.134585
Pap	1.411816	2.666030	5.145270	7.280973
CytC	0.762461	1.154671	1.544764	1.556553
CytC	0.810516	1.156387	1.451583	1.658025
CytC	0.762317	1.206128	1.590562	1.789558
CytC	0.796106	1.174051	1.415568	1.637819
CytC	0.770080	1.201047	1.423660	1.554555

CytC	0.790664	1.178270	1.477269	1.702749
TRF	1.175358	1.760250	2.526871	2.977949
TRF	1.200248	1.896717	2.611355	2.819198
TRF	1.196974	1.981048	2.659089	2.949203
TRF	1.296452	1.867541	5.305665	3.161614
TRF	1.233714	1.801949	2.608750	2.949377
TRF	1.247897	1.856427	2.360854	3.070478
Mb	1.442892	2.835556	3.002180	2.763921
Mb	1.490339	2.829476	2.885023	3.094051
Mb	1.471857	2.472618	3.068869	3.010332
Mb	1.457281	2.651607	2.803158	2.806439
Mb	1.537587	2.609565	3.049715	2.566554
Mb	1.478515	2.549224	2.707676	2.666229
Lys	1.767962	2.052223	2.499393	2.948335
Lys	1.750265	2.049518	2.575175	2.700847
Lys	1.649966	2.095952	2.645369	3.120699
Lys	1.564199	2.280847	2.891935	3.282657
Lys	1.653067	2.283014	2.618223	3.119192
Lys	1.748990	2.322329	2.477853	3.154337
Lip	1.751231	4.496668	3.583089	3.614532
Lip	1.665431	4.454861	3.233528	3.400676
Lip	1.634815	4.595037	3.558892	3.156575
Lip	1.710225	4.527707	3.668536	3.410212
Lip	1.799041	4.524743	3.741907	3.227976
Lip	1.867183	4.335347	3.615568	3.203540
Try	1.816707	1.896642	2.660348	4.125895
Try	1.749235	2.026457	2.914241	4.168690
Try	1.818708	1.949709	2.810808	4.343600
Try	2.086352	1.833705	2.735475	4.211608
Try	1.925973	1.948781	2.856965	4.146023
Try	1.874879	1.925907	2.887521	4.007095
Hb	1.463750	2.864823	5.787124	8.907718
Hb	1.482209	2.734847	4.913827	8.276339
Hb	1.562259	2.699794	5.118656	9.100510
Hb	1.586748	2.730818	4.875036	9.274969
Hb	1.691357	2.781060	5.064673	9.530486
Hb	1.649402	2.872550	4.931190	9.263452

Supplementary Table 21. **The training matrix of fluorescence response patterns of SA6 against different proteins.**

Protein	SC4A-CD = 1:2	SC4A-CD = 1:1	SC4A-CD = 2:1	SC4A-CD = 3:1
HSA	3.658848	3.743149	9.562974	10.061685
HSA	3.957308	4.327616	10.783083	11.416128
HSA	4.145507	3.518258	11.978692	12.805898
HSA	3.954421	3.929987	11.503177	12.704818
HSA	3.868672	3.746753	11.272964	11.473482
HSA	4.004509	3.741115	12.196153	13.238228
BSA	3.530305	3.679684	9.959146	10.559483
BSA	3.496772	4.123787	9.700216	9.671996
BSA	3.789877	3.905378	10.643201	9.840203
BSA	3.704220	4.008196	9.830978	9.903716
BSA	3.332251	3.678112	10.085041	9.617672
BSA	3.615807	3.947863	10.350136	10.219079
BHb	4.798475	8.714060	17.362407	16.797496
BHb	4.673653	8.832018	17.070892	17.721971
BHb	4.890809	9.431495	18.642305	17.836926
BHb	4.576827	9.065423	17.638402	16.410396
BHb	4.676597	9.331243	16.955150	16.033371
BHb	4.492255	8.619654	15.336603	13.887055
Ova	2.198948	2.520231	3.984380	4.290608
Ova	2.142065	2.427176	4.490333	4.605685
Ova	2.247125	2.545876	4.166940	4.459810
Ova	2.312345	2.517008	4.463782	4.517303
Ova	2.033427	2.577729	4.252030	4.508739
Ova	2.227662	2.514279	4.042270	4.765421
Sal	4.363933	17.877438	25.710589	35.004345
Sal	4.488133	19.873053	26.318879	38.651447
Sal	4.371292	17.041580	27.718729	40.464222
Sal	5.442112	18.655255	29.193848	39.920048
Sal	5.521588	18.213185	30.629462	40.281433
Sal	5.484294	18.803699	29.168855	35.433350
Pap	5.292771	9.809021	17.942492	22.044623
Pap	5.504191	9.899080	17.697372	23.510378
Pap	5.520624	10.725011	16.442510	22.054959
Pap	5.484976	9.978216	18.009511	23.225770
Pap	5.841333	10.204298	17.073195	18.236130
Pap	5.479127	10.605511	18.212367	20.212824
CytC	6.720128	15.456310	30.076146	40.922907
CytC	6.783582	15.239940	30.892379	39.968828
CytC	6.682406	13.276620	29.318662	37.103399
CytC	6.348719	14.964263	27.170901	39.088818
CytC	6.154553	14.663917	26.385614	37.296819

CytC	6.070256	13.900080	28.736123	35.530454
TRF	2.386257	2.693956	7.105127	7.270978
TRF	2.443986	2.650878	6.858434	7.878612
TRF	2.308549	2.798704	7.052387	8.080317
TRF	2.360134	2.732574	7.059952	8.715293
TRF	2.389842	2.636168	7.467668	8.506717
TRF	2.283585	2.700654	7.974012	8.639645
Mb	4.430127	5.876151	15.665875	16.333480
Mb	4.459195	6.210066	15.659327	16.519015
Mb	4.111368	5.943893	16.285237	18.360610
Mb	4.372385	6.179689	15.863969	17.052066
Mb	4.482101	6.168472	17.163766	18.100179
Mb	4.496382	5.886037	17.614913	19.053028
Lys	4.790307	3.925902	4.587846	4.197608
Lys	4.625545	4.212898	4.239419	3.874674
Lys	4.740614	4.141870	4.399962	3.831852
Lys	4.158634	3.962225	4.180176	3.897710
Lys	4.585634	4.298387	4.236762	3.766547
Lys	4.537350	4.684620	4.075121	3.524450
Lip	2.022713	2.466147	3.545383	3.745168
Lip	2.071523	2.493900	3.380361	3.965630
Lip	1.958347	2.628330	3.716590	3.499106
Lip	2.092842	2.559539	3.286935	3.685009
Lip	2.038527	2.412274	3.176026	3.614748
Lip	2.177640	2.270209	3.298195	3.283524
Try	4.865099	5.969323	8.266698	7.991150
Try	4.649145	6.273109	9.047727	8.659233
Try	4.371034	6.190966	9.313988	8.874754
Try	5.082219	6.214021	9.382963	8.750918
Try	4.633335	5.867536	8.965418	7.901221
Try	4.453011	6.175308	8.944938	7.967278
Hb	5.000449	7.996288	13.965795	12.062055
Hb	5.482410	7.806682	14.367082	13.304446
Hb	4.982501	7.546427	15.056424	13.878626
Hb	5.757540	7.018783	16.429634	14.911177
Hb	5.815531	7.164231	16.481608	15.318764
Hb	5.575532	7.321079	16.784967	12.591227

Supplementary Table 22. **The training matrix of fluorescence response patterns of SA7 against different proteins.**

Protein	GC5A-CD/AlPcS$_4$ = 4:5	GC5A-CD/AlPcS$_4$ = 1:1	GC5A-CD/AlPcS$_4$ = 6:5	GC5A-CD/AlPcS$_4$ = 7:5
HSA	1.422136	3.030367	4.396806	4.721867
HSA	1.530840	3.105192	4.221437	5.102339
HSA	1.479637	2.978363	4.532363	5.059015
HSA	1.465120	3.267904	4.143648	4.660898
HSA	1.510847	3.001677	4.262358	4.844979
HSA	1.435749	3.144308	4.711671	4.815503
BSA	1.720601	4.730562	8.226835	12.380576
BSA	1.776834	4.548590	8.673537	13.996628
BSA	1.721386	4.640615	7.866657	11.873308
BSA	1.677143	4.423808	8.786987	12.136022
BSA	1.724110	4.317464	8.000958	11.857827
BSA	1.718769	4.234400	8.233808	12.034235
BHb	1.319044	2.664351	3.525955	4.259485
BHb	1.300772	2.498799	3.193729	4.328981
BHb	1.181910	2.848342	3.737150	5.058278
BHb	1.239796	2.881593	3.616423	4.823856
BHb	1.224618	2.947968	3.885796	4.668044
BHb	1.214210	2.783517	3.635911	5.169031
Ova	1.694211	4.225398	6.780269	10.905835
Ova	1.739754	4.236543	7.565680	12.076806
Ova	1.714554	4.169384	7.338409	12.280312
Ova	1.602551	4.287905	6.577026	12.427563
Ova	1.647812	4.090954	6.396139	11.525296
Ova	1.601469	3.871611	6.500397	10.997170
Sal	0.938634	4.140029	7.367841	17.571883
Sal	0.899533	4.391379	9.467970	18.994093
Sal	0.903188	4.355320	10.080253	19.569905
Sal	0.907371	4.088334	9.465281	18.239110
Sal	0.922037	4.181840	10.371770	18.375093
Sal	0.907864	4.056554	9.901333	19.267815
Pap	1.223355	2.848948	5.198945	6.132689
Pap	1.181029	2.808222	4.741278	5.760699
Pap	1.173799	2.707146	4.605701	5.847765
Pap	1.247650	2.764226	4.686423	6.085211
Pap	1.270501	2.788883	4.571060	5.859218
Pap	1.257060	2.666030	4.575861	5.347578
CytC	0.773775	1.154671	1.338510	1.613270
CytC	0.788500	1.156387	1.367564	1.763172
CytC	0.780572	1.206128	1.547302	1.597860
CytC	0.777637	1.174051	1.381252	1.671422

CytC	0.797948	1.201047	1.451372	1.646069
CytC	0.839867	1.178270	1.463809	1.882644
TRF	1.345599	1.760250	2.583712	3.025719
TRF	1.345558	1.896717	2.394805	2.992632
TRF	1.362604	1.981048	2.561250	3.165965
TRF	1.363887	1.801949	2.645955	3.391815
TRF	1.346467	2.771756	2.787455	3.009305
TRF	1.315298	1.856427	2.657281	3.428793
Mb	1.423610	2.835556	3.413458	3.466691
Mb	1.366895	2.829476	3.171333	3.844234
Mb	1.490389	2.472618	3.268913	3.770118
Mb	1.485235	2.651607	2.905632	3.735650
Mb	1.527541	2.609565	3.017456	3.420632
Mb	1.503684	2.549224	2.939047	2.898193
Lys	1.367519	2.052223	3.059410	2.809970
Lys	1.411762	2.049518	3.012569	2.825072
Lys	1.270080	2.095952	3.410823	3.593661
Lys	1.397748	2.280847	3.509867	3.768993
Lys	1.389064	2.283014	3.043140	3.507844
Lys	1.398495	2.322329	3.086964	3.524534
Lip	1.707785	4.496668	5.563588	6.114498
Lip	1.677598	4.454861	5.064093	6.306567
Lip	1.678943	4.595037	4.945882	5.639197
Lip	1.538811	4.527707	4.895552	5.865497
Lip	1.619397	4.524743	5.029589	6.023498
Lip	1.640304	4.335347	5.148796	5.505018
Try	1.536810	1.896642	2.586697	3.014454
Try	1.517450	2.026457	2.800288	2.734125
Try	1.498329	1.949709	2.720781	3.188577
Try	1.505229	1.833705	2.656598	3.069092
Try	1.533032	1.948781	2.957529	3.607141
Try	1.529906	1.925907	2.964968	3.271359
Hb	1.363274	2.864823	5.664173	9.789755
Hb	1.377243	2.734847	5.335470	9.239253
Hb	1.341775	2.699794	5.544515	9.465015
Hb	1.396231	2.730818	4.886299	9.160041
Hb	1.407039	2.781060	5.622898	9.291494
Hb	1.403799	2.872550	5.849061	8.776378

Supplementary Table 23. **The training matrix of fluorescence response patterns of SA8 against different proteins.**

Protein	SC5A-CD/LCG = 4:5	SC5A-CD/LCG = 1:1	SC5A-CD/LCG = 6:5	SC5A-CD/LCG = 7:5
HSA	7.497342	6.367029	16.574704	15.902075
HSA	7.507873	7.008830	17.236813	15.672961
HSA	8.030073	6.711394	18.181541	15.347014
HSA	7.740512	7.470438	18.217731	15.101253
HSA	7.989308	6.573981	18.963668	16.835130
HSA	7.470314	6.971243	16.399731	16.211817
BSA	6.612457	5.156270	14.945781	13.866114
BSA	6.729335	5.047799	17.175466	14.708101
BSA	6.676307	4.999652	16.508313	13.406965
BSA	6.673922	5.044154	17.093698	13.273116
BSA	6.591416	5.075784	15.305550	13.359959
BSA	6.450790	4.774627	13.871951	12.895429
BHb	7.840805	6.468374	16.762500	15.746493
BHb	7.379451	6.561805	17.411122	16.390173
BHb	7.729624	6.504149	17.719177	16.490669
BHb	7.424130	6.519093	20.673602	17.070291
BHb	7.526530	6.381483	17.221655	16.777491
BHb	6.986747	6.619325	17.532933	16.753950
Ova	4.497409	3.273636	5.367757	4.743760
Ova	4.510696	3.195864	5.289714	4.570389
Ova	4.572413	3.174909	5.496716	4.664802
Ova	4.707672	3.221916	5.012006	4.815730
Ova	4.585715	2.892264	5.073419	4.627700
Ova	4.232320	3.019937	5.285638	4.562545
Sal	12.721617	11.389316	23.830843	24.528156
Sal	12.739499	11.204427	26.315875	24.720438
Sal	12.117258	11.714437	24.143774	25.349505
Sal	12.200092	11.125177	25.320773	24.916732
Sal	13.262122	10.834752	24.385258	23.917652
Sal	12.448860	11.338299	23.439564	23.728992
Pap	6.965531	6.044013	16.467782	16.099727
Pap	6.794902	6.367793	16.677151	18.756066
Pap	7.043137	6.187389	16.954762	17.636497
Pap	6.770367	6.237591	16.304208	17.655953
Pap	6.973765	6.058631	16.909074	17.512397
Pap	7.171533	6.304021	16.136832	16.396895
CytC	9.177050	8.702905	24.059142	24.094648
CytC	9.267921	8.926478	24.388478	24.344522
CytC	9.090419	8.592833	23.694688	24.010268
CytC	9.204659	8.902520	24.382413	25.120266

CytC	8.741871	8.673065	24.718814	25.272667
CytC	9.123507	8.503051	23.255727	24.620240
TRF	4.481830	3.691176	8.226020	7.192185
TRF	4.666269	3.846015	8.487177	7.484400
TRF	4.721084	3.991119	8.155916	7.969942
TRF	4.834850	3.720785	8.909739	7.491125
TRF	4.601567	4.079095	8.731327	7.821304
TRF	4.983101	3.910605	8.917817	8.170583
Mb	8.041881	6.554120	19.708243	17.846216
Mb	7.908467	6.816568	20.077486	17.662596
Mb	8.134289	6.800331	20.843335	17.993091
Mb	7.760035	6.784963	20.020781	17.366231
Mb	7.986895	6.610510	19.236812	16.645083
Mb	7.657873	6.469564	18.314784	16.662471
Lys	6.124135	4.656084	7.372401	4.767203
Lys	5.606070	4.480701	7.120326	5.095970
Lys	5.769102	4.579660	7.596311	4.797832
Lys	5.408224	4.504025	7.943722	4.918807
Lys	5.390729	4.458563	7.704552	5.150639
Lys	6.048771	4.712556	7.293547	5.463943
Lip	2.169849	2.067197	2.567481	2.467263
Lip	2.090218	2.137867	2.466124	2.262419
Lip	2.082835	2.077199	2.700500	2.185136
Lip	2.089580	2.115264	2.559507	2.359254
Lip	2.075025	2.083679	2.726395	2.249341
Lip	2.096279	2.106437	2.768335	2.305407
Try	5.420597	5.183314	11.640027	8.698508
Try	5.330532	5.184896	11.588652	9.176234
Try	5.601744	5.238911	12.511682	9.707982
Try	5.630729	5.182403	12.326269	9.937802
Try	5.613294	5.150553	12.413937	9.204654
Try	5.580031	5.164303	12.431427	8.992934
Hb	8.793367	8.778343	21.036847	16.026736
Hb	8.914490	8.884986	20.942178	17.309193
Hb	8.757517	9.140539	19.773670	17.707052
Hb	8.599462	9.243655	21.617724	17.293195
Hb	8.912107	9.235790	20.406895	16.322694
Hb	8.501051	8.866342	21.426964	16.965523

Supplementary Table 24. **The training matrix of fluorescence response patterns of SA9 against different proteins.**

Protein	GC5A-CD/AlPcS$_4$ pH = 3	GC5A-CD/AlPcS$_4$ pH = 5	GC5A-CD/AlPcS$_4$ pH = 7	GC5A-CD/AlPcS$_4$ pH = 9
HSA	5.326917	2.134155	2.288161	3.185845
HSA	4.618647	1.979932	2.266455	3.069854
HSA	5.393915	2.020149	2.647892	3.415315
HSA	5.149410	2.137698	2.421934	3.032033
HSA	5.029443	2.145269	2.322732	2.988314
HSA	5.301921	2.382472	2.249340	3.572906
BSA	8.405291	4.779226	5.697730	8.171182
BSA	9.934466	4.858337	6.445767	7.613577
BSA	8.419119	4.903098	5.618552	7.479425
BSA	7.819043	4.360310	4.887231	7.735216
BSA	7.947442	4.631465	5.223062	7.451328
BSA	7.634064	4.513549	5.629877	6.785471
BHb	1.463827	1.819313	2.419470	4.452652
BHb	1.442704	1.609214	2.421357	3.911859
BHb	1.660589	2.172025	2.143168	4.224189
BHb	1.534109	2.172025	2.183094	4.186682
BHb	1.513148	1.860366	2.326729	4.414134
BHb	1.350684	1.932373	2.133173	4.088360
Ova	2.913767	4.583513	3.327700	3.217763
Ova	2.345976	4.094989	3.215941	4.265061
Ova	2.273823	3.888308	3.380508	3.721267
Ova	2.193575	4.180681	3.597541	4.240621
Ova	2.519081	5.042198	3.458196	3.992430
Ova	2.401407	4.262118	3.813440	4.108717
Sal	6.074602	7.443798	4.481002	9.343038
Sal	6.554984	7.662934	3.739017	11.623272
Sal	6.252805	6.433471	4.977833	11.062795
Sal	6.682785	7.322225	5.494690	11.043662
Sal	7.020955	6.911532	5.405192	10.186684
Sal	6.264910	7.180646	5.676433	12.557062
Pap	2.070150	3.596187	2.833662	3.850943
Pap	2.312305	3.374271	3.041077	3.963312
Pap	2.585494	3.646196	3.184981	4.287096
Pap	2.506319	3.751474	2.838127	4.192110
Pap	2.831039	3.701655	3.029950	3.993573
Pap	2.535074	3.788856	3.391454	4.298779
CytC	1.325482	1.586304	1.289890	2.017643
CytC	1.136976	1.297785	1.370877	1.865871
CytC	1.160197	1.563558	1.326395	1.948825
CytC	1.202960	1.327659	1.415169	1.807421

CytC	1.098402	1.579701	1.297863	1.995578
CytC	1.229197	1.408061	1.354161	1.886907
TRF	2.129491	1.441332	1.786839	2.495940
TRF	2.186098	1.360721	1.572179	2.746636
TRF	2.343427	1.315414	1.665205	2.544061
TRF	2.396993	1.912741	1.429236	2.810896
TRF	2.259771	1.293243	1.933606	2.636076
TRF	2.061027	1.554640	1.561085	2.280652
Mb	1.714091	1.720970	2.275333	2.833400
Mb	1.689368	1.667191	2.004737	2.773324
Mb	1.512370	1.718034	2.052336	2.548482
Mb	1.636771	1.651627	2.170118	3.049028
Mb	1.537991	1.565569	2.006920	2.966855
Mb	1.563569	1.649539	2.019524	2.795065
Lys	1.661121	1.393871	1.416946	1.955586
Lys	1.456779	1.388214	1.466016	1.987544
Lys	1.499527	1.328404	1.501277	2.047447
Lys	1.564684	1.299186	1.469272	1.967668
Lys	1.353793	1.517488	1.265080	1.911472
Lys	1.524417	1.253010	1.443312	1.946226
Lip	1.189932	1.596816	3.201648	5.011445
Lip	1.188164	1.701585	3.094589	5.095119
Lip	1.246273	1.701548	3.143534	5.016555
Lip	1.273247	1.851976	3.054540	4.906779
Lip	1.142067	1.760696	3.064057	5.201445
Lip	1.229000	2.012610	3.084860	4.773262
Try	2.326593	2.153029	1.770583	2.130260
Try	2.470204	2.186979	1.711427	2.218903
Try	2.330759	2.056610	1.787048	2.157045
Try	2.309494	2.136342	1.545638	2.295455
Try	2.315097	1.872570	1.706943	2.176299
Try	2.238211	2.022585	1.801711	2.399277
Hb	1.390131	1.899236	2.360282	6.925563
Hb	1.293657	2.020024	2.477242	7.506374
Hb	1.445875	1.923990	2.537635	6.758912
Hb	1.401346	2.141857	2.511607	7.069411
Hb	1.535869	1.985657	2.434387	7.410768
Hb	1.479594	2.124727	2.362679	7.136199

Supplementary Table 25. The training matrix of fluorescence response patterns of SA10 against different proteins.

Protein	SC5A-CD/LCG pH = 5	SC5A-CD/LCG pH = 7	SC5A-CD/LCG pH = 9	SC5A-CD/LCG pH = 11
HSA	3.072455	2.619609	2.792605	1.368076
HSA	3.134991	2.619762	3.037307	1.265345
HSA	3.183285	2.545274	3.288775	1.213438
HSA	3.134120	2.723982	2.840514	1.218965
HSA	3.242206	2.832302	3.147569	1.247611
HSA	3.121040	2.588567	3.082063	1.321765
BSA	3.098238	2.337291	2.433621	1.254445
BSA	3.085041	2.289261	2.491431	1.288917
BSA	3.139369	2.387448	2.735916	1.246320
BSA	3.110067	2.433695	2.693786	1.336789
BSA	3.155606	2.562468	2.744554	1.265272
BSA	3.046772	2.049725	2.432623	1.220866
BHb	2.751573	2.706694	3.137626	1.507631
BHb	2.695584	2.615463	3.035006	1.476962
BHb	2.731130	2.556015	2.950345	1.556510
BHb	2.688240	2.950275	3.116409	1.466658
BHb	2.591286	2.736227	3.120798	1.486183
BHb	2.538344	2.442847	3.026056	1.412819
Ova	2.171773	2.124953	2.389492	1.547049
Ova	2.285227	2.076505	2.548226	1.387823
Ova	2.297488	2.089176	2.569918	1.483044
Ova	2.324815	2.005781	2.594774	1.547830
Ova	2.267178	2.104969	2.506335	1.605873
Ova	2.230935	2.134701	2.523832	1.550980
Sal	3.143119	4.484511	4.792191	6.017385
Sal	3.178977	4.337455	4.574553	5.624544
Sal	3.134073	4.259754	4.655700	6.472454
Sal	3.134162	4.387917	4.691648	5.662474
Sal	3.118743	4.095847	4.457690	5.994120
Sal	3.078612	4.459924	4.730739	6.437456
Pap	2.629649	3.014833	3.151671	2.967618
Pap	2.573007	3.266429	3.219169	3.104067
Pap	2.709523	3.154539	3.491568	2.807267
Pap	2.680868	3.516435	3.406626	3.091876
Pap	2.661594	3.462699	3.486756	3.128311
Pap	2.731616	3.314890	3.350562	3.161413
CytC	3.252857	3.957805	3.785908	4.011063
CytC	3.276746	4.281837	3.830498	4.014426
CytC	3.324189	4.023603	3.804164	3.861687
CytC	3.264440	4.208393	3.858128	3.995948

CytC	3.184010	4.263759	3.748332	4.107186
CytC	3.202048	4.169599	3.670835	4.471375
TRF	2.485562	2.143849	2.383189	1.235332
TRF	2.545745	2.278887	2.384772	1.303927
TRF	2.540058	2.206506	2.125218	1.132510
TRF	2.502910	2.096162	2.244286	1.172612
TRF	2.543542	2.169974	2.426224	1.276866
TRF	2.442880	2.122534	2.281615	1.268660
Mb	2.971522	2.771021	3.379515	1.426739
Mb	3.035216	2.706422	3.249131	1.478561
Mb	3.044762	2.777594	3.283448	1.367988
Mb	3.028957	2.928380	3.419005	1.324750
Mb	3.032998	2.753313	3.451655	1.267412
Mb	2.923523	2.733957	2.941885	1.322970
Lys	2.264199	2.437643	2.427159	1.712102
Lys	2.369328	2.518394	2.727390	1.808069
Lys	2.464212	2.435352	2.896303	1.829943
Lys	2.562621	2.557351	2.832174	1.779085
Lys	2.484903	2.452088	2.956070	1.905111
Lys	2.516459	2.471059	2.664514	1.620237
Lip	1.742886	1.734575	1.709801	1.647962
Lip	1.750990	1.549051	1.580840	1.677084
Lip	1.716434	1.665824	1.691229	1.596331
Lip	1.725375	1.762102	1.786840	1.598745
Lip	1.694161	1.693256	1.629104	1.748745
Lip	1.729342	1.584541	1.737875	1.680313
Try	2.384420	2.902920	2.490035	1.224529
Try	2.346349	2.538553	2.472764	1.120733
Try	2.354492	2.592687	2.448603	1.161813
Try	2.384267	2.826168	2.514681	1.271548
Try	2.356584	2.489732	2.722421	1.226639
Try	2.367427	2.490191	2.523946	1.306049
Hb	2.352651	3.669376	3.392824	1.994030
Hb	2.421508	3.781095	3.167123	1.811838
Hb	2.432565	3.720219	3.302760	1.932781
Hb	2.327913	3.586042	3.254717	2.132362
Hb	2.385247	4.473158	3.084503	2.124857
Hb	2.422527	4.024747	3.005709	2.051033

Supplementary Table 26. The training matrix of fluorescence response patterns of SA1 against CytC and Ova at (1) 2.4, (2) 3.6, (3) 4.8, (4) 14.3, (5) 71.4 and (6) 238.1 μg/mL.

Protein	GC5A-CD/$AlPcS_4$	QC5A-CD/$AlPcS_4$	SC4A-CD/LCG	SC5A-CD/LCG
CytC 1	1.025433	1.265644	2.926344	3.414976
CytC 1	1.123462	1.255372	3.186766	3.349424
CytC 1	1.085995	1.282035	3.332035	3.295259
CytC 1	1.039614	1.294608	3.423120	3.429066
CytC 1	1.089727	1.258596	3.293169	3.790566
CytC 1	1.046268	1.389083	3.327486	3.398300
CytC 2	1.088854	1.341159	4.425719	4.332748
CytC 2	1.073708	1.345819	4.779981	4.263260
CytC 2	1.175200	1.352692	4.576537	4.426817
CytC 2	1.084156	1.166900	4.487855	4.267315
CytC 2	1.103503	1.432913	4.496509	4.399876
CytC 2	1.130333	1.330723	4.439075	4.294275
CytC 3	0.978867	1.347084	6.330539	5.031903
CytC 3	0.961754	1.293313	5.973701	5.017016
CytC 3	0.870169	1.279046	6.432699	5.024917
CytC 3	0.850500	1.260292	6.049224	5.036743
CytC 3	0.920903	1.218951	5.632368	4.908210
CytC 3	0.926743	1.281075	5.854841	4.828073
CytC 4	0.836047	1.099639	8.078540	5.701697
CytC 4	0.840537	1.147909	8.311619	6.002186
CytC 4	0.778800	1.123291	8.290928	5.891925
CytC 4	0.870428	1.040162	8.435408	5.827902
CytC 4	0.826220	1.200097	7.820955	5.792909
CytC 4	0.836365	1.081140	7.961593	5.723384
CytC 5	0.620951	0.886543	5.809173	6.521413
CytC 5	0.634839	0.824080	6.294582	6.436563
CytC 5	0.598401	0.907647	6.226718	6.200777
CytC 5	0.680297	0.841530	5.878856	6.592259
CytC 5	0.656945	0.878991	5.971869	6.629568
CytC 5	0.640065	0.852638	6.371362	6.888065
CytC 6	0.530227	0.703997	5.850497	5.572668
CytC 6	0.571589	0.685836	5.785039	5.528625
CytC 6	0.508730	0.753692	5.568435	5.834054
CytC 6	0.553902	0.804439	5.410312	5.674830
CytC 6	0.569912	0.804613	5.524298	5.421642
CytC 6	0.550899	0.795088	5.455967	5.699142
Ova 1	2.924518	1.762618	1.478451	1.394597
Ova 1	2.757982	1.732567	1.449545	1.298248
Ova 1	2.920688	1.799799	1.409471	1.351744
Ova 1	2.685538	1.741349	1.449951	1.291186

Ova 1	2.916078	1.769547	1.445436	1.292312
Ova 1	3.048083	1.761528	1.434176	1.428763
Ova 2	3.441133	2.069749	1.466601	1.446127
Ova 2	3.573661	2.035195	1.506851	1.586084
Ova 2	3.575866	2.061522	1.463233	1.451017
Ova 2	3.568112	2.081648	1.485143	1.513603
Ova 2	3.577015	2.020691	1.460500	1.420397
Ova 2	3.598666	2.094514	1.408488	1.461249
Ova 3	3.761465	2.427506	1.478910	1.485780
Ova 3	3.969460	2.347823	1.499845	1.525484
Ova 3	3.714099	2.329009	1.455563	1.461234
Ova 3	3.881586	2.287699	1.503839	1.508127
Ova 3	3.877693	2.380575	1.473322	1.555256
Ova 3	3.916039	2.332847	1.479354	1.490527
Ova 4	4.892352	2.716085	1.702152	1.869216
Ova 4	4.699522	2.800326	1.739089	1.930461
Ova 4	4.728519	2.785885	1.711934	1.864685
Ova 4	4.665796	2.827733	1.731444	1.877606
Ova 4	4.431053	2.745661	1.783424	1.840649
Ova 4	4.593677	2.745641	1.671922	1.815132
Ova 5	6.239503	3.351705	2.039888	2.590922
Ova 5	6.278850	3.369049	2.039720	2.658390
Ova 5	6.340933	3.313760	1.931311	2.552551
Ova 5	6.454094	3.483053	1.833791	2.388179
Ova 5	6.523123	3.464073	1.900505	2.438497
Ova 5	5.969754	3.415954	1.870243	2.360663
Ova 6	8.050953	4.576525	2.284687	2.720022
Ova 6	8.557471	4.729394	2.251363	2.621083
Ova 6	8.497551	4.504987	2.107862	2.636051
Ova 6	8.041523	4.724944	2.217713	2.938766
Ova 6	8.218970	4.536609	2.158779	2.852766
Ova 6	8.171375	4.516265	2.073772	2.649540

Supplementary Table 27. The training matrix of fluorescence response patterns of SA1 against protein mixtures of BSA/BHb.

Protein	GC5A-CD/AlPcS$_4$	QC5A-CD/AlPcS$_4$	SC4A-CD/LCG	SC5A-CD/LCG
100% BSA	4.161138	3.131978	3.478157	3.142999
100% BSA	3.993168	3.267009	3.482880	3.163562
100% BSA	4.007485	3.067493	3.405164	3.204847
100% BSA	4.399874	3.323011	3.593531	3.099333
100% BSA	4.175657	3.206452	3.226466	3.124312
100% BSA	4.440726	3.428315	3.276223	3.080576
60% BSA + 40% BHb	2.736873	2.678285	4.170173	3.875176
60% BSA + 40% BHb	3.207936	2.879972	4.166100	4.138991
60% BSA + 40% BHb	3.556130	2.768586	4.214483	4.136502
60% BSA + 40% BHb	3.603262	2.878856	4.248384	4.118125
60% BSA + 40% BHb	3.824257	2.789186	4.233779	4.268226
60% BSA + 40% BHb	3.906417	2.807990	4.049031	4.195249
40% BSA + 60% BHb	3.281755	2.615938	5.511640	4.378925
40% BSA + 60% BHb	3.127121	2.354255	5.816747	4.494242
40% BSA + 60% BHb	3.313075	2.483224	5.449602	4.514095
40% BSA + 60% BHb	3.244534	2.321612	5.467471	4.790430
40% BSA + 60% BHb	2.991020	2.256429	5.078216	4.684229
40% BSA + 60% BHb	2.725733	2.093530	4.841246	4.568757
100% BHb	1.738674	1.517609	6.958344	5.332981
100% BHb	1.904853	1.576827	6.507635	5.225577
100% BHb	1.876988	1.543568	6.248019	5.565511
100% BHb	1.780028	1.538433	5.966984	5.043929
100% BHb	1.707515	1.466744	6.098760	5.029503
100% BHb	1.583967	1.366912	6.182528	5.067074

Supplementary Table 28. **The training matrix of fluorescence response patterns of SA11 against honey samples from Tongrentang brand.**

Honey	SC4A-CD/LCG	SC5A-CD/LCG	SC6A-CD/LCG	SC8A-CD/LCG
Jujube	6.230147	5.003594	3.372409	2.305500
Jujube	7.199441	5.134370	3.493308	2.132304
Jujube	7.207394	5.093230	2.779037	2.212129
Jujube	7.048835	5.171314	3.419830	2.289788
Jujube	6.639463	5.087162	3.016777	2.302473
Jujube	6.935405	5.123528	3.462027	2.257741
Acacia	3.460042	3.067065	2.081567	1.429353
Acacia	3.252451	3.094244	1.978951	1.352735
Acacia	3.111643	2.936858	1.890374	1.422216
Acacia	3.725894	3.100851	2.003723	1.457782
Acacia	3.100808	3.018333	1.910316	1.404839
Acacia	3.180167	2.987388	1.951971	1.380433
Linden	4.749274	3.981333	2.625094	1.741893
Linden	4.562227	3.778193	2.495716	1.612298
Linden	4.900348	3.993108	2.561518	1.728867
Linden	4.257012	3.992587	2.538622	1.675919
Linden	4.893254	4.222766	2.720983	1.663390
Linden	5.037142	4.193794	2.792510	1.762204
Wolfberry	4.716913	4.553926	2.403738	1.568793
Wolfberry	4.123874	4.399806	2.467558	1.565561
Wolfberry	4.756908	4.523140	2.314494	1.503670
Wolfberry	4.299685	4.384690	2.326905	1.492018
Wolfberry	3.893081	4.344746	2.229061	1.537054
Wolfberry	4.518401	4.533318	2.410731	1.598943
Vitex	5.687258	4.130289	2.873543	2.368671
Vitex	6.286217	4.142682	2.906793	2.303441
Vitex	5.939762	4.210513	2.974227	2.450716
Vitex	5.608896	3.928243	2.848817	2.430456
Vitex	5.806297	4.212132	2.852477	2.223701
Vitex	5.898066	4.008708	2.947471	2.436466

Supplementary Table 29. **The training matrix of fluorescence response patterns of SA11 against honey samples from Wangshi brand.**

Honey	SC4A-CD/LCG	SC5A-CD/LCG	SC6A-CD/LCG	SC8A-CD/LCG
Jujube	7.151207	4.264315	3.122041	2.666385
Jujube	7.198389	4.296010	3.291996	2.818738
Jujube	6.881596	4.404520	3.154094	2.787289
Jujube	7.264417	4.327615	3.210931	2.791507
Jujube	7.225291	4.525636	3.162682	2.727560
Jujube	7.433260	4.287607	3.188632	2.693450
Acacia	3.452368	2.470234	1.737755	1.395822
Acacia	3.287888	2.421272	1.665319	1.328785
Acacia	3.280217	2.408927	1.694117	1.376040
Acacia	3.222106	2.352056	1.750071	1.344765
Acacia	3.114324	2.426483	1.765224	1.354130
Acacia	3.154164	2.344629	1.625462	1.308330
Motherwort	8.540696	4.312939	3.446782	1.891291
Motherwort	8.595089	4.449900	3.363265	1.832391
Motherwort	8.314447	4.815631	3.462903	1.761796
Motherwort	7.849573	4.803308	3.402172	1.813348
Motherwort	7.875328	4.868311	3.474134	1.885746
Motherwort	8.282824	4.682556	3.262339	1.890905
Coptis	5.335095	3.465826	2.450597	1.760567
Coptis	5.515045	3.526216	2.536175	1.840971
Coptis	5.053830	3.974815	2.739437	1.892285
Coptis	5.546779	3.253773	2.445726	1.750719
Coptis	5.542293	3.630695	2.673172	1.892357
Coptis	5.973783	3.761565	2.530033	1.953439
Multifloral	4.357228	3.156789	2.123035	1.476624
Multifloral	4.346653	3.528101	2.146547	1.500541
Multifloral	4.342390	2.831175	2.093424	1.413021
Multifloral	4.196058	2.851778	2.077669	1.423386
Multifloral	4.758000	3.051655	2.086192	1.462621
Multifloral	4.304611	3.147631	1.784616	1.472778

Supplementary Table 30. The training matrix of fluorescence response patterns of SA11 against jujube honey samples from four brands.

Honey	SC4A-CD/LCG	SC5A-CD/LCG	SC6A-CD/LCG	SC8A-CD/LCG
Tongrentang	6.230147	5.003594	3.372409	2.305500
Tongrentang	7.199441	5.134370	3.493308	2.132304
Tongrentang	7.207394	5.093230	2.779037	2.212129
Tongrentang	7.048835	5.171314	3.419830	2.289788
Tongrentang	6.639463	5.087162	3.016777	2.302473
Tongrentang	6.935405	5.123528	3.462027	2.257741
Guanshengyuan	4.806649	4.097928	2.728339	1.764731
Guanshengyuan	4.488110	3.607457	2.683880	1.772368
Guanshengyuan	5.061821	3.756969	2.795168	1.717269
Guanshengyuan	5.111172	3.571876	2.656157	1.762106
Guanshengyuan	5.693139	3.629921	2.801613	1.671754
Guanshengyuan	4.837522	3.901939	2.725830	1.773354
Wangshi	7.151207	4.264315	3.122041	2.666385
Wangshi	7.198389	4.296010	3.291996	2.818738
Wangshi	6.881596	4.404520	3.154094	2.787289
Wangshi	7.264417	4.327615	3.210931	2.791507
Wangshi	7.225291	4.525636	3.162682	2.727560
Wangshi	7.433260	4.287607	3.188632	2.693450
Baihua	3.479925	2.679020	2.176232	1.644962
Baihua	3.427182	2.576551	2.129227	1.567053
Baihua	3.573391	2.926366	2.145510	1.627045
Baihua	3.253452	2.508230	2.117013	1.551631
Baihua	3.480727	2.841444	2.245139	1.543166
Baihua	3.269592	2.744313	2.235569	1.567103

Supplementary Table 31. The training matrix of fluorescence response patterns of SA11 against mixtures of honey and syrup.

Honey	SC4A-CD/LCG	SC5A-CD/LCG	SC6A-CD/LCG	SC8A-CD/LCG
100% honey	8.540696	4.312939	3.446782	1.891291
100% honey	8.595089	4.449900	3.363265	1.832391
100% honey	8.314447	4.815631	3.462903	1.761796
100% honey	7.849573	4.803308	3.402172	1.813348
100% honey	7.875328	4.868311	3.474134	1.885746
100% honey	8.282824	4.682556	3.262339	1.890905
20% syrup + 80% honey	4.743742	4.144014	2.867572	1.704820
20% syrup + 80% honey	5.476567	4.230186	2.837277	1.646336
20% syrup + 80% honey	4.696267	4.168449	2.652899	1.720387
20% syrup + 80% honey	5.091580	4.124860	2.781755	1.528921
20% syrup + 80% honey	5.257049	4.143510	2.877765	1.702375
20% syrup + 80% honey	5.199196	4.161680	2.703831	1.668346
40% syrup + 60% honey	4.051036	3.321256	2.437531	1.653993
40% syrup + 60% honey	4.525192	3.461501	2.340372	1.468092
40% syrup + 60% honey	4.684341	3.361660	2.429168	1.592923
40% syrup + 60% honey	4.253777	3.303480	2.334560	1.614932
40% syrup + 60% honey	4.152110	3.373758	2.443014	1.515550
40% syrup + 60% honey	4.500780	3.394752	2.462761	1.704492
60% syrup + 40% honey	2.672866	2.736115	2.046995	1.465865
60% syrup + 40% honey	2.958787	2.862015	2.065233	1.453086
60% syrup + 40% honey	2.419065	2.802193	1.999724	1.386772
60% syrup + 40% honey	2.527919	2.759101	1.875950	1.328802
60% syrup + 40% honey	2.884132	2.736657	2.085393	1.345223
60% syrup + 40% honey	2.790310	2.651439	1.988326	1.051747
80% syrup + 20% honey	2.146678	2.130934	1.660281	1.359444
80% syrup + 20% honey	2.260030	2.230226	1.672414	1.401439
80% syrup + 20% honey	2.170595	2.166417	1.627531	1.375679
80% syrup + 20% honey	2.214064	2.117460	1.676653	1.287558
80% syrup + 20% honey	2.284350	2.115972	1.819865	1.288701
80% syrup + 20% honey	2.402431	2.284385	1.727925	1.251796
100% syrup	1.489528	1.410342	1.356036	1.213427
100% syrup	1.472173	1.594977	1.384297	1.122560
100% syrup	1.334349	1.490876	1.364524	1.187078
100% syrup	1.408467	1.516689	1.335316	1.228381
100% syrup	1.311364	1.455728	1.320828	1.205691
100% syrup	1.389428	1.543207	1.430979	1.344363

Supplementary Table 32. The training matrix of fluorescence response patterns of SA11 against mixtures of vitex and rapeseed honey.

Honey	SC4A-CD/LCG	SC5A-CD/LCG	SC6A-CD/LCG	SC8A-CD/LCG
100% vitex honey	5.687258	4.130289	2.873543	2.368671
100% vitex honey	6.286217	4.142682	2.906793	2.303441
100% vitex honey	5.939762	4.210513	2.974227	2.450716
100% vitex honey	5.608896	3.928243	2.848817	2.430456
100% vitex honey	5.806297	4.212132	2.852477	2.223701
100% vitex honey	5.898066	4.008708	2.947471	2.436466
40% rapeseed honey + 60% vitex honey	5.195246	3.640497	2.371081	2.166026
40% rapeseed honey + 60% vitex honey	5.203429	3.739125	2.417225	2.081267
40% rapeseed honey + 60% vitex honey	5.186229	3.635771	2.493755	2.034072
40% rapeseed honey + 60% vitex honey	5.090143	3.741253	2.394014	1.939864
40% rapeseed honey + 60% vitex honey	5.447306	3.774503	2.383728	2.065315
40% rapeseed honey + 60% vitex honey	5.172620	3.699205	2.345055	2.058376
80% rapeseed honey + 20% vitex honey	3.855815	2.982192	1.922127	1.773163
80% rapeseed honey + 20% vitex honey	3.785902	3.085185	1.988188	1.759088
80% rapeseed honey + 20% vitex honey	3.931494	3.037434	2.021868	1.748947
80% rapeseed honey + 20% vitex honey	4.218866	3.175509	2.075329	1.662860
80% rapeseed honey + 20% vitex honey	4.126591	3.168622	2.129739	1.730052
80% rapeseed honey + 20% vitex honey	4.345278	3.147843	2.017004	1.754542
100% rapeseed honey	3.264126	2.639111	1.693630	1.615073
100% rapeseed honey	3.396419	2.683291	1.822303	1.373338
100% rapeseed honey	3.039852	2.506034	1.726881	1.581106
100% rapeseed honey	3.088490	2.599184	1.795721	1.474802
100% rapeseed honey	3.124069	2.527477	1.745885	1.558579
100% rapeseed honey	3.002012	2.695128	1.757096	1.572585

Supplementary References

1. Gutsche, C. D. & Iqbal, M. *p*-tert-Butylcalix[4]arene. *Organic Syntheses* **68**, 234 (1990).
2. Stewart, D. R. & Gutsche, C. D. The one-step synthesis of *p*-tert-butylcalix[5]arene. *Org. Prep. Proced. Int.* **25**, 137–139 (1993).
3. Geng, W.-C. et al. A self-assembled white-light-emitting system in aqueous medium based on a macrocyclic amphiphile. *Chem. Commun.* **53,** 392–395 (2017).
4. Gao, J. et al. Biomarker displacement activation: a general host-guest strategy for targeted phototheranostics in vivo. *J. Am. Chem. Soc.* **140,** 4945–4953 (2018).
5. Shahgaldian, P., Coleman, A. W., Kuduvab, S. S. & Zaworotkob, M. J. Amphiphilic behavior of an apparently non-polar calixarene. *Chem. Commun.* **15**, 1968–1970 (2005).
6. Wang, K.-P., Chen, Y. & Liu, Y. A polycation-induced secondary assembly of amphiphilc calixarene and its multi-stimuli responsive gelation behavior. *Chem. Commun.* **51**, 1647–1649 (2015).
7. Tian, H.-W. et al. Supramolecular imaging of spermine in cancer cells. *Nanoscale* **13,** 15362–15368 (2021).
8. Shinkai, S., Mori, S., Koreishi, H., Tsubaki, T. & Manabe, O. Hexasulfonated calix[6]arene derivatives: a new class of catalysts, surfactants, and host molecules. *J. Am. Chem. Soc.* **108**, 2409–2416 (1986.)
9. Tian, H.-W., Pan, Y.-C. & Guo, D.-S. Assembly-enhanced molecular recognition of calix[6]arene. *Supramol. Chem.* **30,** 562–567 (2018).
10. Munch, J. H. & Gutsche, C. D. *p*-tert-Butylcalix[8]arene. *Organic Syntheses* **68**, 243 (1990).
11. Sayin, S. & Yilmaz, M., Synthesis and investigation of catalytic affinities of water-soluble amphiphilic calix[n]arene surfactants in the coupling reaction of some heteroaromatic compounds. *Tetrahedron* **72,** 6528–6535 (2016).
12. Mazzaglia, A., Donohue, R., Ravoo, B. J. & Darcy, R. Novel amphiphilic cyclodextrins: graft-synthesis of heptakis(6-alkylthio-6-deoxy)-β-cyclodextrin 2-oligo(ethylene glycol) conjugates and their ω-halo derivatives. *Eur. J. Org. Chem.* **2001**, 1715–1721 (2001).
13. Cao, F.-Y. et al. Fluorescence light-up AIE probe for monitoring cellular alkaline phosphatase activity and detecting osteogenic differentiation. *J. Mater. Chem. B* **4**, 4534–4541 (2016).
